# Investigations of alkynylbenziodoxole derivatives for radical alkynylations in photoredox catalysis

**DOI:** 10.3762/bjoc.14.103

**Published:** 2018-05-28

**Authors:** Yue Pan, Kunfang Jia, Yali Chen, Yiyun Chen

**Affiliations:** 1 Department of Chemistry, Shanghai University, 99 Shangda Road, Shanghai 200444, China; 2 State Key Laboratory of Bioorganic and Natural Products Chemistry, Centre for Excellence in Molecular Synthesis, Shanghai Institute of Organic Chemistry, University of Chinese Academy of Sciences, Chinese Academy of Sciences, 345 Lingling Road, Shanghai 200032, China

**Keywords:** acyl radical, alkyl radical, alkynylbenziodoxoles, photoredox catalysis, radical alkynylation

## Abstract

The alkynylbenziodoxole derivatives are recently developed alkynylation reagents in organic synthesis, which demonstrate excellent radical alkynylation reactivity in photoredox catalysis reactions. Herein we report the synthesis of alkynylbenziodoxole derivatives with difluoro, monofluoro, monomethoxy, and dimethoxy substitution on the benziodoxole moiety, and investigated their radical alkynylation reactivity for the first time. A series of mechanistic experiments were conducted to study the radical acceptor and oxidative quencher reactivity of alkynylbenziodoxoles, in which unsubstituted alkynylbenziodoxoles played balancing roles in both processes, while electron-rich benziodoxole derivatives demonstrate synthetic advantages in some cases.

## Introduction

The introduction of the alkynyl group to organic molecules is an important synthetic transformation in organic synthesis [1–4]. Recently, cyclic iodine(III) reagents (CIR)-substituted alkynes, alkynylbenziodoxoles, were developed with readily preparation and shelf-stableness [5–10]. The alkynylbenziodoxoles were first synthesized by the Ochiai group, and later studied by Waser and other groups for the use in electrophilic alkynylation reactions [11–18]. In 2012, the Li group first used alkynylbenziodoxoles for decarboxylative radical alkynylation under silver salt and persulfate conditions [19]. In 2014, the Chen group discovered that alkynylbenziodoxoles (BI-alkyne) readily participated in photoredox catalysis as the radical alkynylation reagent [20], after which various applications in photoredox catalysis were reported [21–27].

**Scheme 1:**
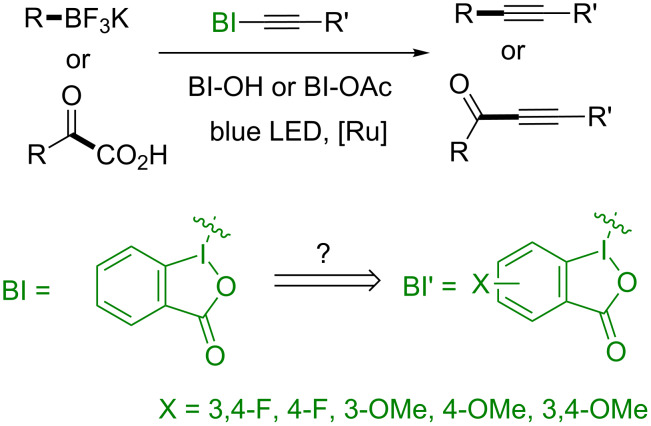
Investigation of alkynylbenziodoxole derivatives for radical alkynylations.

Currently, the use of BI-alkyne for radical alkynylation is limited to unsubstituted alkynylbenziodoxoles. While effective, its reactivity with some radical precursors was compromised [19–27]. The Waser group pioneered the study of substituted alkynylbenziodoxoles for the electrophilic alkynylation reactivity, however, no significant improvements were observed by the derivatizations [28–32]. Herein, we report the synthesis of alkynylbenziodoxole derivatives and investigate their reactivity toward alkyl radical and acyl radical additions in photoredox catalysis. The mechanistic investigations were carried out to study the derivatization of BI-alkynes in radical acceptor and oxidative quencher reactivity, and the electron-rich benziodoxole derivatives demonstrated synthetic advantages in some cases (Scheme 1).

## Results and Discussion

**Scheme 2:**
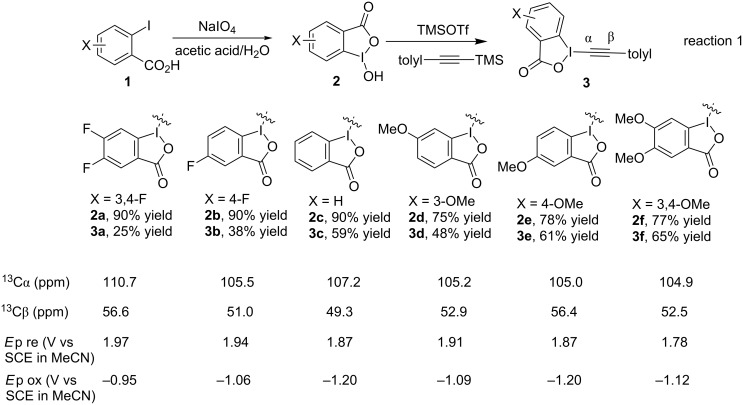
Synthesis and characterization of BI-alkyne derivatives **3a**–**f**.

We started the synthesis of BI-alkyne derivatives with substituted *o*-iodobenzoic acids **1** bearing 3,4-difluoro, 4-fluoro, 3-methoxy, 4-methoxy, or 3,4-dimethoxy substitutions (Scheme 2). Using a slightly modified Ochiai procedure [11], the substituted hydroxybenziodoxoles **2a**–**f** were prepared with periodate oxidation in 75–90% yield, in which the electronic effect did not have much influence on the reaction [33]. Subsequently, the treatment with trimethylsilyl *p*-tolylacetylene in the presence of trimethylsilyl trifluoromethanesulfonate afforded *p*-tolylacetylenic benziodoxoles **3a**–**f** in 25–65% yield, in which the electron-donating substitutions were beneficial for the reaction. The two-step synthesis of BI’-alkyne derivatives **3a**,**b**,**d**–**f** were in the range of 23–50% yield in gram scale, which was comparable to the synthesis of unsubstituted BI-alkyne **3c**.

The ^13^C NMR spectra of BI-alkynes **3a**–**f** were studied with the focus on the α-carbon, which position directly underwent α-radical addition [19]. The electron density of the α-carbon was decreased in **3a** with electron-deficient 3,4-difluoro groups on the benziodoxole, and was increased for **3f** with electron-donating 3,4-dimethoxy groups. Cyclic voltammetry measurements were also carried out for BI-alkynes **3a**–**f**, in which the reduction potential (*E*p re) indicated the electron-accepting capacity of the BI-alkynes. As expected, the reduction potential of **3a** was increased with electron-deficient 3,4-difluoro substituents on the benziodoxole, and was decreased for **3f** with electron-donating 3,4-dimethoxy groups. It is interesting to note that the effect of single substitution on benziodoxoles in **3b**, **3d** and **3e** was insignificant and sporadical in both ^13^C NMR spectroscopy and cyclic voltammetry experiments.

**Scheme 3:**
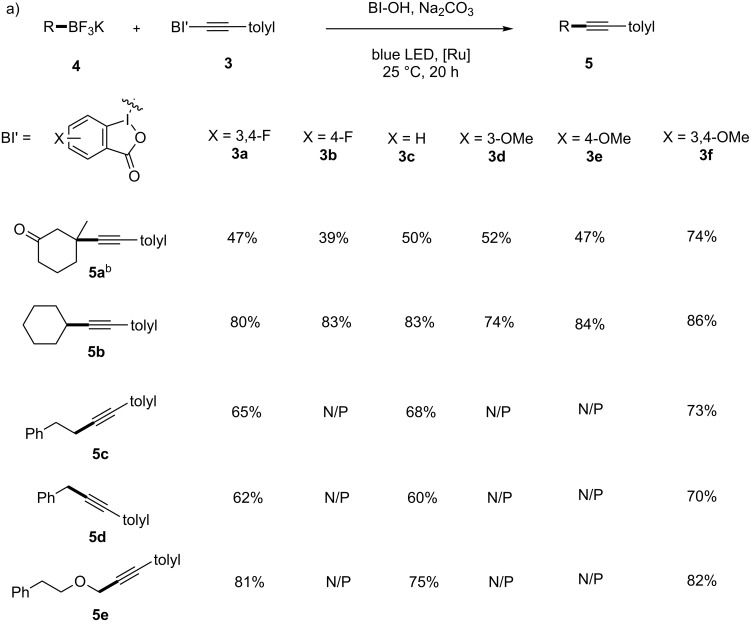
Reaction of alkynylbenziodoxole derivatives for deboronative alkynylation in photoredox catalysis. Reaction conditions: a) alkyl potassium trifluoroborate **4** (0.15 mmol, 1.5 equiv), alkynylbenziodoxole **3** (0.10 mmol, 1.0 equiv), Ru(bpy)_3_(PF_6_)_2_ (1.7 mg, 0.002 mmol, 0.02 equiv), hydroxybenziodoxole (BI-OH, 0.05 mmol, 0.5 equiv), and Na_2_CO_3_ (0.2 mmol, 2.0 equiv) in 1.0 mL CH_2_Cl_2_ and 1.0 mL H_2_O for 20 h under a nitrogen atmosphere, unless otherwise noted; b) **4** (0.3 mmol, 3.0 equiv), Na_2_CO_3_ (0.4 mmol, 4.0 equiv). Yields are isolated yields. N/P = not performed.

We next tested the reactivity of tolylacetylenic benziodoxole derivatives **3a**–**f** for deboronative alkynylation under photoredox catalysis conditions (Scheme 3) [20,34]. Using the tertiary alkyl trifluoroborate **4a** as the alkyl radical precursor under literature conditions, the unsubstituted BI-alkyne **3c** only results in 50% yield of alkynylation adduct **5a**, which is consistent with the literature report that tertiary alkyl trifluoroborates did not give satisfying results [20]. Using BI’-alkyne **3a** with 3,4-difluoro substitutions, the alkynylation adduct **5a** was obtained in decreased 47% yield. In contrast, 74% yield of **5a** was obtained with 3,4-dimethoxy-substituted BI’-alkyne **3f**. Being consistent with the ^13^C NMR spectroscopy and cyclic voltammetry experiments, the effect of single substitution on the benziodoxole was insignificant and no improvement was observed. We also tested the secondary alkyl trifluoroborate **4b** and primary alkyl trifluoroborate **4c**, in which the deboronative alkynylation with unsubstituted BI-alkynes already gave good results. The electronic effects on the benziodoxoles were less significant and fluctuated within the 5% yield range: The alkynylation adducts **5b** and **5c** were obtained in decreased 80% and 65% yields using BI’-alkyne **3a**, while 86% and 73% yields of **5b** and **5c** were obtained with BI’-alkyne **3f**. We then tested benzyl trifluoroborate **4d** and oxygen-substituted alkyl trifluoroborate **4e**, which were not reported for deboronative alkynylations before. The 3,4-dimethoxy-substituted BI’-alkyne **3f** gave the optimal 70% and 82% yields of alkynes **5d** and **5e**, which observed ≈10% yield improvement comparing to the unsubstituted BI-alkyne **3c**.

**Scheme 4:**
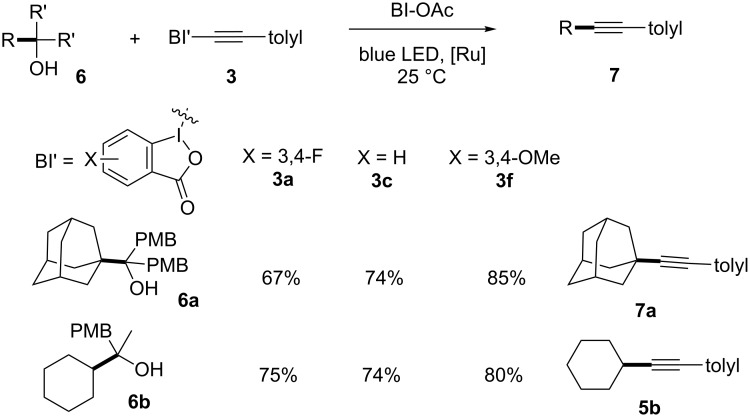
Reaction of alkynylbenziodoxole derivatives for radical alkynylations in photoredox catalysis. Reaction conditions: tertiary alcohol **6** (0.25 mmol, 2.5 equiv), alkynylbenziodoxole **3** (0.10 mmol, 1.0 equiv), Ru(bpy)_3_(PF_6_)_2_ (0.002 mmol, 0.02 equiv), and BI-OAc (0.25 mmol, 2.5 equiv) in 2.0 mL DCE for 24 h under a nitrogen atmosphere, unless otherwise noted. Yields are isolated yields.

We then tested if the propensity of BI-alkyne derivatives toward alkyl radical additions was general and could extend to other alkyl radical precursors (Scheme 4). Tertiary alcohols **6** were reported to be activated by cyclic iodine(III) reagents under photoredox conditions to generate alkoxyl radicals, and subsequently underwent β-fragmentation and alkynylation to yield **7** after eliminating the arylketone [25]. With tertiary alcohol **6a** as the alkyl radical precursor, the unsubstituted BI-alkyne **3c** gave 74% yield of **7a**, which was consistent with the literature report [25]. Under otherwise identical reaction conditions, 67% yield of **7a** was obtained with 3,4-difluoro BI’-alkyne **3a**, while optimal 85% yield of **7a** was obtained with 3,4-dimethoxy BI’-alkyne **3f**. We then tested the secondary alkyl radical precursor **6b** and observed 74% yield of alkyne **5b** using unsubstituted BI-alkyne **3c**. In contrast, 3,4-dimethoxy BI'-alkyne **3f** gave improved 80% yields of **5b**.

**Scheme 5:**
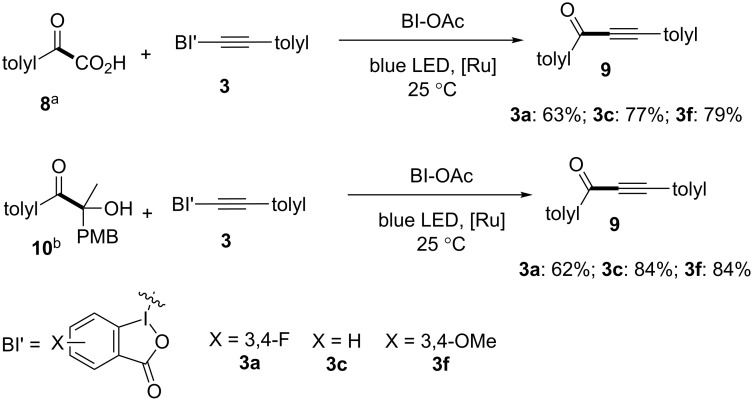
Reaction of alkynylbenziodoxole derivatives for acyl radical alkynylation in photoredox catalysis. Reaction conditions: a) ketoacid **8** (0.15 mmol, 1.5 equiv), alkynylbenziodoxole **3** (0.10 mmol, 1.0 equiv), Ru(bpy)_3_(PF_6_)_2_ (0.002 mmol, 0.02 equiv), and BIOAc (0.10 mmol, 1.0 equiv) in 2.0 mL DCM for 5 h under a nitrogen atmosphere; b) β-ketone alcohol **10** (0.20 mmol, 2.0 equiv), alkynylbenziodoxole **3** (0.10 mmol, 1.0 equiv), Ru(bpy)_3_(PF_6_)_2_ (0.002 mmol, 0.02 equiv), and BI-OAc (0.20 mmol, 2.0 equiv) in 2.0 mL DCM for 24 h under a nitrogen atmosphere. Yields are isolated yields.

We finally moved to test the BI-alkyne derivatives toward acyl radical additions (Scheme 5). With ketoacid **8** as the acyl radical precursor, the decarboxylative alkynylation with BI-alkyne derivatives afforded ynone **9** under the photoredox conditions [21]. Both the unsubstituted and 3,4-dimethoxy substituted BI’-alkynes **3c** and **3f** gave ynone **9** in similar 77–79% yields, while the 3,4-difluoro substituted BI’-alkyne **3a** gave a slightly lower 63% yield of **9** [21]. β-Ketone alcohols **10** were reported to be activated by cyclic iodine(III) reagents under photoredox conditions to generate alkoxyl radicals, and subsequently underwent β-fragmentation and alkynylation to yield ynone **9** [26]. The unsubstituted BI-alkyne **3c** gave 84% yield of **9** consistent with the literature report, while 62% yield of **9** was obtained with 3,4-difluoro BI’-alkyne **3a** and 84% yield of **9** was obtained with 3,4-dimethoxy BI’-alkyne **3f**.

**Scheme 6:**
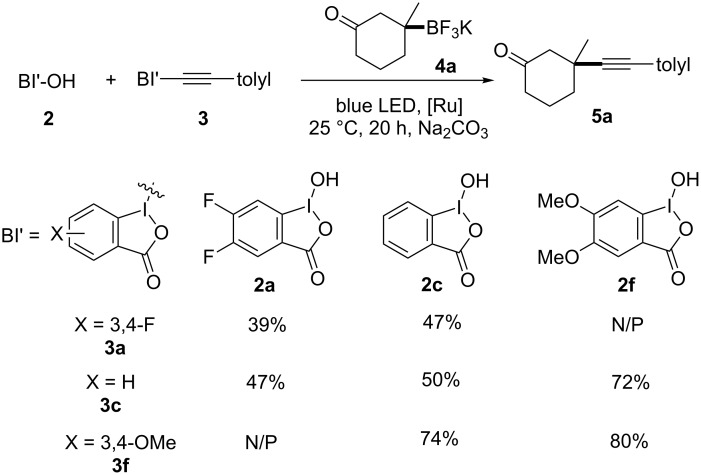
Mechanistic investigations of alkynylbenziodoxole for radical acceptor and oxidative quenching reactivity. Yields are isolated yields. N/P = not performed.

With the preliminary hypothesis that the electron-withdrawing and electron-donating substituents on the benziodoxole have opposite effects for the radical alkynylation, we first conducted the fluorescence quenching experiments of tolylacetylenic benziodoxole derivatives **3a**–**f** and found none of them oxidatively quenched the photoexcited Ru(bpy)_3_^2+*^ complex (see Supporting Information File 1, Scheme S1). We next investigated if the benziodoxole radical released from the radical alkynylation of BI-alkynes affected the reaction (Scheme 6). Using the combination of substituted hydroxybenziodoxoles (BI’-OH) and substituted BI’-alkynes, we found the 3,4-difluoro electron-withdrawing substituents either on BI’-OH or BI’-alkyne decreased the reaction yields, while the use of both further decreased the formation of **5a** to 39% yield. In contrast, the use of electron-donating 3,4-dimethoxy group either BI'-OH or BI'-alkyne increased the yields of **5a** to 74% and 72% yields, while the use of both increased the formation of **5a** to optimal 80% yield.

**Scheme 7:**
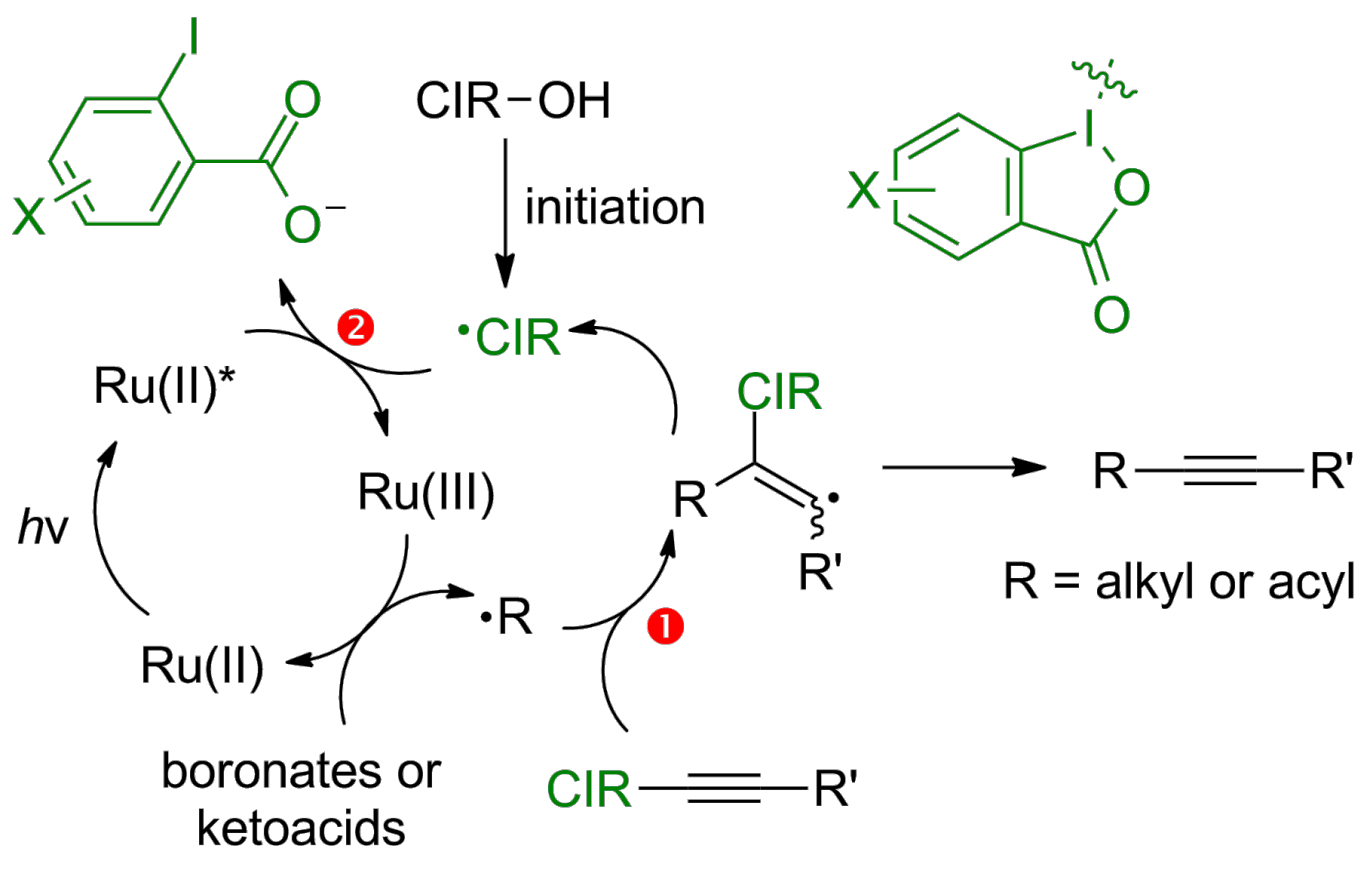
The role of alkynylbenziodoxole derivatives for radical alkynylation in photoredox catalysis.

Based on mechanistic investigations above, we propose that the electronic effect on benziodoxoles affected both the radical acceptor and oxidative quencher reactivity of BI-alkyne derivatives (Scheme 7). In the alkyl or acyl radical addition step to BI’-alkyne (step 1) and the oxidative quenching step by benziodoxole radical (step 2), the electron-donating substituents on BI’-alkynes are both beneficial, while the electron-withdrawing substituents have opposite effects.

## Conclusion

In conclusion, we have developed and investigated novel alkynylbenziodoxole derivatives as radical alkynylation reagents in photoredox catalysis reactions. Alkynylbenziodoxole derivatives with electron-rich benziodoxoles demonstrate synthetic advantages in some situations. The mechanistic investigations suggested both the radical acceptor (step 1) and oxidative quencher reactivity (step 2) were affected by BI-alkyne derivatization. We envision these alkynylbenziodoxole derivatives will provide alternative radical alkynylation reagents in photoredox catalysis and other synthetic applications.

## Supporting Information

File 1 Experimental details, and copies of ^1^H NMR and ^13^C NMR spectra for all new compounds.
