# Supplementary material for: Investigations of alkynylbenziodoxole derivatives for radical alkynylations in photoredox catalysis
Source: Beilstein J Org Chem. 2018 May 28;14:1215–21. doi: 10.3762/bjoc.14.103 (PMC6009179; doi:10.3762/bjoc.14.103)
Supplement: File 1 — Experimental details, and copies of 1H NMR and 13C NMR spectra for all new compounds. [file Beilstein_J_Org_Chem-14-1215-s001.pdf]

Supporting Information

for

Investigations of alkynylbenziodoxole derivatives for radical alkynylations in photoredox catalysis

Yue Pan[1,2], Kunfang Jia[2], Yali Chen*[1], and Yiyun Chen*[1,2]

Address: [1]Department of Chemistry, Shanghai University, 99 Shangda Road, Shanghai 200444 China and [2]State Key Laboratory of Bioorganic and Natural Products Chemistry, Centre for Excellence in Molecular Synthesis, Shanghai Institute of Organic Chemistry, University of Chinese Academy of Sciences, Chinese Academy of Sciences, 345 Lingling Road, Shanghai 200032 China

Email: Yali Chen - ylchen@staff.shu.edu.cn; Yiyun Chen - yiyunchen@sioc.ac.cn

*Corresponding author

Experimental details, and copies of ^{1}H NMR and ^{13}C NMR spectra for all new compounds

I. General methods. S2

II. Mechanistic studies S3

III. Substrates preparation and characterization S8

IV. Radical alkynylation product characterization S18

V. Reference S24

VI. NMR Spectra of new compounds S25

I. General methods.

Unless otherwise noted, all reactions of substrates preparation were conducted in flame-dried glassware under a nitrogen atmosphere using anhydrous solvent passed through an activated alumina column (Innovative Technology). Commercially available reagents were used without further purification. Thin layer chromatography (TLC) was performed using Jiangyou TLC silica gel plates HSG F_{254} and visualized using UV light, anisaldehyde or potassium permanganate. Flash chromatography was performed on Lisure science EZ purification system using the Santai technologies silica gel cartridge. Photochemical reactions were carried with 4 W blue LED (468 nm peak wavelength, 25 nm spectral half-wave width, composed of 55–65 LED units each with 60 mW, 3 V, 20 mA) obtained from Qiding Photo Electric (analyzed by Everfine PMS-50). ^{1}H and ^{13}C NMR spectra were recorded in $CDCl_3$ or $(CD_3)_2SO$, unless otherwise noted, on a Bruker AV-400 MHz or an Agilent 500 MHz spectrometer. Chemical shifts in ^{1}H NMR spectra were reported in parts per million (ppm) on the δ scale from an internal standard of residual chloroform (7.26 ppm) or dimethyl sulfoxide (2.50 ppm). Data for ^{1}H NMR were reported as follows: chemical shift, multiplicity (s = singlet, d = doublet, t = triplet, q = quartet, m = multiplet), coupling constant in Hertz (Hz) and integration. Data for ^{13}C NMR spectra were reported in terms of chemical shift in ppm from the central peak of $CDCl_3$ (77.16 ppm) or $(CD_3)_2SO$ (39.52 ppm). IR (neat) spectra were recorded on a Thermo Scientific Nicolet 380 FT-IR (neat) spectrometer. MS experiments were performed on a Bruker maXis 4G instrument for HRMS–ESI, an Agilent 5973N instrument for EI-MS, and a Waters Micromass GCT Premier instrument for HRMS–EI. Cyclic voltammetry experiments were performed on a CH Instruments Electrochemical Analyzer.

II. Mechanistic studies

The Luminescence quenching experiments

Emission intensities were recorded using Microplate Accessory 5JO-0139 spectrometer for all experiments. All $Ru(bpy)_3(PF_6)_2$ solutions were excited at 460 nm and the emission intensity at 600 nm was observed. In a typical experiment, the DCM solution of $Ru(bpy)_3(PF_6)_2$ (36 μM) was added the appropriate amount of quencher in a screw-top 1.0 cm quartz cuvette. After degassing with nitrogen for 10 min, the emission spectra of the samples were collected.

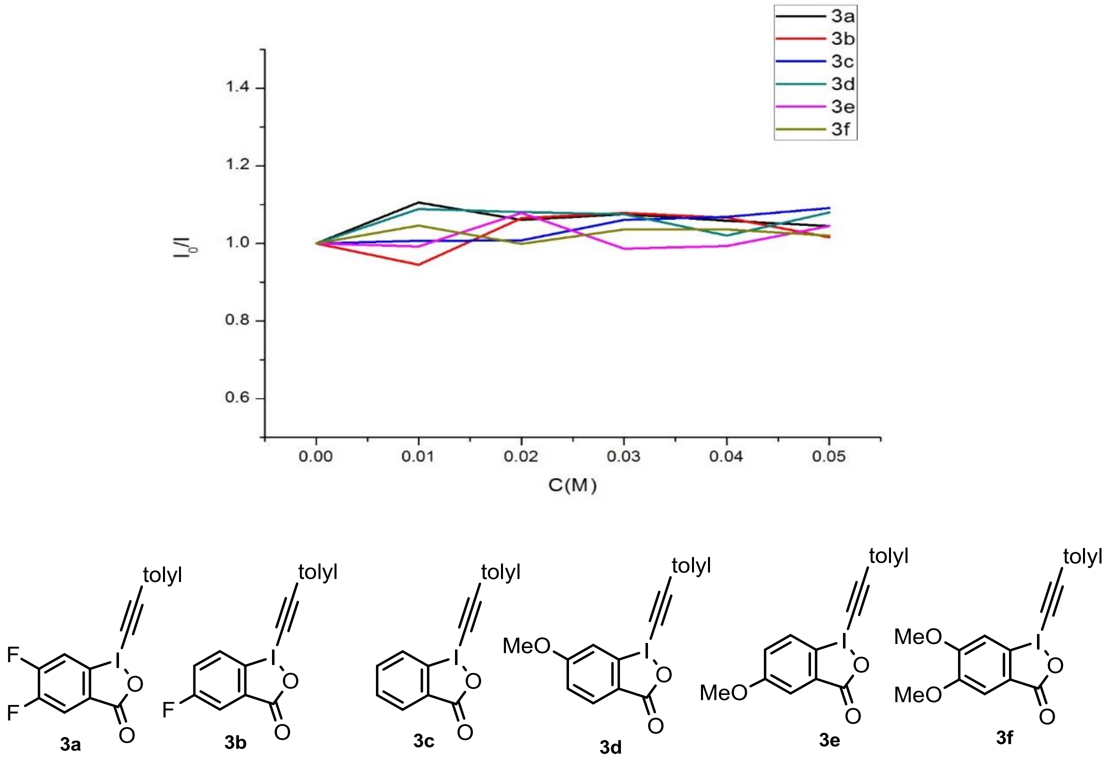

Scheme S1: $Ru(bpy)_3(PF_6)_2$ emission quenching by BI-alkyne

The cyclic voltammetry experiments

Cyclic voltammetry experiments were performed on a CH Instruments Electrochemical Analyzer at room temperature under a nitrogen atmosphere. The 1mM CH_3CN solution was prepared with 100 mM tetrabutylammonium tetrafluoroborate as the supporting electrolyte, using a glassy carbon working

electrode, a Pt counter electrode, and a Hg/$HgCl_2$ reference electrode. Scan Rate (V/s) = 0.05.

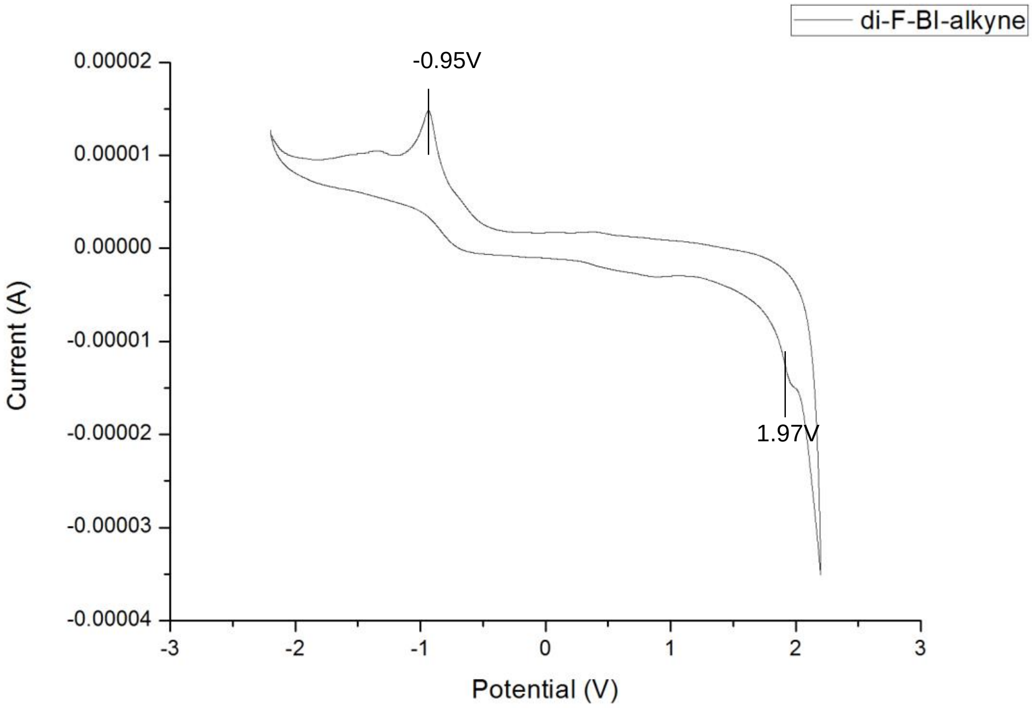

Scheme S2: The cyclic voltammetry experiment of 3a.

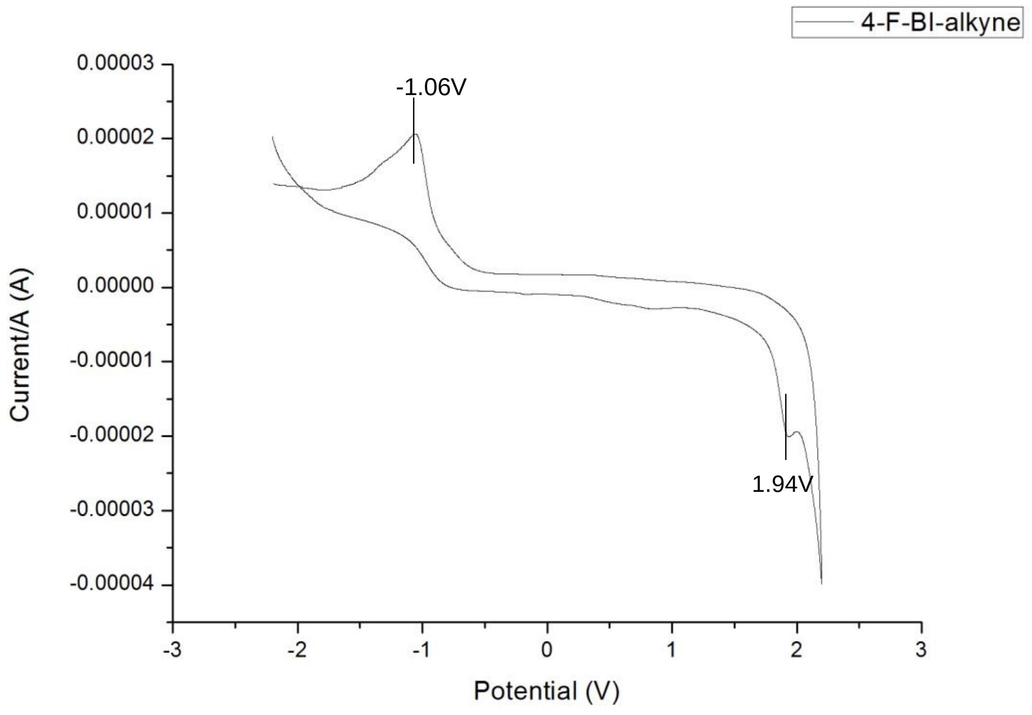

Scheme S3: The cyclic voltammetry experiment of 3b.

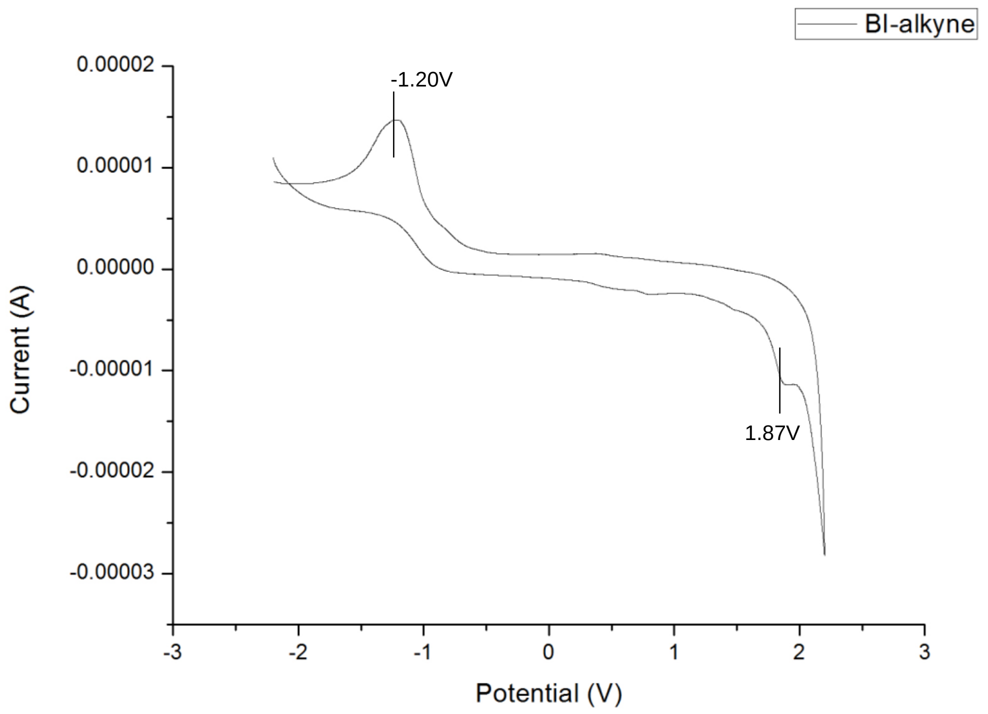

Scheme S4: The cyclic voltammetry experiment of 3c.

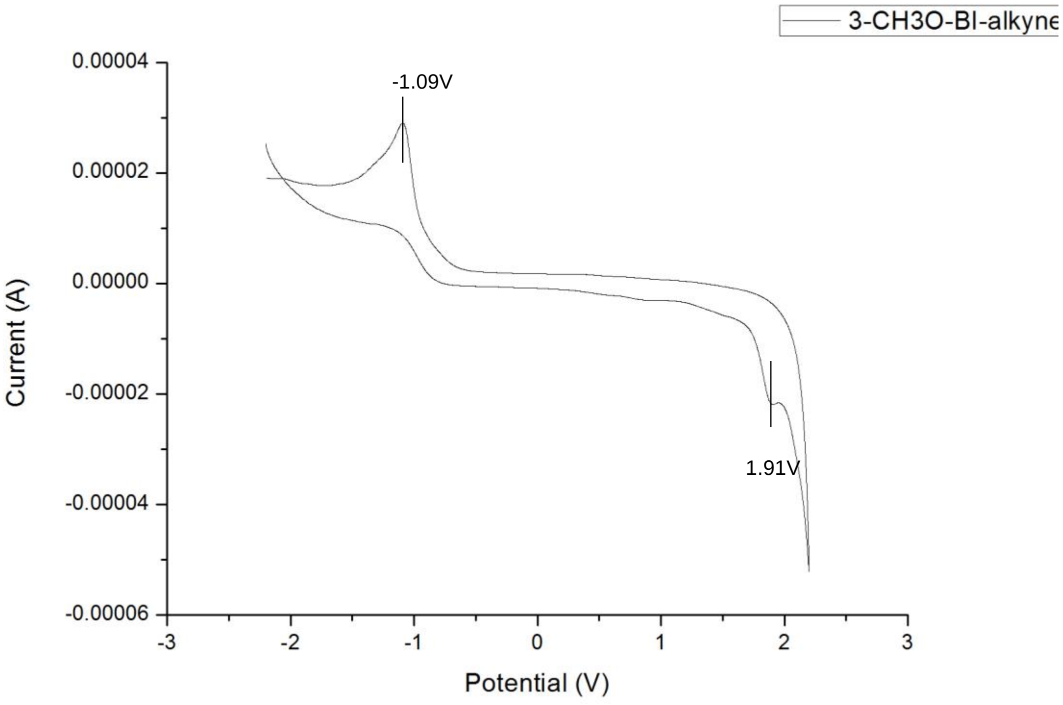

Scheme S5: The cyclic voltammetry experiment of 3d.

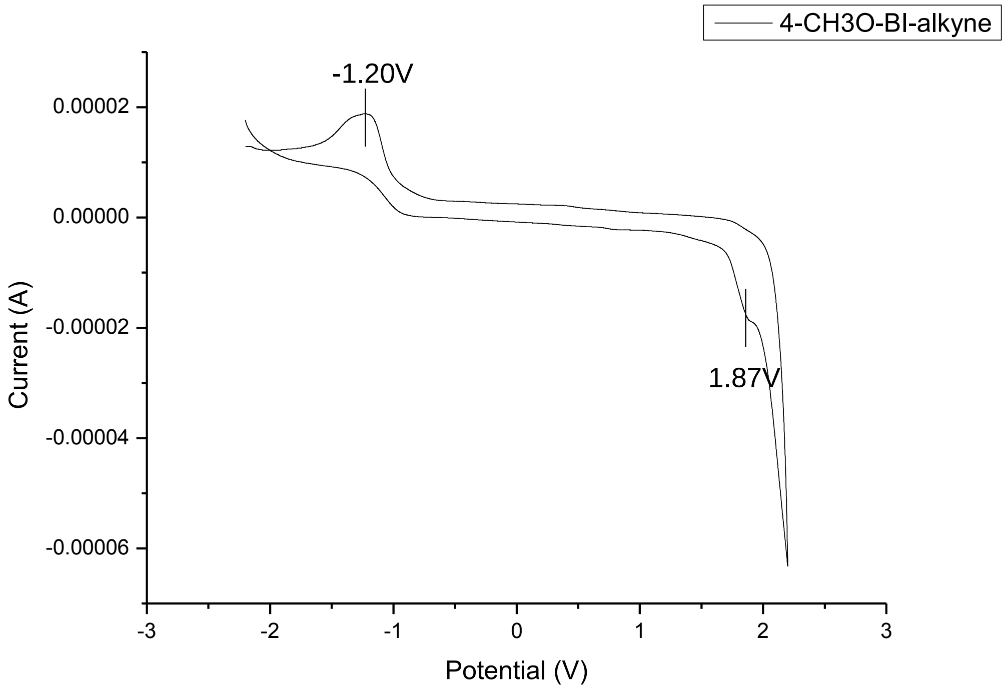

Scheme S6: The cyclic voltammetry experiment of 3e.

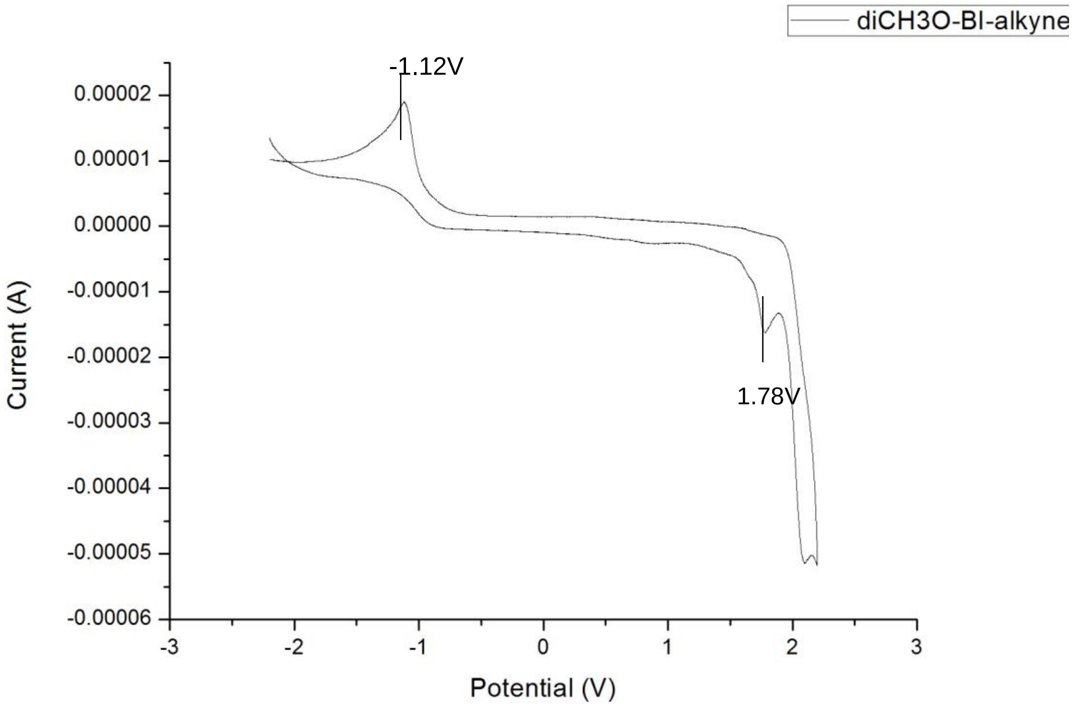

Scheme S7: The cyclic voltammetry experiment of 3f.

The solubility test of alkynylbenziodoxoles

The solution was prepared from 0.05 mmol of alkynylbenziodoxole and 0.5 ml of DCM. At this concentration, **3b** and **3d** can be completely dissolved and the solution is clear; **3c** and **3e** are in a slightly turbid state, while **3a** and **3f** are not completely dissolved and the solution is cloudy.

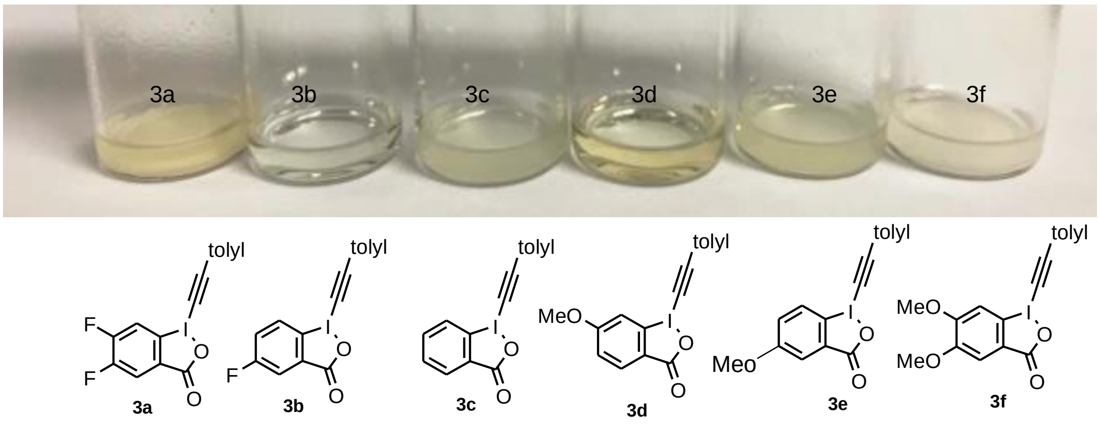

Scheme S8: The solubility of alkynylbenziodoxoles 3a–f.

III. Substrates preparation and characterization

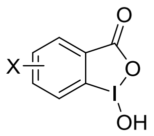

Synthesis of hydroxybenziodoxole 2. $NaIO_4$ (6.7 g, 31 mmol, 1.0 equiv) and 2-iodobenzoic acid (7.4 g, 30 mmol, 1.0 equiv) were suspended in 30% (v:v) aqueous acetic acid (45 mL). The reaction mixture was vigorously stirred and refluxed for 3 h protecting from light. Cold water (120 mL) was then added and allowed to cool to room temperature. After 1 h, the crude product was collected by filtration, washed with ice water (3 x 30 mL) and acetone (3 x 30 mL). After air-drying in the dark, hydroxybenziodoxole **2** was yielded as a white solid.

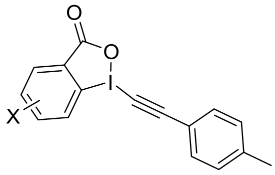

Synthesis of alkynylbenziodoxle 3[1]. Trimethylsilyl triflate (1.1 equiv) was added to a suspension of hydroxybenziodoxole (BI-OH, 1.0 equiv) in CH_2Cl_2 at room temperature. The resulting yellow mixture was stirred for 1 h, followed by the addition of trimethylsilyl alkyne (1.1 equiv). After stirring for 6 h at room temperature, saturated $NaHCO_3$ (100 mL) was added and the mixture was stirred vigorously for 30 min. After filtration, the filtrate was washed with saturated $NaHCO_3$, dried over Na_2SO_4, filtered and concentrated in vacuum. The resulting mixture was combined with the previously obtained solid and then recrystallized in CH_3CN to afford the alkynylbenziodoxole **3**.

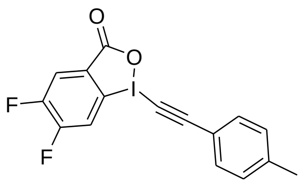

5,6-Difluoro-1-(*p*-tolylethynyl)-1λ^3-benzo[*d*][1,2]iodaoxol-3(1*H*)-one (3a). Following the general procedure, the reaction from 5,6-difluoro hydroxybenziodoxole (0.90 g, 3.0 mmol, 1.0 equiv) and trimethyl(*p*-tolylethynyl)silane (0.62 g, 3.3 mmol, 1.1equiv) afforded alkynylbenziodoxole **3a** as a pale yellow solid (305 mg, 25% yield): IR (neat) 1624, 1599, 1484, 1408, 1300, 1282, 813, 782, 655 cm^{-1}; ^{1}H NMR (500 MHz, $(CD_3)_2SO$) δ 8.11 (dd, *J* = 9.1, 6.7 Hz, 1H), 8.03 (dd, *J* = 9.7, 7.6 Hz, 1H), 7.60 (d, *J* = 8.1 Hz, 2H), 7.33 (d, *J* = 7.9 Hz, 2H), 2.38 (s, 3H); ^{13}C NMR (125 MHz, $(CD_3)_2SO$) δ 169.7 , 159.9 – 157.8 (m), 156.8 (dd, *J* = 240.1, 13.1 Hz), 146.3 , 137.8 , 135.2 (dd, *J* = 5.2, 2.9 Hz), 134.9 – 134.8 (m), 124.5 (dd, *J* = 31.7, 18.8 Hz), 122.3 , 122.2 (dd, *J* = 34.3, 22.8 Hz), 116.2 (d, *J* = 6.8 Hz), 110.7 , 56.6 , 26.4; ^{19}F NMR (375 MHz, $CDCl_3$) δ -125.8 (dt, *J* = 19.1, 8.0 Hz), -131.3 (ddd, *J* = 19.2, 9.3, 6.3 Hz); HRMS-ESI (m/z) $[M+H]^+$ calc'd for $C_{16}H_{10}O_2F_2I$, 398.9688, found 398.9700.

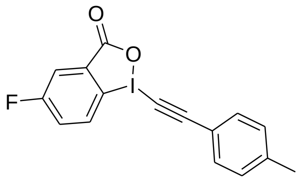

5-Fluoro-1-(*p*-tolylethynyl)-1λ^3-benzo[*d*][1,2]iodaoxol-3(1*H*)-one (3b). Following the general procedure, the reaction from 5-fluoro hydroxybenziodoxole (1.41 g, 5.0 mmol, 1.0 equiv) and trimethyl(p-tolylethynyl)silane (1.0 g, 5.5 mmol, 1.1 equiv) afforded alkynylbenziodoxole **3b** as a white solid (731 mg, 38% yield): IR (neat) 3069, 2921, 2154, 1628, 1578, 1508, 1453, 1415, 1257, 818, 784 cm^{-1}; ^{1}H NMR (500 MHz, $(CD_3)_2SO$) δ 8.25 (dd, J = 8.9, 4.5 Hz, 1H), 7.82 (dd, J = 8.3, 3.0 Hz, 1H), 7.77 (ddd, J = 8.9, 8.2, 3.0 Hz, 1H), 7.60-7.56 (m, 2H), 7.33-7.28 (m, 2H), 2.37 (s, 3H); ^{13}C NMR (125 MHz, $(CD_3)_2SO$ δ 165.4 (d, J = 2.0 Hz), 164.8 (d, J = 249.3 Hz), 141.4, 135.4 (d, J = 7.1 Hz), 133.0, 130.1 (d, J = 8.6 Hz), 130.1, 122.7 (d, J = 24.0

Hz), 118.1 (d, J = 23.6 Hz), 117.8, 110.4 (d, J = 1.9 Hz), 105.5, 51.0, 21.6; ^{19}F NMR (375MHz, $(CD_3)_2SO$) δ -111.6 (tt, J = 8.1, 3.5 Hz), HRMS-ESI (m/z) $[M+H]^+$ calc'd for $C_{16}H_{11}O_2FI$, 380.9782, found 380.9784.

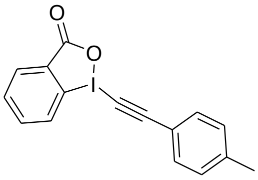

1-(p-Tolylethynyl)-1λ^3-benzo[*d*][1,2]iodaoxol-3(1*H*)-one (3c) (CAS: 1433188-23-8). Following the general procedure, the reaction from hydroxybenziodoxole (1.3 g, 5.0 mmol, 1.0 equiv) and trimethyl(p-tolylethynyl)silane (0.94 g, 5.0 mmol, 1.0 equiv) afforded alkynylbenziodoxole **3c** as a white solid (1.1 g, 59% yield): ^{1}H NMR (500 MHz, $CDCl_3$) δ 8.39 (dd, *J* = 6.5, 1.3 Hz, 1H), 8.23 (d, *J* = 7.7 Hz, 1H), 7.79–7.69 (m, 2H), 7.48 (d, *J* = 7.6 Hz,2H), 7.22 (d, *J* = 7.6 Hz, 2H), 2.41 (s, 3H); ^{13}C NMR (125 MHz, $CDCl_3$) δ 166.8, 141.6, 134.9, 132.9, 132.5, 131.6, 131.5, 129.6, 126.4, 117.5, 116.4, 107.2, 49.3, 21.8.

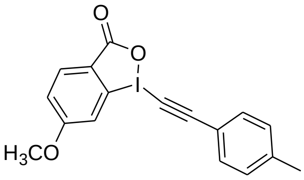

6-Methoxy-1-(*p*-tolylethynyl)-1λ^3-benzo[*d*][1,2]iodaoxol-3(1*H*)-one (3d). Following the general procedure, the reaction from 6-methoxy-hydroxybenziodoxole (1.5 g, 5.0 mmol, 1.0 equiv) and trimethyl(p-tolylethynyl)silane (1.2 g, 5.5 mmol, 1.1 equiv) afforded alkynylbenziodoxole **3d** as a pale yellow solid (946mg, 48% yield): IR (neat) 2146, 1597, 1556, 1482, 1305， 1265, 1233， 1205, 814, 798, 630 cm^{-1}; ^{1}H NMR (500 MHz, $(CD_3)_2SO$) δ 7.98 (d, *J* = 8.4 Hz, 1H), 7.71 (d, *J* = 2.3 Hz, 1H), 7.59-7.54 (m, 2H), 7.31 (m, 3H), 3.86 (s, 3H), 2.36 (s, 3H); ^{13}C NMR (125 MHz, $(CD_3)_2SO$) δ 166.7, 164.8, 141.3, 132.8, 132.5, 130.1, 125.1, 118.7, 117.8, 117.5,

112.6, 105.2, 56.4, 52.9, 21.6; HRMS-ESI (m/z) $[M+H]^+$ calc'd for $C_{17}H_{14}O_3I$, 392.9982, found 392.9983.

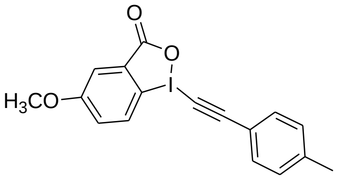

5-Methoxy-1-(*p*-tolylethynyl)-1λ^3-benzo[*d*][1,2]iodaoxol-3(1*H*)-one (3e). Following the general procedure, the reaction from 5-methoxy-hydroxybenziodoxole (1.5 g, 5.0 mmol, 1.0 equiv) and trimethyl(p-tolylethynyl)silane (1.2 g, 5.5 mmol, 1.1 equiv) afforded alkynylbenziodoxole **3e** as a white solid (1.2 g, 48% yield): IR (neat) 3481, 2148, 1723, 1617, 1574, 1507, 1462, 1409, 1315, 1272, 813, 787 cm^{-1}; ^1H NMR (500 MHz, $(CD_3)_2SO$) δ 8.09 (d, J = 9.0 Hz, 1H), 7.61 (d, J = 3.0 Hz, 1H), 7.57 (d, J = 8.1 Hz, 2H), 7.47 (dd, J = 9.0, 3.0 Hz, 1H), 7.30 (d, J = 8.4 Hz, 2H), 3.86 (s, 3H), 2.37 (s, 3H); ^{13}C NMR (125 MHz, $(CD_3)_2SO$) δ 166.3, 162.6, 141.3, 134.2, 132.9, 130.0, 128.7, 122.3, 117.9, 115.8, 105.0, 104.9, 56.4, 51.2, 21.6; HRMS-ESI (m/z) $[M+H]^+$ calc'd for $C_{17}H_{14}O_3I$, 392.9982, found 392.9988.

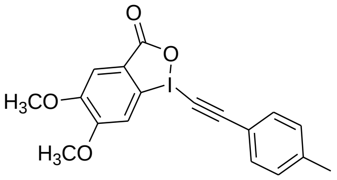

5,6-Dimethoxy-1-(*p*-tolylethynyl)-1λ^3-benzo[*d*][1,2]iodaoxol-3(1*H*)-one (3f) (CAS: 2074732-35-5). Following the general procedure, the reaction from 5,6-dimethoxy-hydroxybenziodoxole (1.6 g, 5.0 mmol, 1.0 equiv) and trimethyl(*p*-tolylethynyl)silane (1.2 g, 5.5 mmol, 1.1 equiv) afforded alkynylbenziodoxole **3f** as a white solid (1.4 g, 65% yield): ^1H NMR (500 MHz, $CDCl_3$) δ 7.82 (s, 1H), 7.64 (s, 1H), 7.48-7.43 (m, 2H), 7.22 (d, *J* = 7.6 Hz, 2H), 3.98 (s, 3H), 3.92 (s, 3H), 2.40 (s, 3H); ^{13}C NMR (125 MHz, $(CD_3)_2SO$) δ 166.8, 154.5,

151.7, 141.2, 132.7, 130.1, 125.4, 117.9, 113.0, 109.3, 106.2, 104.9, 56.5, 56.2, 52.5, 21.6.

General synthesis of alkyl trifluoroborates [1]. To the solution of alkyl boronic acid or pinacol ester (10 mmol) in methanol (20 mL) was added saturated aqueous KHF_2 (15 mL, 3.91 g, 50 mmol, 5.0 equiv). The resulting suspension was stirred for 2 h and then concentrated to dryness. The residue, a white solid, was extracted with hot acetone, and the combined filtered extracts were concentrated to approximately 5 mL. Ether (or CH_2Cl_2) was added and the resultant precipitate was collected and dried to afford the potassium trifluoroborate as a white solid.

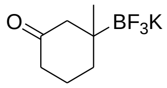

Potassium (1-methyl-3-oxocyclohexyl)trifluoroborates (4a) (CAS: 1260112-11-5). Following the literature procedure [1], bis(pinacolato)diboron (5.6 g, 22 mmol, 2.2 equiv), 3-methylcyclohex-2-enone (2.2 g, 10.0 mmol, 1.0 equiv), and 4-picoline (0.10 g, 1.0 mmol, 0.10 equiv) were added to a 100 mL round-bottomed flask with a magnetic stir bar. A solution of $CuSO_4{\cdot}5H_2O$ (50 mg, 0.2 mmol) in distilled water (15 mL) was added to the reaction vessel. The reaction mixture was stirred vigorously for 3 h at room temperature until completion indicated by TLC. EtOAc (50 mL) was then added and allowed to stir for 15 minutes. The aqueous layer was extracted with EtOAc (2 × 30 mL) and the combined organic extracts were washed with brine (50 mL), dried over sodium sulfate, filtered, concentrated and purified by column chromatography to afford the alkyl boronic acid. The alkyl boronic acids afforded trifluoroborate **4a** via the general procedure as a white solid (3.3 g, 76% yield): ^{1}H NMR (500 MHz, $(CD_3)_2SO$) δ 2.18 (dd, J = 13.3, 1.4 Hz, 1H), 2.14-2.06 (m, 1H), 2.00-1.93 (m, 1H), 1.84 (tdd, J = 9.1, 5.7, 2.4 Hz, 1H), 1.75-1.61 (m, 2H), 1.60-1.52 (m, 1H), 1.13 (dd, J = 13.4, 6.1 Hz, 1H), 0.60 (d, J = 2.1 Hz, 3H);

^{13}C NMR (125 MHz, $(CD_3)_2SO$) δ 215.1, 51.3, 41.8, 33.6, 24.0, 22.9; ^{19}F NMR (375 MHz, $(CD_3)_2SO$) δ -144.9(d, J = 78.6 Hz).

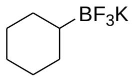

Potassium cyclohexyltrifluoroborate (4b) (CAS: 446065-11-8). Following the general procedure, the reaction from cyclohexylboronic acid (1.3 g, 10.0 mmol, 1.0 equiv) afforded potassium cyclohexyltrifluoroborate as a white solid (1.6 g, 79% yield): ^{1}H NMR (500 MHz, $(CD_3)_2SO$) δ 1.61-1.52 (m, 3H), 1.52-1.44 (m, 2H), 1.14-0.98 (m, 3H), 0.94-0.82 (m, 2H), -0.02 (d, J = 23.2 Hz, 1H); ^{13}C NMR (125 MHz, $(CD_3)_2SO$) δ 29.3, 28.7, 27.9; ^{19}F NMR (375 MHz, $(CD_3)_2SO$) δ -144.4 (d, J = 96.1 Hz).

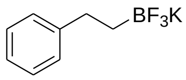

Trifluoro(phenethyl)-λ^4-borane, potassium salt 4c (CAS: 329976-74-1). Following the literature procedure [1], styrene (1.0 g, 10.0 mmol, 1.0 equiv) in THF (2.0 mL) was added dropwise to a solution of BH_3·THF (20.0 mL, 20.0 mmol, 1.0 M solution in THF, 2.0 equiv) at 0 °C. The mixture was stirred for 2 h at room temperature and H_2O (2.0 mL) was slowly added. After stirring for additional 3 h at room temperature, the reaction mixture was concentrated in vacuo, diluted with ethyl acetate (30 mL), and washed with saturated aqueous bicarbonate (20 mL) and brine (20 mL). The organic layer was dried over sodium sulfate, filtered, and concentrated to approximately 5 mL. Petroleum ether was then added. The resultant precipitate was washed with petroleum ether and dried under vacuum to afford the alkylboronic acid as a white solid. The alkyl boronic acids afford trifluoroborate **4c** via the general procedure as a white solid **(**86.3 mg, 41% yield): ^{1}H NMR (500 MHz, $(CD_3)_2SO$) δ 7.19 (t, J = 7.6 Hz, 2H), 7.14-7.10 (m, 2H), 7.08-7.01 (m, 1H), 2.46-2.39 (m, 2H), 0.29 (dp, J = 16.8, 6.2 Hz, 2H); ^{13}C NMR (125 MHz, $(CD_3)_2SO$) δ 148.5 , 128.3 , 128.1 , 124.8 , 32.6 (q, J = 2.5 Hz); ^{19}F NMR (375 MHz, $CDCl_3$) δ -137.3.

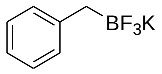

Benzyltrifluoro-λ^4-borane, potassium salt 4d (CAS: 329976-73-0). Following the literature procedure [1], CuI (19 mg,1.0 mmol, 0.10 equiv), PPh_3 (367 mg, 1.4 mmol, 0.14 equiv), LiOMe (760 mg, 20.0 mmol, 2.0 equiv), and bis(pinacolato)diboron (3.80 g, 15 mmol, 1.5 equiv) were added to a 250 mL round-bottomed flask equipped with a stir bar. The vessel was evacuated and filled with nitrogen gas three times. DMF (40 mL) and the benzyl bromide (1.7 g, 10.0 mmol, 1.0 equiv) were added by syringe under a nitrogen atmosphere. The resulting reaction mixture was stirred vigorously at 25 °C for 18 h. The reaction mixture was then diluted with EtOAc and filtered through silica gel with copious EtOAc. The filtrate was concentrated and purified by column chromatography to afford the pinacol ester. The pinacol ester afforded trifluoroborate **4d** via the general procedure as a white solid (983 mg, 50% yield): ^{1}H NMR (500 MHz, $(CD_3)_2SO$) δ 7.03 (dd, *J* = 8.0, 7.1 Hz, 2H), 7.00-6.95 (m, 2H), 6.86 (tt, *J* = 7.0, 1.5 Hz, 1H), 1.46 (q, *J* = 6.3 Hz, 2H); ^{13}C NMR (125 MHz, $(CD_3)_2SO$) δ 147.3, 129.0, 127.3, 122.4; ^{19}F NMR (375 MHz, $(CD_3)_2CO$) δ 140.7 (dd, *J* = 112.7, 50.6 Hz).

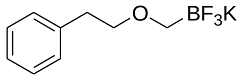

Trifluoro(phenethoxymethyl)-λ^4-borane, potassium salt 4e. Following the literature procedure [2], NaH (360 mg, 15 mmol, 3.0 equiv) was added to a 50 mL 2-neck round-bottom-flask and purged with N_2 three times. The content was diluted with dry THF (40 mL) and phenylethyl alcohol (1.8 g, 15.0 mmol, 3.0 equiv) was then added dropwise to the reaction mixture at 0 °C under N_2. After stirring for 15 min at 0 ^{o}C, the temperature was increased to rt and further stirred for 30 min. Bromomethyltrifluoroborate (1.0 g, 5 mmol, 1.0 equiv) was added in one portion to the suspension at 0 °C, and the reaction was stirred at rt for 3 h. The reaction was quenched by adding saturated solution of KHF_2 (20 mL). The final mixture was stirred for 30 min and then concentrated and dried overnight under high vacuum to

remove the trace solvent. The crude residue was suspended in anhydrous acetone (3 × 50 mL) and filtered. The filtrate was concentrated to a minimal volume (5–10 mL) and Et_2O (≈150 mL) was added to precipitate. The white precipitate was isolated by filtration, washing with hexanes (≈30 mL) and CH_2Cl_2 (≈30 mL), to afforded trifluoborate **4e** as a white solid (875 mg, 72% yield): IR (neat) 2950, 2882, 2862, 1454, 1244, 1084, 1006, 958, 806, 699 cm^{-1}; ^1H NMR (500 MHz, $(CD_3)_2SO$) δ 7.27-7.23 (m, 2H), 7.22-7.18 (m, 2H), 7.18-7.14 (m, 1H), 3.38 (t, *J* = 7.5 Hz, 2H), 2.74 (t, *J* = 7.4 Hz, 2H), 2.53 (q, *J* = 5.5 Hz, 2H); ^{13}C NMR (125 MHz, $(CD_3)_2SO$) δ 140.1, 129.2, 128.6, 126.2, 74.9, 36.4; ^{19}F NMR (375 MHz, $CDCl_3$) δ -140.7. HRMS-ESI (m/z) $[M]^-$ calc'd for $C_9H_{11}O_{10}BF_{3,}$202.0897, found 202.0898.

General synthesis of tertiary alcohols [3]. A flame-dried round bottom flask charged with 40 mmol (4.0 equiv) of 4-bromoanisole in 40 mL THF was cooled to −78 °C and 16 mL BuLi (2.5 M in hexane, 40 mmol, 4.0 equiv) was added dropwise via syringe. The mixture was stirred for 1.5 h at −78 °C before the addition of the corresponding methyl ester (10 mmol, 1.0 equiv), then the mixture was warmed to room temperature and stirred overnight. After ammonium chloride solution quenching, the precipitated solid was removed by filtration. The filtrate was extracted with ethyl acetate (3 × 30 mL), washed with sodium chloride solution, and dried over Na_2SO_4. After filtration and concentration, the residue was purified by column chromatography to afford the tertiary alcohol as a white solid.

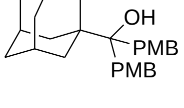

2-((3*r*,5*r*,7*r*)-Adamantan-1-yl)-1,3-bis(4-methoxyphenyl)propan-2-ol (6a) (CAS: 210624-71-8), Following the general synthesis of tertiary alcohols [3], the reaction adamantane-1-carboxylic acid methyl ester (1.9 g, 10 mmol, 1.0 equiv) afforded tertiary alcohol **6a** as a white solid (1.1 g, 27% yield): TLC R_f = 0.29 (EA/PE = 1:10); ^1H NMR (500 MHz, $CDCl_3$) δ 7.47(d, *J* = 9.0 Hz, 4H), 6.80 (d, *J* = 9.0 Hz, 4H), 3.79

(s, 6H), 2.16 (s, 1H), 1.99 (s, 3H), 1.84 (d, *J* = 2.5 Hz, 6H), 1.63 (q, *J* = 11.9 Hz, 6H); ^{13}C NMR (125MHz, $CDCl_3$) δ 157.8, 137.8, 129.7, 112.4, 81.8, 55.1, 41.2, 37.4, 37.0, 28.8.

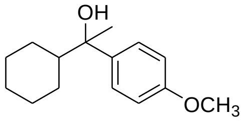

1-Cyclohexyl-1-(4-methoxyphenyl)ethan-1-ol (6b) (CAS: 97704-00-2)**.** Following the general synthesis of tertiary alcohols, the reaction from 4-bromoanisol (2.8 g, 15mmol, 1.5 equiv), *n*-BuLi (2.5 M in hexane, 15 mmol, 1.5 equiv) and 1-cyclohexylethan-1-one (1.26 g, 10 mmol, 1.0 equiv) afforded tertiary alcohol **6b** as a colorless oil (1.96 g, 80% yield): TLC R_f = 0.59 (EA/PE = 1:6); ^{1}H NMR (500 MHz, $CDCl_3$) δ 7.32 (d, *J* = 8.9 Hz, 2H), 6.87 (d, *J* = 8.9 Hz, 2H), 3.81 (s, 3H), 1.73 (m, 3H), 1.66 – 1.61 (m, 2H), 1.59 – 1.54 (m, 1H), 1.52 (s, 3H), 1.18 (dddq, *J* = 15.9, 9.8, 6.4, 3.2 Hz, 2H), 1.08 (tt, *J* = 13.0, 3.3 Hz, 1H), 0.96 (dtt, *J* = 16.4, 8.6, 3.7 Hz, 2H); ^{13}C NMR (125MHz, $CDCl_3$) δ 158.1, 140.0, 126.5, 113.1, 76.4, 55.2, 49.2, 27.4, 27.3, 26.7 (d, *J* = 3.1 Hz). 26.4**.**

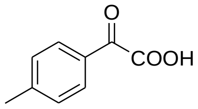

Synthesis of ketoacid 2-oxo-2-(*p*-tolyl)acetic acid (8) (CAS: 7163-50-0). Following the slightly modified literature procedure [5], in a round-bottomed flask mounted with a cooling system under N_2, $AlCl_3$ **(**2.6 g, 20 mmol, 2.0 equiv**)** was suspended in CH_2Cl_2 **(**15 mL**)** at 0 °C. To this mixture mono-ethyloxalyl chloride **(**2.7 g, 20 mmol, 2.0 equiv**)** was added dropwise in about 15 min. After 10 min the stirred suspension became a pale yellow solution. At 0 °C, toluene (0.92 g, 40 mmol, 4.0 equiv**)** was added dropwise in about 10 min. The solution was then stirred at rt for 1 h before was poured over crushed ice and 50 mL of concentrated hydrochloric acid. Extraction was performed with CH_2Cl_2, the organic layer was collected and washed

with 0.1 N sodium hydroxide (40 mL) and brine. After the organic layer was separated and dried over Na_2SO_4, the solvent was evaporated and the crude product directly subjected to hydrolysis to afford **8** as a white needle crystal: ^{1}H NMR (500 MHz, $CDCl_3$) δ 8.55 (s, 1H), 8.26-8.19 (m, 2H), 7.33 (d, *J* = 8.0 Hz, 2H), 2.45 (s, 3H); ^{13}C NMR (125 MHz, $CDCl_3$) δ 183.9, 162.3, 147.3, 131.4, 129.7, 129.2, 22.0.

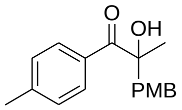

2-Hydroxy-2-(4-methoxyphenyl)-1-(*p*-tolyl)propan-1-one (10) (CAS: 2074732-28-6). Following the literature procedure [6], a flame-dried round bottom flask charged with p-bromotoluene (2.6 g, 15 mmol, 1.5 equiv) in 20 mL THF was cooled to −78 °C and 6 mL *n*-BuLi (2.5 M in hexane, 15 mmol, 1.5 equiv) was added dropwise via syringe under a nitrogen atmosphere. The mixture was stirred for 1.5 h at −78 °C before the addition of 2-(4-methoxyphenyl)-2-((trimethylsilyl)oxy)-propanenitrile (2.5 g , 10 mmol, 1.0 equiv), then the mixture was warmed to room temperature and stirred over 3 h. After ammonium chloride solution quenching, the precipitated solid was removed by filtration. The filtrate was extracted with ethyl acetate, washed with sodium chloride solution, and dried over Na_2SO_4. After filtration and concentration, the crude product and 40 ml THF was added to a 100 mL round-bottomed flask equipped with a stir bar. 10 ml 10% HCl was added via syringe at 0 °C and stirred for 5 h at the same temperature. After 30 ml H_2O quenching, the solution was extracted with ethyl acetate, washed with sodium chloride solution, and dried over Na_2SO_4. After filtration and concentration, the residue was purified by column chromatography to afford **10** as a pale yellow oil: TLC R_f = 0.38 (EA/PE =1:5); ^{1}H NMR (500 MHz, $CDCl_3$) δ 7.61 (d, *J* = 8.3 Hz, 2H), 7.36 (d, *J* = 8.9 Hz, 2H), 7.11 (d, *J* = 9.1 Hz, 2H), 6.91 (d, *J* = 8.9 Hz, 2H), 4.89 (s, 1H), 3.81 (s, 3H), 2.34 (s, 3H), 1.89 (s, 3H); ^{13}C NMR (125 MHz, $CDCl_3$) δ 201.7, 159.3, 143.9, 134.8, 130.6, 130.4, 129.0, 127.3, 114.3, 78.3, 55.3, 26.0, 21.6.

IV. Radical alkynylation product characterization

Standard procedure for alkyl trifluoroborates. Following the literature procedure [1], alkyl potassium trifluoroborate (0.15 mmol, 1.5 equiv), alkynylbenziodoxole (0.10 mmol, 1.0 equiv), $Ru(bpy)_3(PF_6)_2$ (1.7 mg, 0.002 mmol, 0.02 equiv), hydroxybenziodoxole (BI-OH, 13.2 mg, 0.05 mmol, 0.5 equiv), and Na_2CO_3 (21.2 mg, 0.2 mmol, 2.0 equiv) were placed in a 5 mL clear-colored glass vial equipped with a magnetic stir bar. After 1.0 mL CH_2Cl_2 and 1.0 mL H_2O (water was bubbled with nitrogen gas for 30 minutes to remove oxygen before the experiment) were added, the vial was sealed and exposed to blue LEDs at room temperature for 20 h with stirring until TLC indicated the complete consumption of alkynylbenziodoxoles. The reaction mixture was extracted with CH_2Cl_2 and the organic layer was concentrated and purified directly by column chromatography to afford the product.

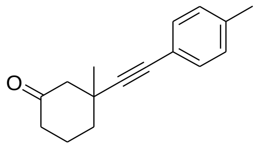

3-Methyl-3-(*p*-tolylethynyl)cyclohexan-1-one (5a). Following the standard procedure for alkyl trifluoroborates, the reaction of trifluoroborate **4a** (65.4 mg, 0.3 mmol, 3.0 equiv), alkynylbenziodoxole **3a** (39.8 mg, 0.1 mmol, 1.0 equiv), and Na_2CO_3 (42.4 mg, 0.4 mmol, 4.0 equiv) for 20 h afforded alkyne **5a** as a colorless oil (10.6 mg, 47% yield) after flash chromatography. When alkynylbenziodoxole **3b** (38.0 mg, 0.10 mmol, 1.0 equiv) was used, 8.8 mg, 39% yield of alkyne **5a** was obtained. When alkynylbenziodoxole **3c** (36.2 mg, 0.10 mmol, 1.0 equiv) was used, 11.3 mg, 50% yield of alkyne **5a** was obtained. When alkynylbenziodoxole **3d** (39.2 mg, 0.10 mmol, 1.0 equiv) was used, 11.6 mg, 52% yield of alkyne **5a** was obtained. When alkynylbenziodoxole **3e** (39.2 mg, 0.10 mmol) was used, 10.4 mg, 47% yield of alkyne **5a** was obtained when alkynylbenziodoxole **3f** (42.2 mg, 0.10 mmol) was used, 16.8 mg, 74% yield of alkyne **5a** was obtained: TLC R_f = 0.45 (EtOAc / PE = 1/10); IR (neat) 2963, 2928, 2870, 1715, 1510, 1455, 1310, 1228, 817 cm^{-1}; ^1H NMR

(500 MHz, $CDCl_3$) δ 7.26-7.23 (m, 2H), 7.09-7.05 (m, 2H), 2.59 (dt, J = 13.7, 2.0 Hz, 1H), 2.45-2.37 (m, 1H), 2.34-2.30 (m, 4H), 2.28-2.11 (m, 2H), 2.06-1.95 (m, 2H), 1.74-1.67 (m, 1H), 1.41 (s, 3H); ^{13}C NMR (125 MHz, $CDCl_3$) δ 209.3, 137.9, 131.5, 128.9, 120.1, 92.4, 83.6, 54.2, 40.6, 37.9, 36.7, 29.3, 22.9, 21.4; HRMS-ESI (m/z) $[M+H]^+$ calc'd for $C_{16}H_{18}O$, 227.1430, found 227.1428.

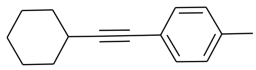

1-(Cyclohexylethynyl)-4-methylbenzene (5b) (CAS: 1529771-44-5). Following the standard procedure for alkyl trifluoroborates, the reaction of trifluoborate **4b** (28.5 mg, 0.15 mmol, 1.5 equiv) and alkynylbenziodoxole **3a** (39.8 mg, 0.10 mmol, 1.0 equiv) for 20 h afforded alkyne **5b** as a colorless oil (15.8 mg, 80% yield) after flash chromatography. When alkynylbenziodoxole **3b** (38.0 mg, 0.10 mmol, 1.0 equiv) was used, 16.4 mg, 83% yield of alkyne **5b** was obtained When alkynylbenziodoxole **3c** (36.2 mg, 0.10 mmol, 1.0 equiv) was used, 16.4 mg, 83% yield of alkyne **5b** was obtained**.** When alkynylbenziodoxole **3d** (39.2 mg, 0.10 mmol, 1.0 equiv) was used, 14.7 mg, 74% yield of alkyne **5b** was obtained**.** When alkynylbenziodoxole **3e** (39.2 mg, 0.10 mmol, 1.0 equiv) was used, 16.6 mg, 84% yield of alkyne **5b** was obtained**.** When alkynylbenziodoxole **3f** (42.2 mg, 0.10 mmol, 1.0 equiv) was used, 17.0 mg, 86% yield of alkyne **5b** was obtained. TLC R_f = 0.6(PE); ^1H NMR (500 MHz, $CDCl_3$) δ 7.29 (d, J = 8.1 Hz, 2H), 7.08 (d, J = 7.8 Hz, 2H), 2.57 (tt, J = 8.9, 3.6 Hz, 1H), 2.33 (s, 3H), 1.92-1.84 (m, 2H), 1.75 (tt, J = 8.3, 4.1 Hz, 2H), 1.58-1.48 (m, 3H), 1.40-1.30 (m, 3H); ^{13}C NMR (125 MHz, $CDCl_3$) δ 137.3, 131.4, 128.9, 121.0, 93.6, 80.5, 32.8, 29.7, 25.9, 24.9, 21.4.

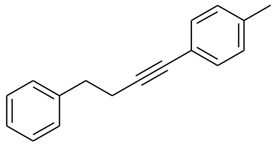

1-Methyl-4-(4-phenylbut-1-yn-1-yl)benzene (5c) (CAS: 1064664-64-7**).** Following the standard procedure for alkyl trifluoroborates, the reaction of

trifluoborate **4c** (31.8 mg, 0.15 mmol, 1.5 equiv) and alkynylbenziodoxole **3a** (39.8 mg, 0.10 mmol, 1.0 equiv) for 20 h afforded alkyne **5c** as a colorless oil (14.4 mg, 65% yield) after flash chromatography. When alkynylbenziodoxole **3c** (36.2 mg, 0.10 mmol, 1.0 equiv) was used, 14.9 mg, 68% yield of alkyne **5c** was obtained. When alkynylbenziodoxole **3f** (42.2 mg, 0.10 mmol, 1.0 equiv) was used, 16.1 mg, 73% yield of alkyne **5c** was obtained: TLC R_f = 0.4 (PE); ^{1}H NMR (500 MHz, $CDCl_3$) δ 7.35-7.31 (m, 2H), 7.30-7.26 (m, 4H), 7.26-7.22 (m, 1H), 7.12-7.08 (m, 2H), 2.94 (t, *J* = 7.6 Hz, 2H), 2.70 (t, *J* = 7.6 Hz, 2H), 2.34 (s, 3H); ^{13}C NMR (125 MHz, $CDCl_3$) δ 140.8, 137.6, 131.4, 128.9, 128.5, 128.4, 126.3, 120.8, 88.7, 81.3, 35.3, 21.7, 21.4.

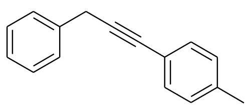

1-Methyl-4-(3-phenylprop-1-yn-1-yl)benzene (5d) (CAS: 852031-46-0). Following the standard procedure for alkyl trifluoroborates, the reaction of trifluoborate **4d** (29.7 mg, 0.15 mmol, 1.5 equiv) and alkynylbenziodoxole **3a** (39.8 mg, 0.10 mmol, 1.0 equiv) for 20 h afforded alkyne **5d** as a colorless oil (12.8 mg, 62% yield) after flash chromatography. When alkynylbenziodoxole **3c** (36.2 mg, 0.10 mmol, 1.0 equiv) was used, 12.4 mg, 60% yield of alkyne **5d** was obtained. When alkynylbenziodoxole **3f** (42.2 mg, 0.10 mmol, 1.0 equiv) was used, 14.4 mg, 70% yield of alkyne **5d** was obtained. TLC R_f = 0.54 (PE); ^{1}H NMR (400 MHz, $CDCl_3$) δ 7.45 (d, *J* = 7.5 Hz, 2H), 7.40-7.34 (m, 4H), 7.29 (d, *J* = 6.7 Hz, 1H), 7.14 (d, *J* = 7.9 Hz, 2H), 3.86 (s, 2H), 2.37 (s, 3H); ^{13}C NMR (125 MHz, $CDCl_3$) δ 137.8, 136.9, 131.5, 129.0, 128.5, 127.9, 126.6, 120.6, 86.7, 82.7, 25.8, 21.4.

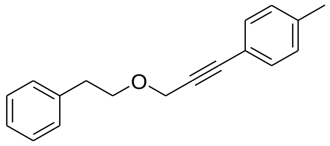

1-Methyl-4-(3-phenethoxyprop-1-yn-1-yl)benzene (5e). Following the standard procedure for alkyl trifluoroborates, the reaction of trifluoborate **4e** (36.3 mg, 0.15

mmol, 1.5 equiv) and alkynylbenziodoxole **3a** (39.8 mg, 0.10 mmol, 1.0 equiv) for 20 h afforded alkyne **5c** as a colorless oil (20.2 mg, 81% yield) after flash chromatography. When alkynylbenziodoxole **3c** (36.2 mg, 0.10 mmol, 1.0 equiv) was used, 18.8 mg, 75% yield of alkyne **5e** was obtained**.** When alkynylbenziodoxole **3f** (42.2 mg, 0.10 mmol, 1.0 equiv) was used, 20.5 mg, 82% yield of alkyne **5e** was obtained: TLC R_f = 0.41 (EtOAc/PE = 1/20); IR (neat) 3027, 2962, 2854, 1604, 1509, 1261, 1095, 1022, 816, 800 cm^{-1}; ^{1}H NMR (500 MHz, $CDCl_3$) δ 7.36-7.33 (m, 2H), 7.33-7.28 (m, 2H), 7.28-7.25 (m, 2H), 7.25-7.20 (m, 1H), 7.12 (dt, *J* = 7.7, 0.8 Hz, 2H), 4.39 (s, 2H), 3.83 (t, *J* = 7.2 Hz, 2H), 2.97 (t, *J* = 7.2 Hz, 2H), 2.35 (s, 3H); ^{13}C NMR (125 MHz, $CDCl_3$) δ 138.7, 138.5, 131.7, 129.0, 128.9, 128.4, 126.3, 119.6, 86.3, 84.4, 70.9, 59.0, 36.1, 21.5; HRMS-ESI (m/z) $[M+NH_4]^+$ calc'd for $C_{18}H_{22}ON$, 268.1696, found 268.1695.

Standard procedure for tertiary alcohols. Following the literature procedure [3], tertiary alcohol (0.25 mmol, 2.5 equiv), alkynylbenziodoxole (0.10 mmol, 1.0 equiv), $Ru(bpy)_3(PF_6)_2$ (1.7 mg, 0.002 mmol, 0.02 equiv), and BI-OAc (76.5 mg, 0.25 mmol, 2.5 equiv) were placed in a 5 mL clear-colored glass vial equipped with a magnetic stir bar. After 2.0 mL DCE (anhydrous) were added, the vial was sealed and exposed to blue LEDs at room temperature for 24 h with stirring. After TLC indicated the complete consumption of alkynylbenziodoxoles, the reaction mixture was concentrated and purified directly by column chromatography to afford the product.

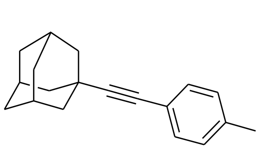

(3*r*,5*r*,7*r*)-1-(*p*-Tolylethynyl)adamantine (7a) (CAS: 851073-59-1)**.** Following the standard procedure for tertiary alcohols, the reaction of **6a** (94.6 mg, 0.25 mmol, 2.5 equiv) and alkynylbenziodoxole **3a** (40.2 mg, 0.10 mmol, 1.0 equiv) for 24 h afforded **7a** as a white solid (17.0 mg, 67% yield) after flash chromatography. When

alkynylbenziodoxole **3c** (36.2 mg, 0.10 mmol, 1.0 equiv) was used, 18.4 mg, 74% yield of alkyne **7a** was obtained**.** When alkynylbenziodoxole **3f** (42.2 mg, 0.10 mmol, 1.0 equiv) was used, 21.2 mg, 85% yield of alkyne **7a** was obtained. TLC R_f = 0.69 (PE); ^{1}H NMR (500 MHz, $CDCl_3$) δ 7.28 (d, *J* = 8.1 Hz, 2H), 7.07 (d, *J* = 7.9 Hz, 2H), 2.33 (s, 3H), 2.03-1.94 (m, 9H), 1.73 (t, *J* = 2.9 Hz, 6H); ^{13}C NMR (125 MHz, $CDCl_3$) δ 137.2, 131.5, 128.8, 121.0, 97.6, 79.3, 42.9, 36.4, 30.0, 28.1, 21.4.

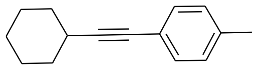

1-(Cyclohexylethynyl)-4-methylbenzene (5b) (CAS: 1529771-44-5). Following the standard procedure for tertiary alcohols, the reaction of **6b** (58.6 mg, 0.25 mmol, 2.5 equiv) and alkynylbenziodoxole **3a** (39.9 mg, 0.10 mmol, 1.0 equiv) for 24 h afforded **5b** as a colorless oil (14.8 mg, 75% yield) after flash chromatography; When alkynylbenziodoxole **3c** (36.5 mg, 0.10 mmol, 1.0 equiv) was used, 14.6 mg, 74% yield of alkyne **5b** was obtained**.** When alkynylbenziodoxole **3f** (42.4 mg, 0.10 mmol, 1.0 equiv) was used, 15.8 mg, 80% yield of alkyne **5b** was obtained.

Standard procedure for ketoacids. Following the literature procedure [5], alketoacid (0.15 mmol, 1.5 equiv), alkynylbenziodoxole (0.10 mmol, 1.0 equiv), $Ru(bpy)_3(PF_6)_2$ (1.7 mg, 0.002 mmol, 0.02 equiv), and 1-acetoxy-1,2-benziodoxol-3-(1H)-one (BIOAc, 30.6 mg, 0.10 mmol, 1.0 equiv) were placed in a 5 mL clear-colored glass vial equipped with a magnetic stir bar. After 2.0 mL CH_2Cl_2 were added, the vial was sealed and exposed to blue LEDs at room temperature for 5 h with stirring until TLC indicated the complete consumption of alkynylbenziodoxoles. The reaction mixture was concentrated and purified directly by column chromatography to afford the product.

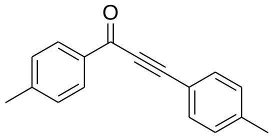

1,3-Di-p-tolylprop-2-yn-1-one 9 (CAS: 97691-66-2). Following the standard procedure for ketoacids, the reaction of ketoacid **8** (24.6 mg, 0.15 mmol, 1.5 equiv) and alkynylbenziodoxole **3a** (40.6 mg, 0.10 mmol, 1.0 equiv) for 5 h afforded ynone **9** as a white solid (15.1 mg, 63% yield) after flash chromatography. When alkynylbenziodoxole **3c** (37.2 mg, 0.10 mmol, 1.0 equiv) was used, 18.5 mg, 77% yield of alkyne **9** was obtained**.** When alkynylbenziodoxole **3f** (43.3 mg, 0.10 mmol, 1.0 equiv) was used, 19.0 mg, 79% yield of alkyne **9** was obtained. TLC: R_f = 0.61 (EtOAc / Hexanes = 1/20); ^{1}H NMR (500 MHz, $CDCl_3$) δ 8.12 (d, *J* = 8.2 Hz, 2H), 7.59 (d, *J* = 8.1 Hz, 2H), 7.32 (d, *J* = 8.4 Hz, 2H), 7.23 (d, *J* = 8.3 Hz, 2H), 2.46 (s, 3H), 2.42 (s, 3H); ^{13}C NMR (125 MHz, $CDCl_3$) δ 177.8, 145.1, 141.4, 134.7, 133.1, 129.7, 129.5, 129.3, 117.2, 93.3, 86.8, 21.8, 21.8.

Standard procedure for β-ketone alcohols. Following the literature procedure [6], β-ketone alcohol (0.20 mmol, 2.0 equiv), alkynylbenziodoxole (0.10 mmol, 1.0 equiv), $Ru(bpy)_3(PF_6)_2$ (1.7 mg, 0.002 mmol, 0.02 equiv), and BI-OAc (61.2 mg, 0.20 mmol, 2.0 equiv) were placed in a 5 mL clear-colored glass vial equipped with a magnetic stir bar. After 2.0 mL DCM were added, the vial was sealed and exposed to blue LEDs at room temperature with stirring. After TLC indicated the complete consumption of alkynylbenziodoxoles (24 h), the reaction mixture was extracted with CH_2Cl_2 and the organic layer was concentrated and purified directly by column chromatography to afford the product.

1,3-Di-p-tolylprop-2-yn-1-one 9 (CAS: 97691-66-2). Following the standard procedure for β-ketone alcohols, the reaction of β-ketone alcohol **10** (54.0 mg, 0.20 mmol, 2.0 equiv) and alkynylbenziodoxole **3a** (39.8 mg, 0.10 mmol, 1.0 equiv) for 24 h afforded ynone **9** as a white solid (14.5 mg, 62% yield) after flash chromatography. When alkynylbenziodoxole **3c** (36.2 mg, 0.10 mmol, 1.0 equiv) was used, 19.7 mg, 84% yield of alkyne **9** was obtained**.** When alkynylbenziodoxole **3f** (42.2 mg, 0.10 mmol, 1.0 equiv) was used, 19.7 mg, 84% yield of alkyne **9** was obtained.

V. References

1. Huang, H. C.; Zhang, G. J.; Gong, L.; Zhang, S. Y.; Chen, Y. Y. *J. Am. Chem. Soc*. **2014,** *136*, 2280-2283.
2. Karakaya, I.; Primer, D. N.; Molander, G. A. *Org. Lett.* **2015,** 17, 3294-3297.
3. Jia, K. F.; Zhang, F. Y.; Huang, H. C.; Chen, Y. Y. *J. Am. Chem. Soc*. **2016,** *138,* 1514-1517.
4. Hanson, R. N.; Tongcharoensirikul, P.; Barnsley, K.; Ondrechen, M. J.; Hughes, A.; DeSombre, E. R. *Steroids*, **2015,** *96*, 50-62.
5. Huang, H. C.; Zhang, G. J.; Chen, Y. Y. *Angew. Chem. Int. Ed.* **2015,** *54,* 7872-7876.
6. Jia, K. F.; Pan, Y.; Chen, Y. Y. *Angew. Chem. Int. Ed.* **2017,** *56*, 2478-2481

VI. NMR Spectra of New Compounds

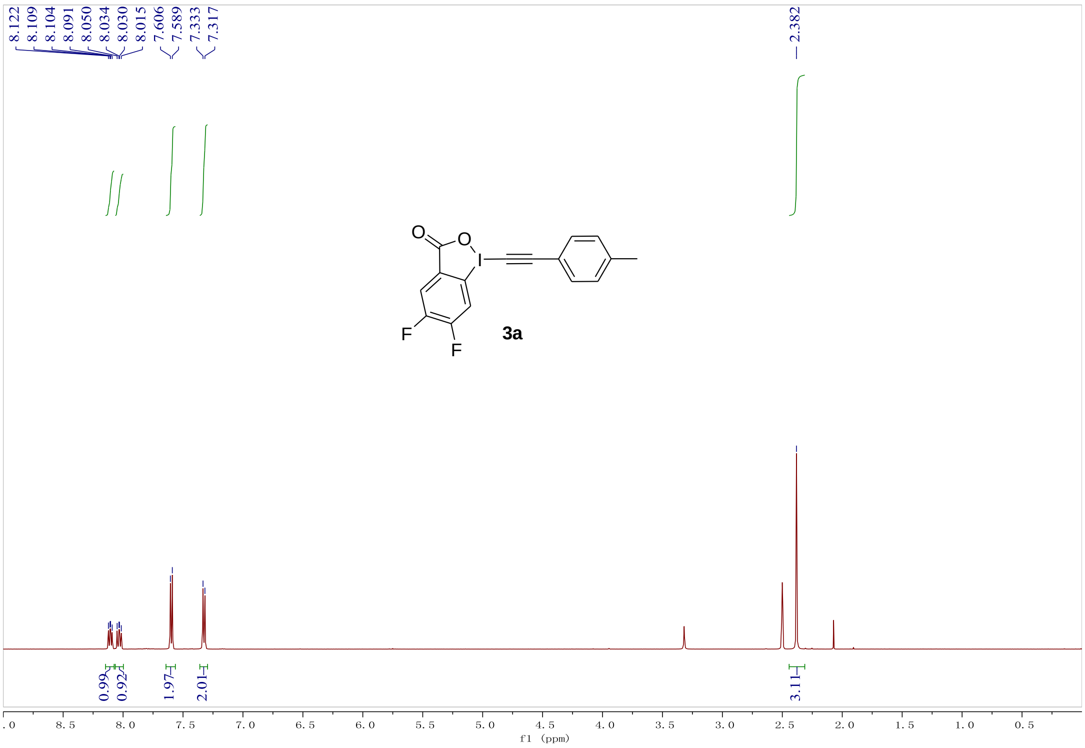

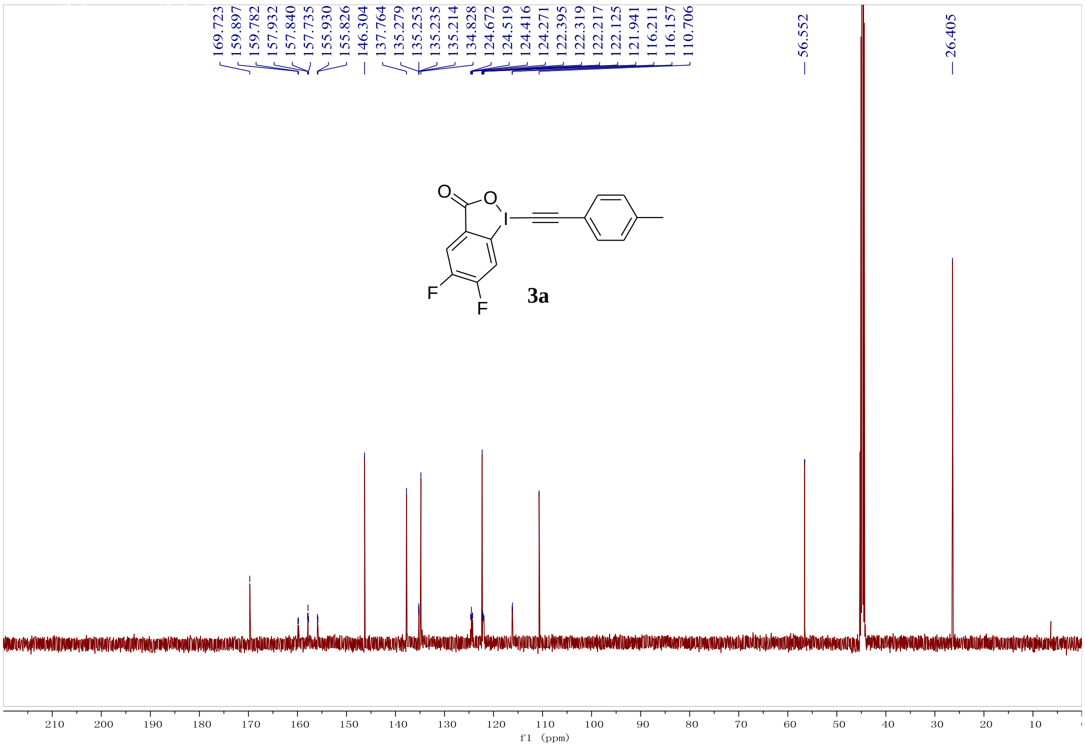

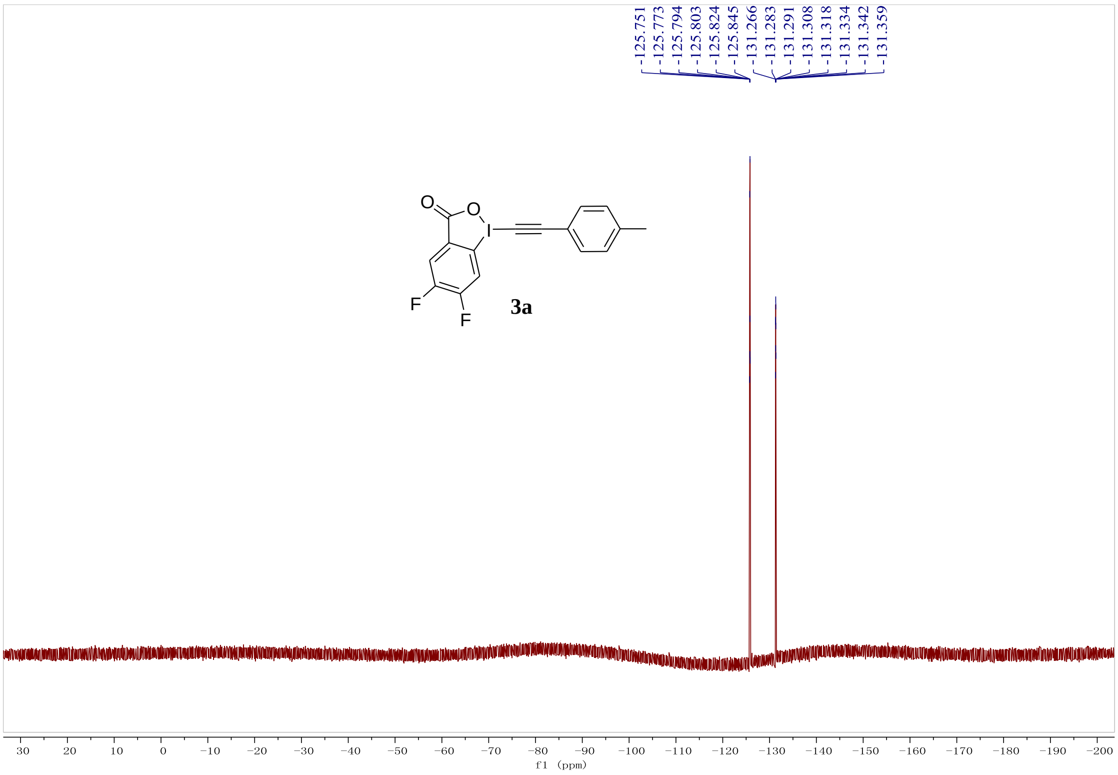

-125.751
-125.773
-125.794
-125.803
-125.824
-125.845
-131.266
-131.283
-131.291
-131.308
-131.318
-131.334
-131.342
-131.359
O
O
I
F
F
3a
30 20 10 0 −10 −20 −30 −40 −50 −60 −70 −80 −90 −100 −110 −120 −130 −140 −150 −160 −170 −180 −190 −200
f1 (ppm)

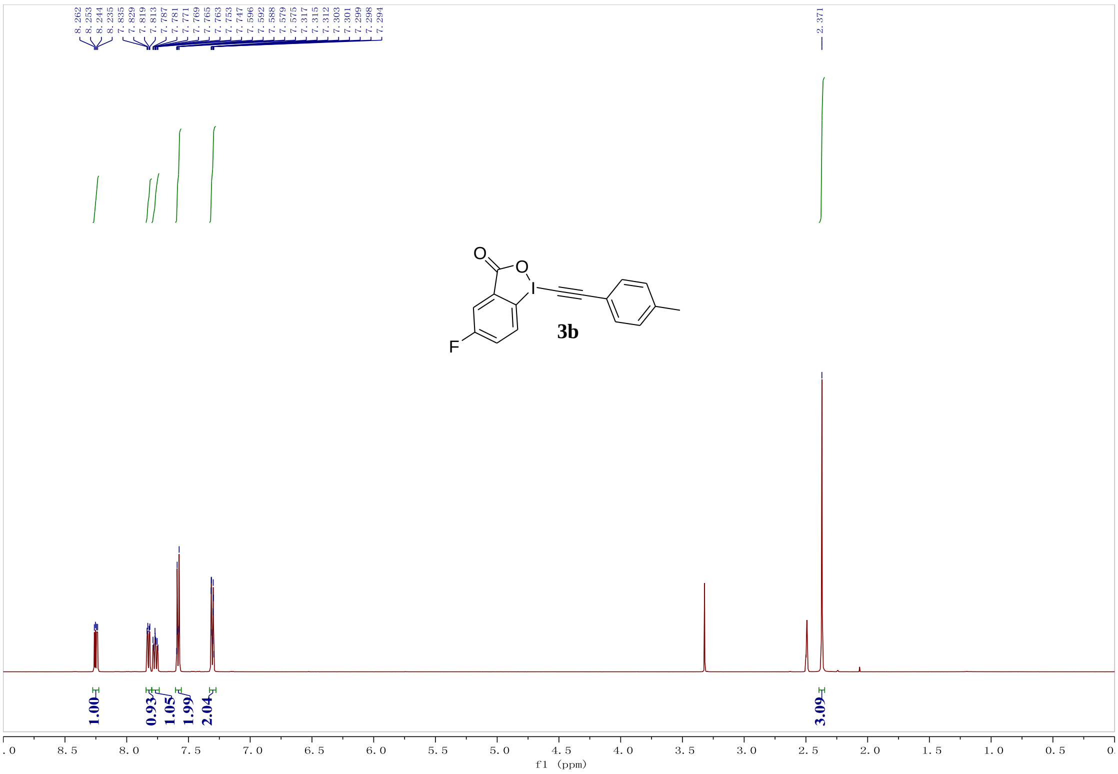

8.262
8.253
8.244
8.235
7.835
7.829
7.819
7.813
7.787
7.781
7.771
7.769
7.765
7.763
7.753
7.747
7.596
7.592
7.588
7.579
7.575
7.317
7.315
7.312
7.303
7.301
7.299
7.298
7.294
2.371
O
O
I
F
3b
1.00
0.93
1.05
1.99
2.04
3.09
8.5 8.0 7.5 7.0 6.5 6.0 5.5 5.0 4.5 4.0 3.5 3.0 2.5 2.0 1.5 1.0 0.5 0
f1 (ppm)

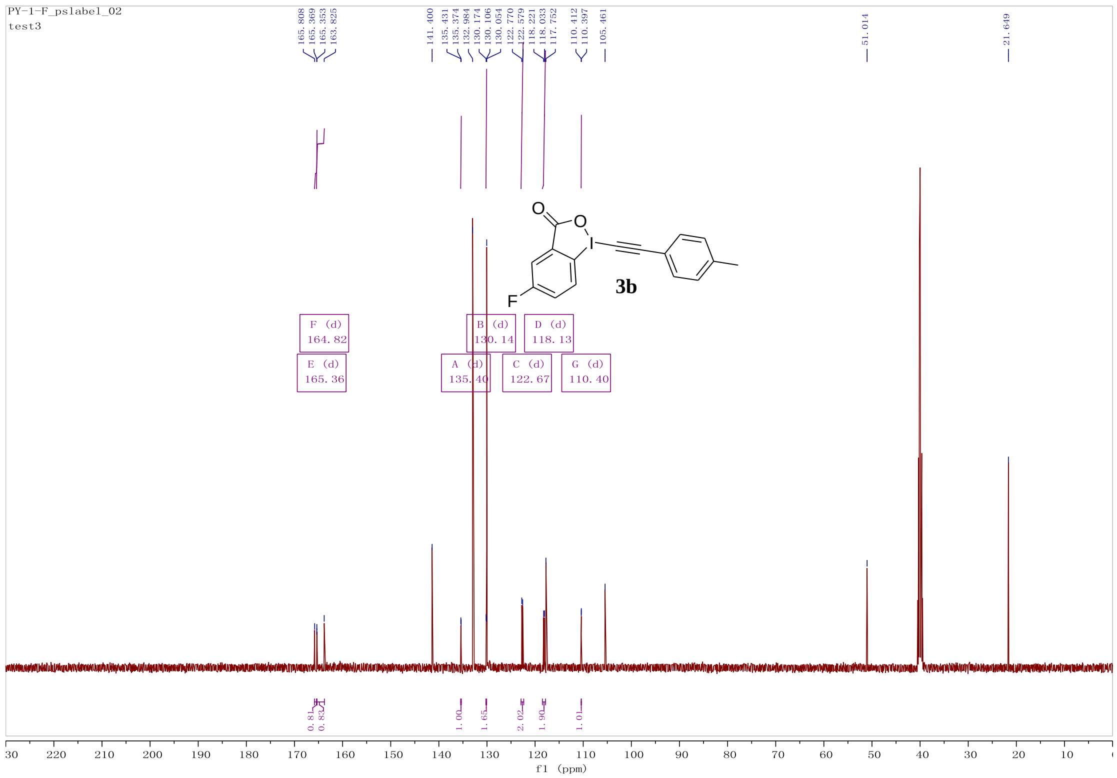
PY-1-F_pslabel_02
test3
165.898
165.369
165.353
163.825
141.400
135.431
135.374
132.984
130.174
130.106
130.054
122.770
122.579
118.221
118.033
117.752
110.412
110.397
105.461
51.014
21.649
3b
F (d)
164.82
E (d)
165.36
B (d)
130.14
A (d)
135.40
D (d)
118.13
C (d)
122.67
G (d)
110.40
0.81
0.83
1.00
1.65
2.02
1.90
1.01
f1 (ppm)

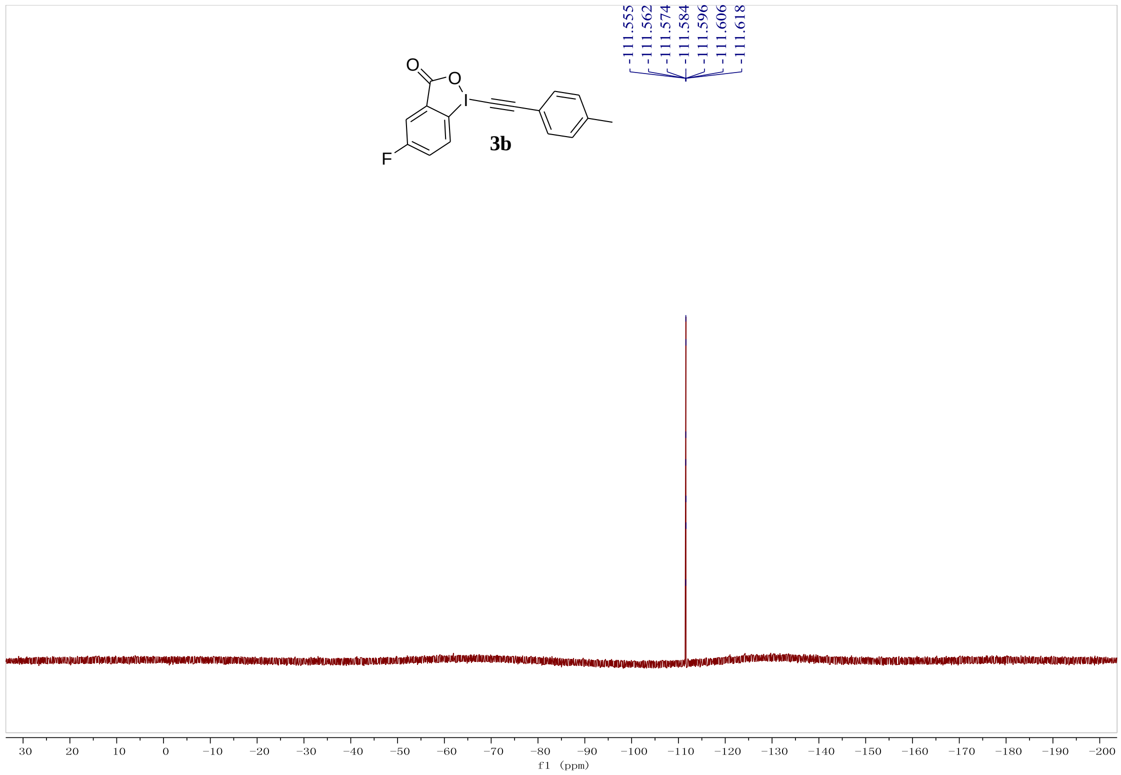
-111.555
-111.562
-111.574
-111.584
-111.596
-111.606
-111.618
3b
f1 (ppm)

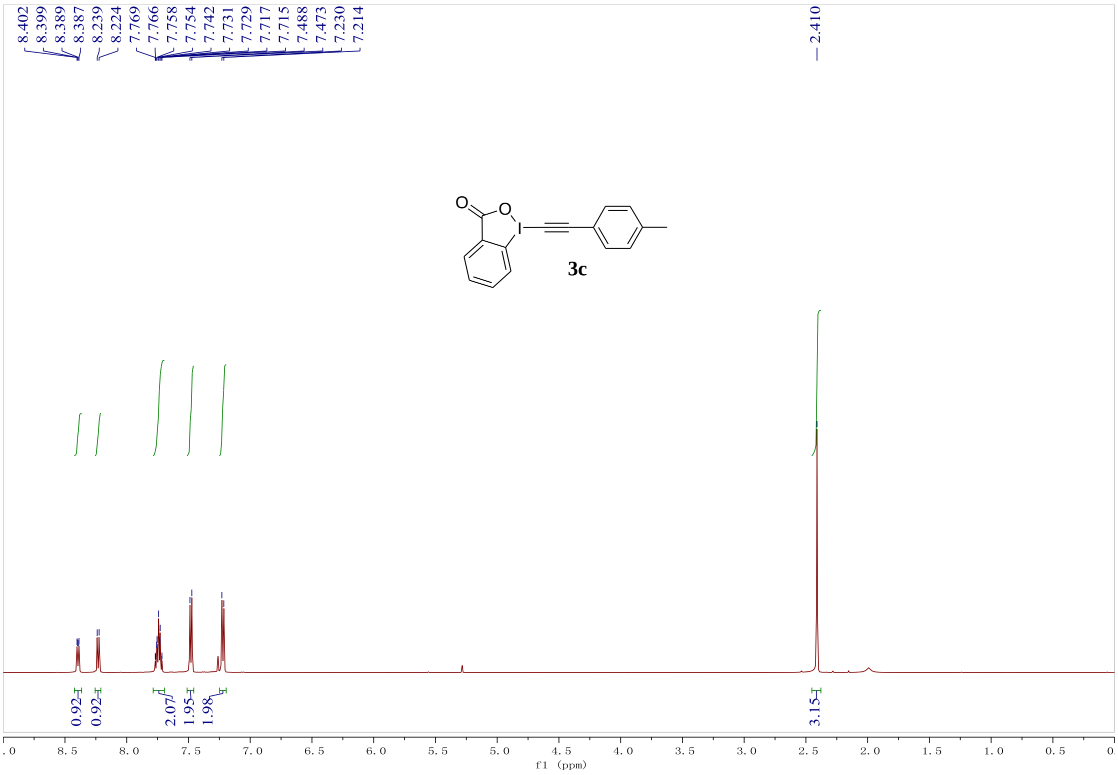
8.402
8.399
8.389
8.387
8.239
8.224
7.769
7.766
7.758
7.754
7.742
7.731
7.729
7.717
7.715
7.488
7.473
7.230
7.214
2.410
O
O
I
3c
0.92
0.92
2.07
1.95
1.98
3.15
f1 (ppm)

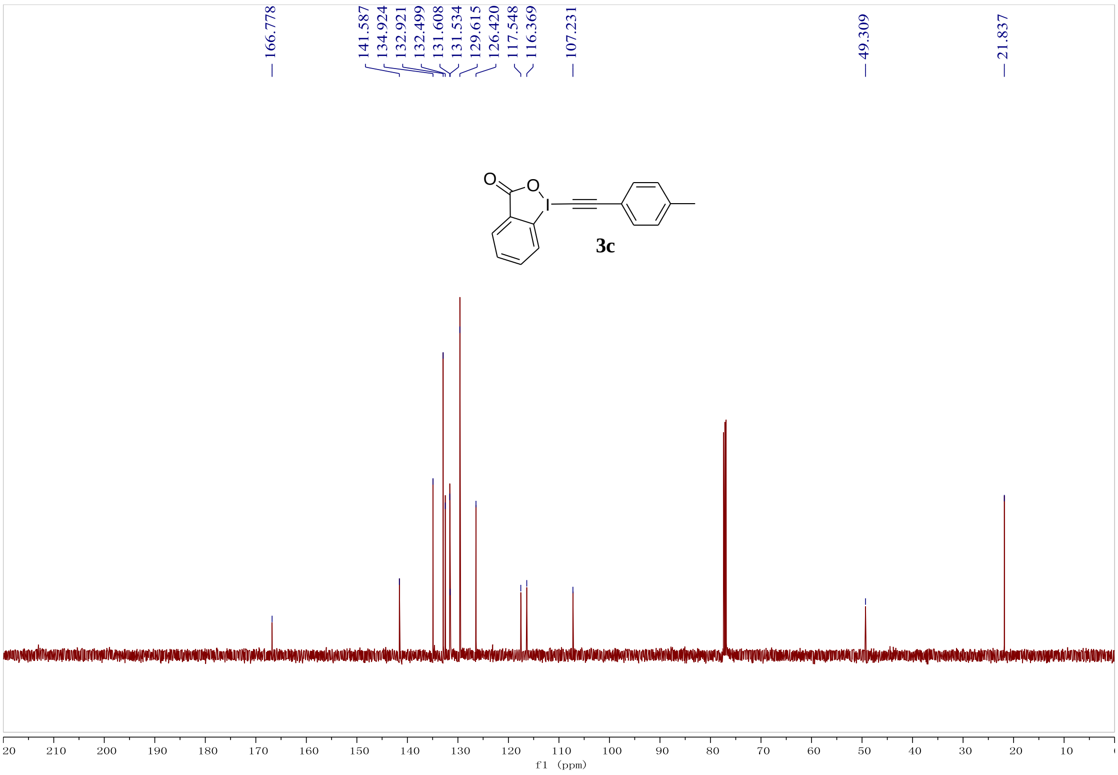
166.778
141.587
134.924
132.921
132.499
131.608
131.534
129.615
126.420
117.548
116.369
107.231
49.309
21.837
O
O
I
3c
f1 (ppm)

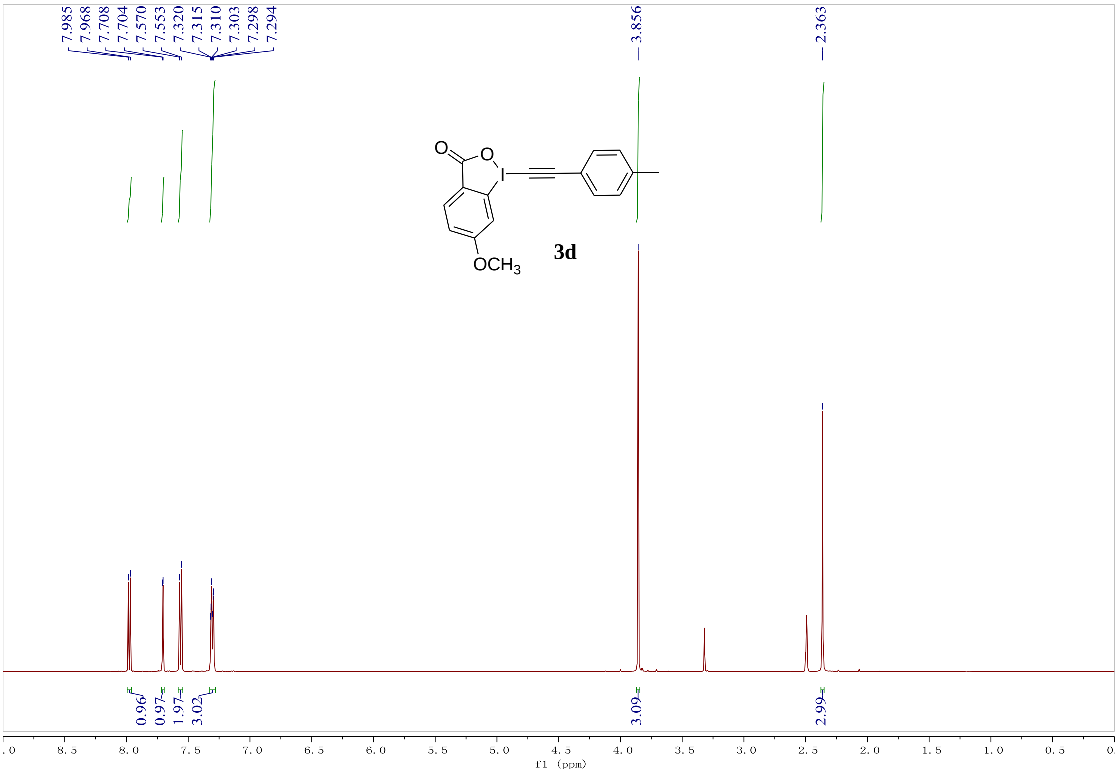

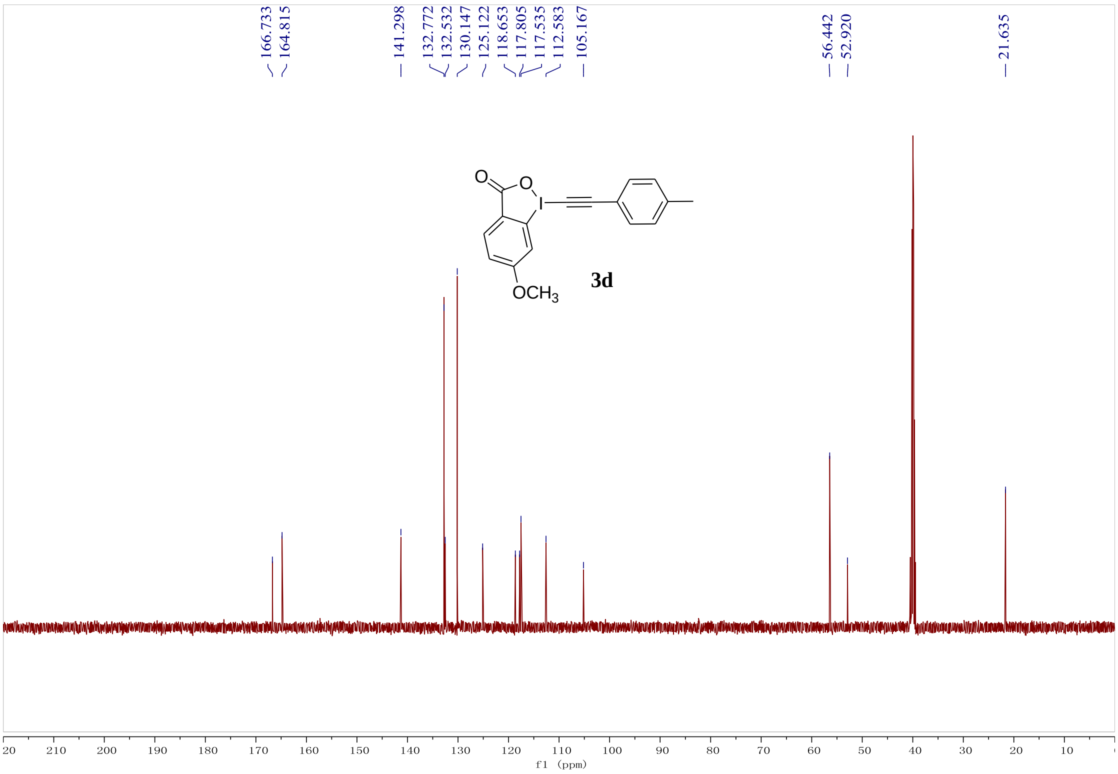

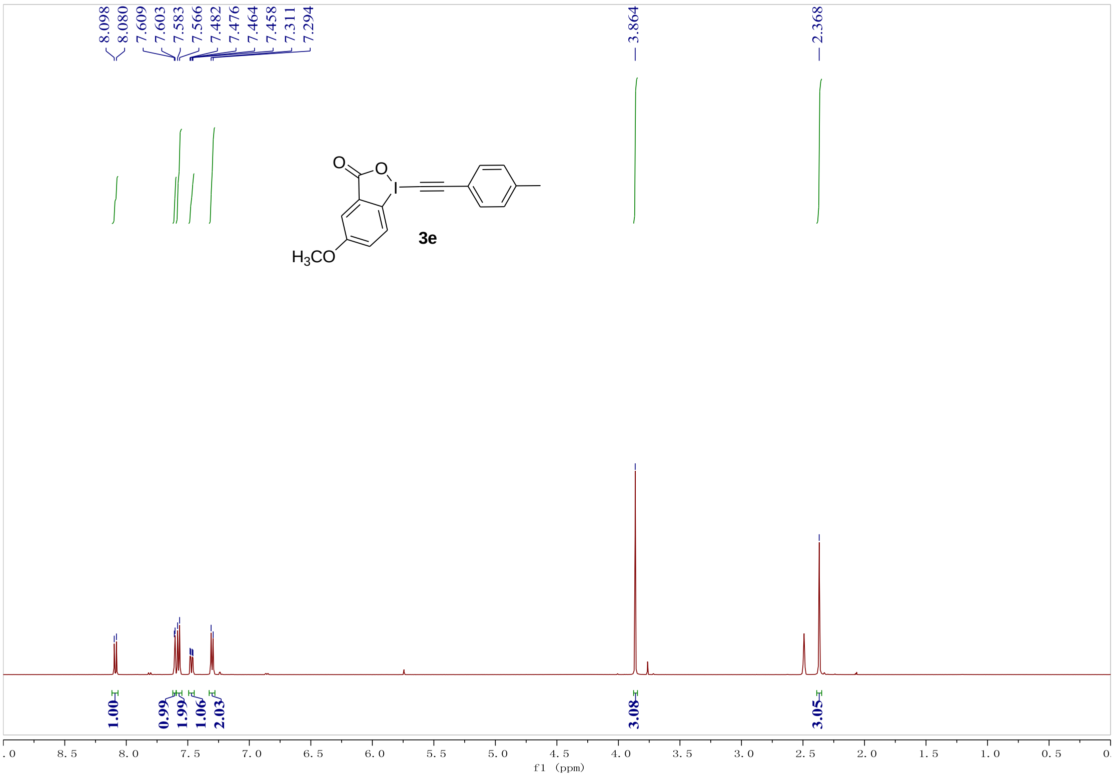
8.098
8.080
7.609
7.603
7.583
7.566
7.482
7.476
7.464
7.458
7.311
7.294
3.864
2.368
H3CO
3e
1.00
0.99
1.99
1.06
2.03
3.08
3.05
f1 (ppm)

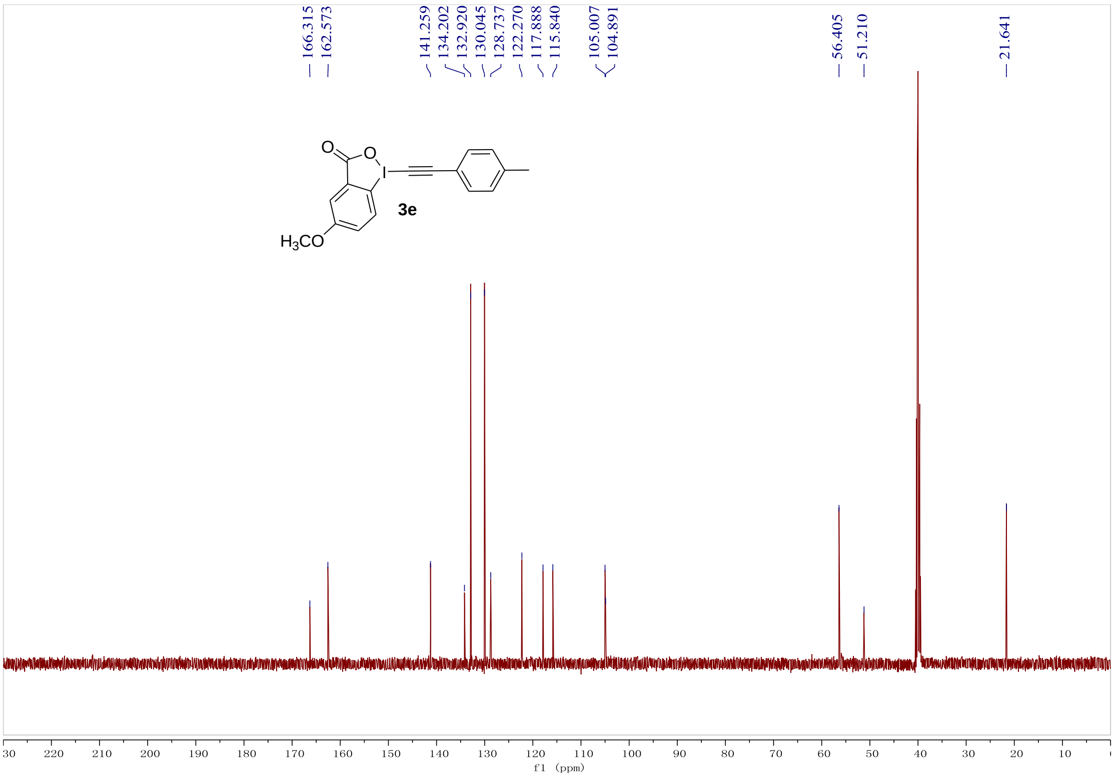
166.315
162.573
141.259
134.202
132.920
130.045
128.737
122.270
117.888
115.840
105.007
104.891
56.405
51.210
21.641
H3CO
3e
f1 (ppm)

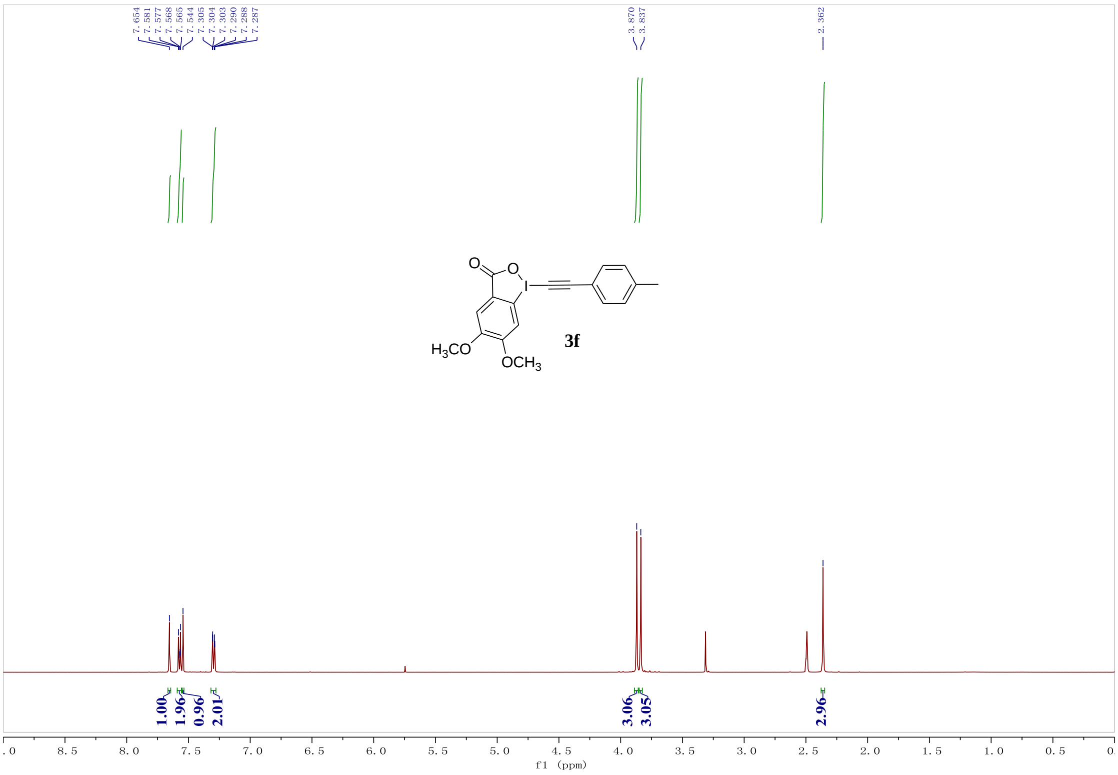
7.654
7.581
7.577
7.568
7.565
7.544
7.305
7.304
7.303
7.290
7.288
7.287
3.870
3.837
2.362
H3CO
OCH3
3f
1.00
1.96
0.96
2.01
3.06
3.05
2.96
f1 (ppm)

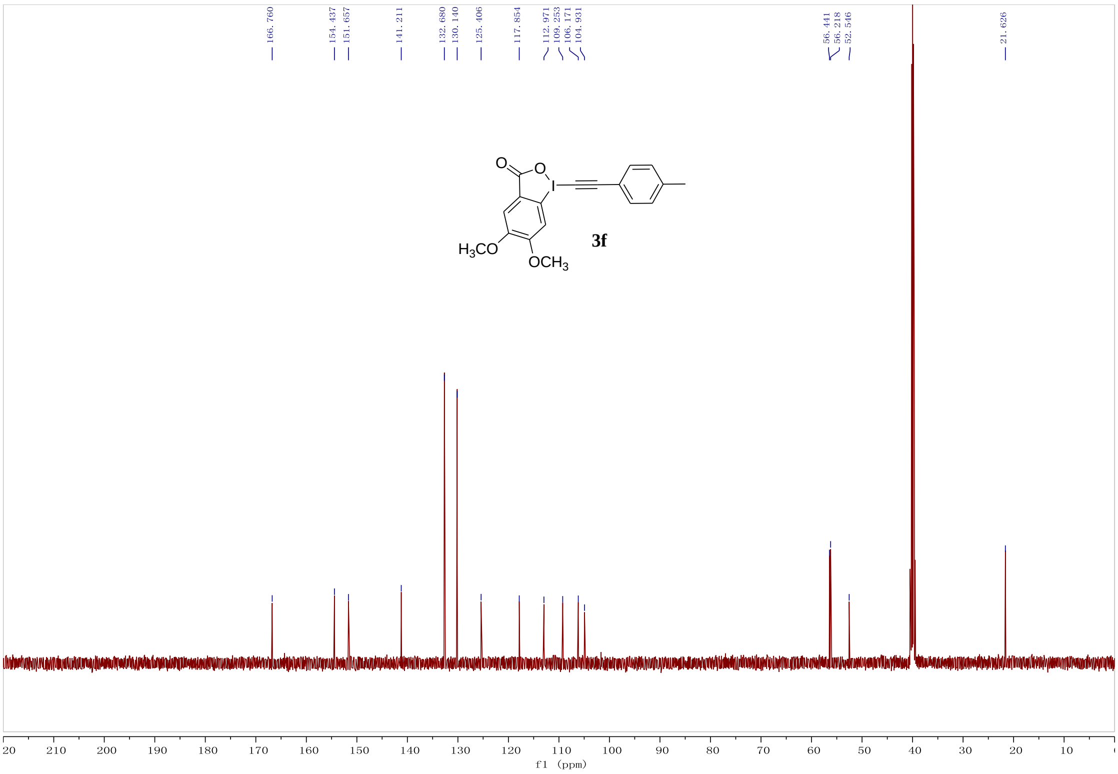
166.760
154.437
151.657
141.211
132.680
130.140
125.406
117.854
112.971
109.253
106.171
104.931
56.441
56.218
52.546
21.626
H3CO
OCH3
3f
f1 (ppm)

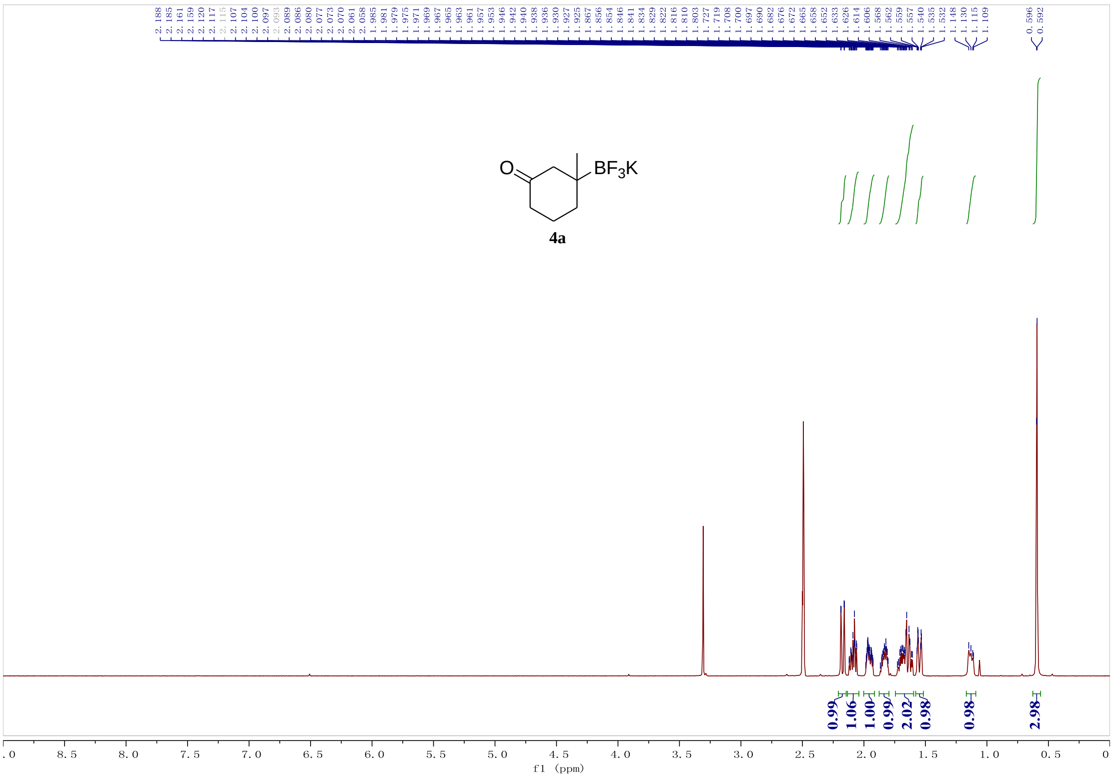
4a

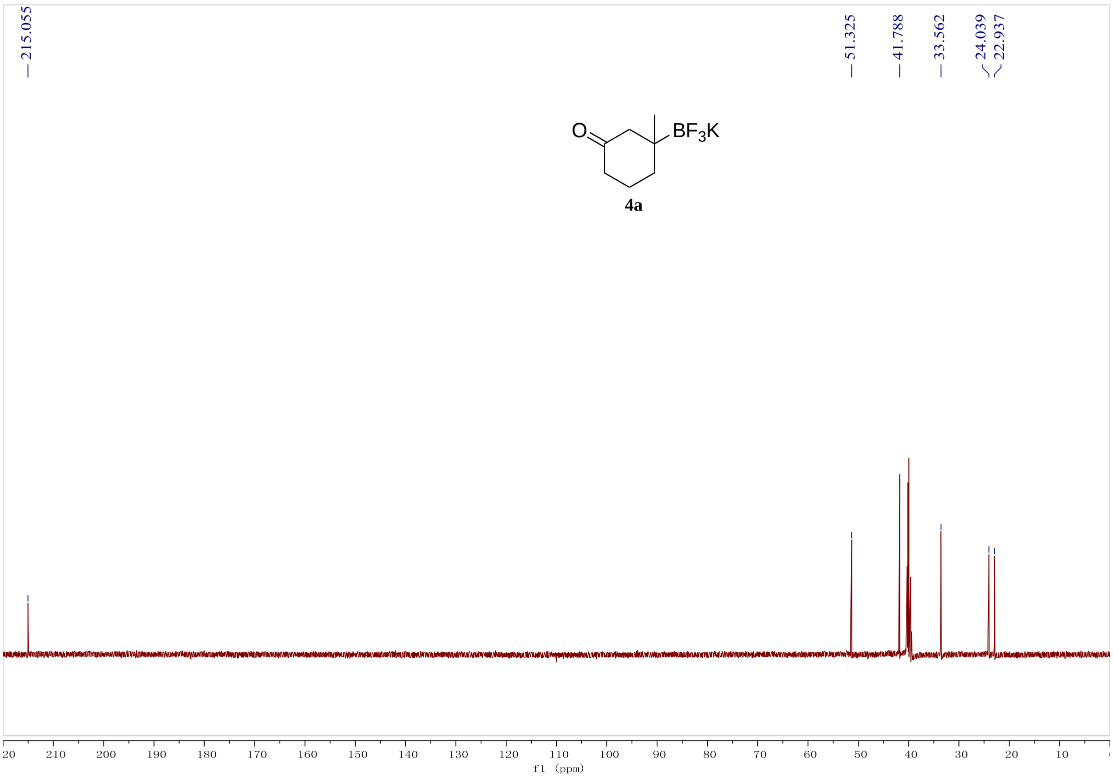
215.055
51.325
41.788
33.562
24.039
22.937
4a

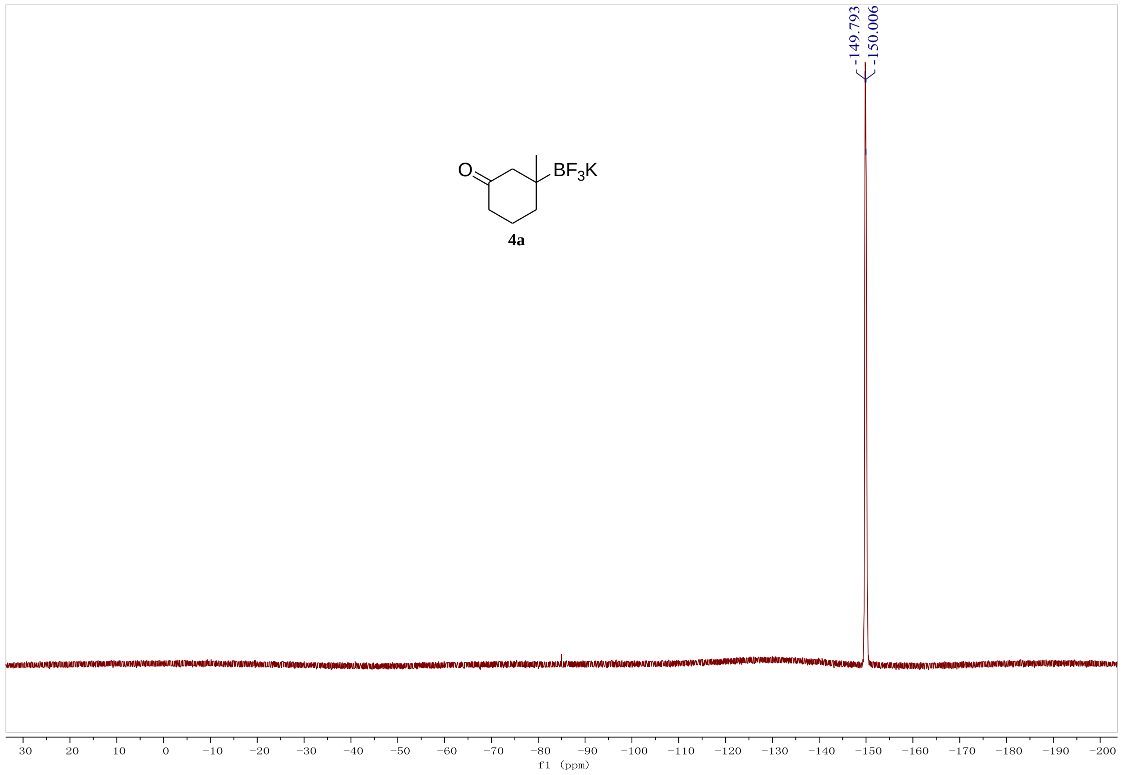

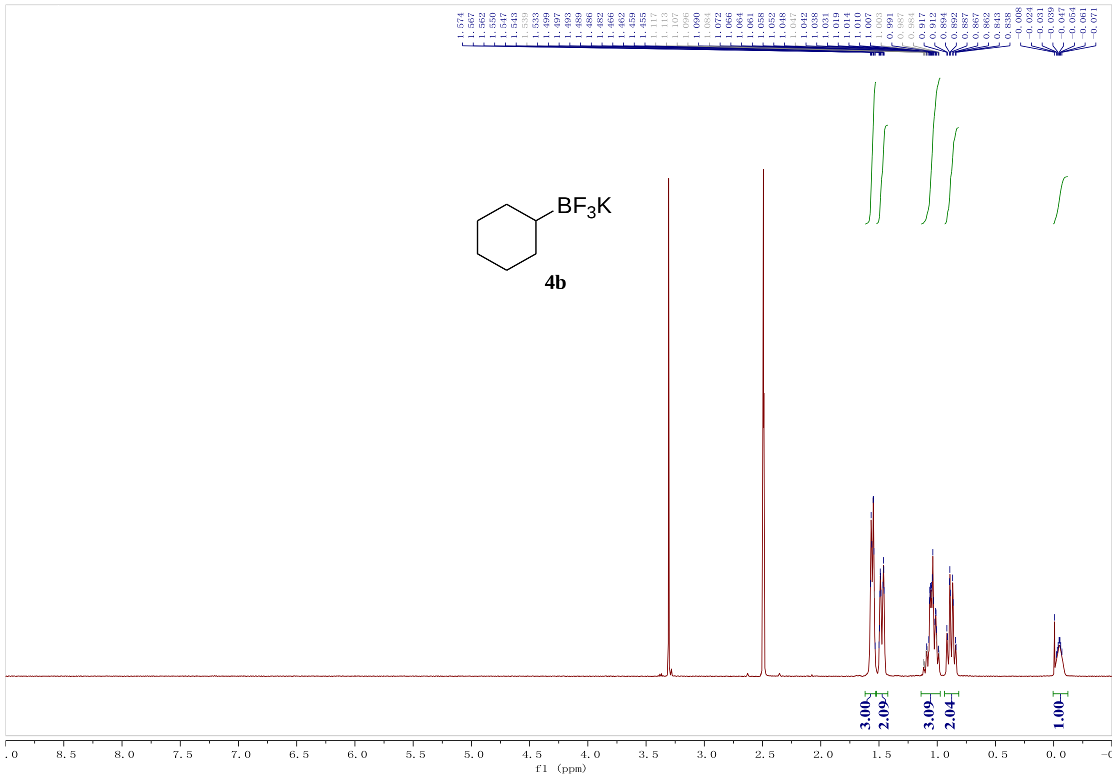

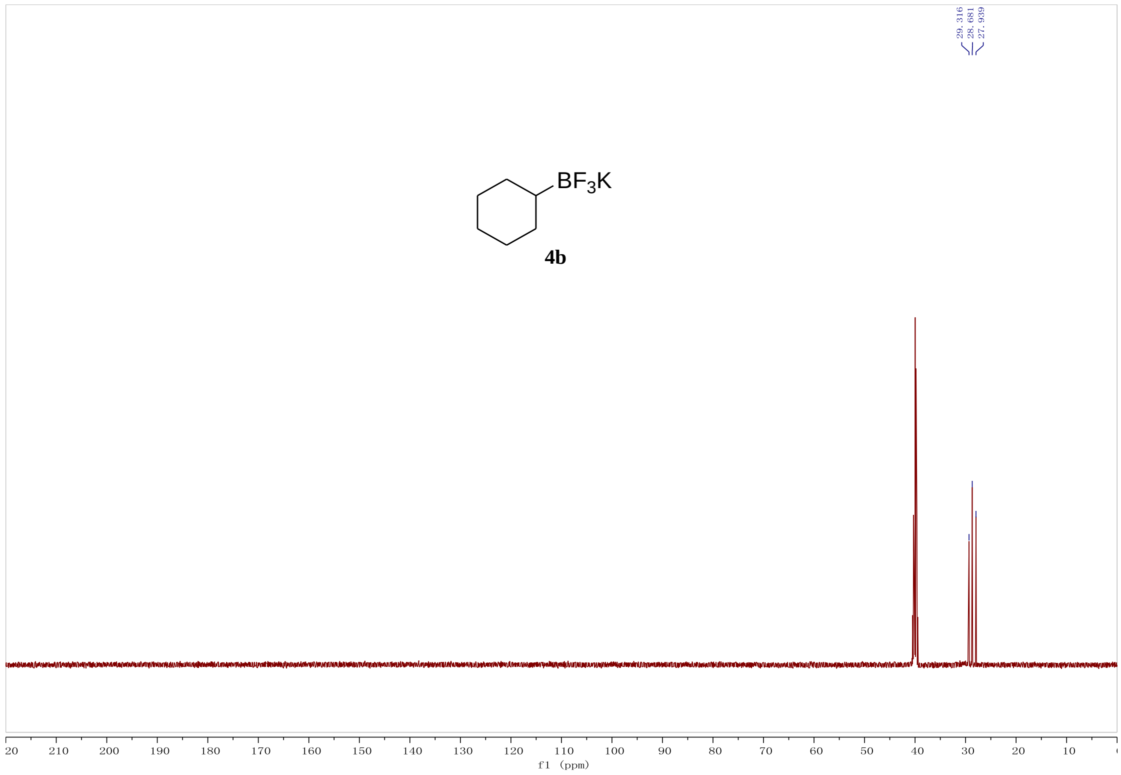
29.316
28.681
27.939
BF3K
4b
f1 (ppm)

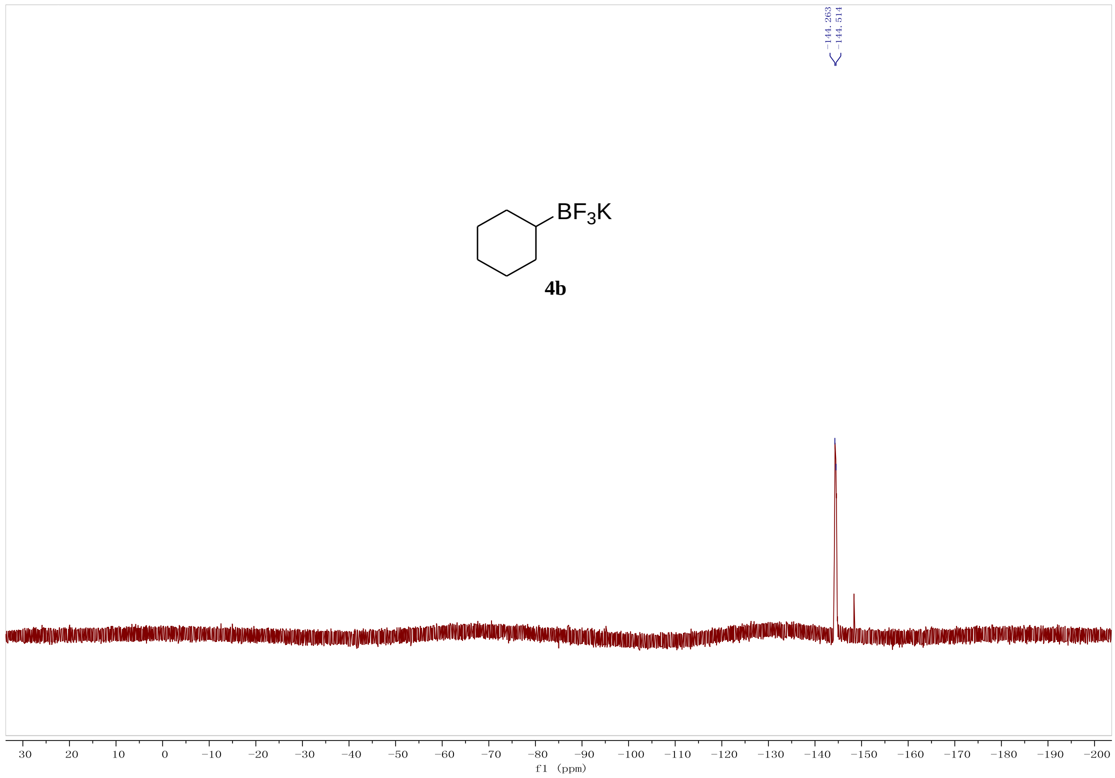
-144.263
-144.514
BF3K
4b
f1 (ppm)

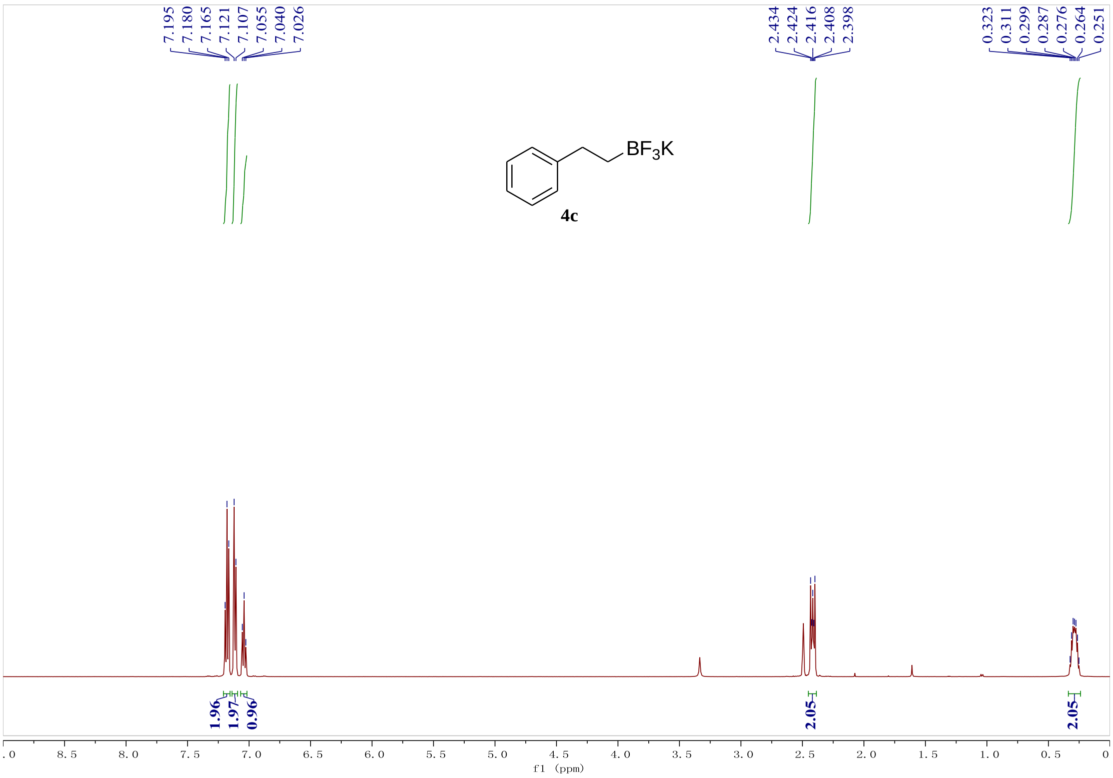
7.195
7.180
7.165
7.121
7.107
7.055
7.040
7.026
2.434
2.424
2.416
2.408
2.398
0.323
0.311
0.299
0.287
0.276
0.264
0.251
BF3K
4c
1.96
1.97
0.96
2.05
2.05
f1 (ppm)

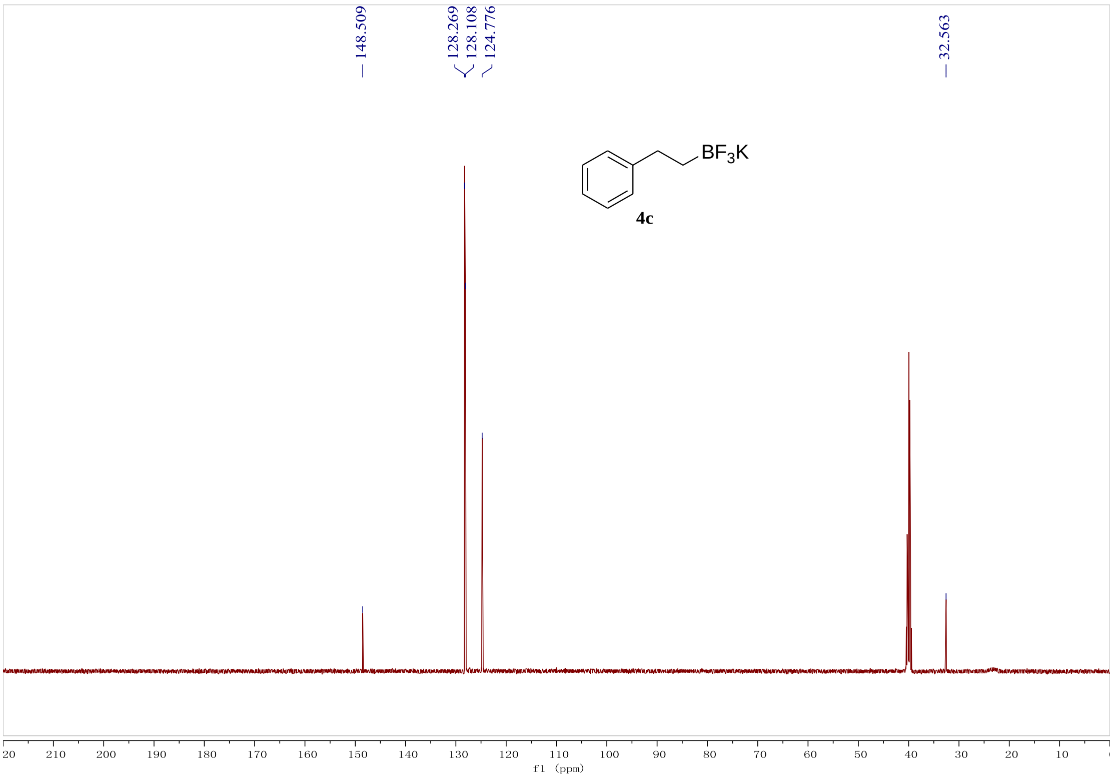
148.509
128.269
128.108
124.776
32.563
BF3K
4c
f1 (ppm)

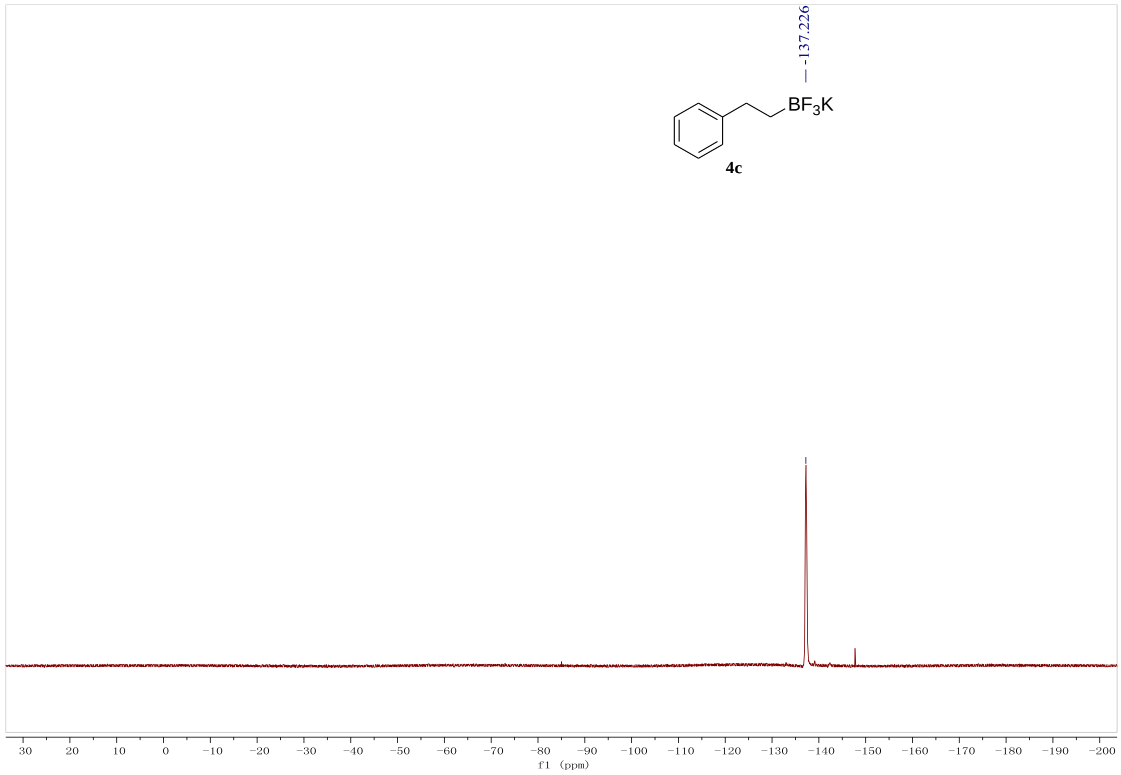
-137.226
BF3K
4c
f1 (ppm)

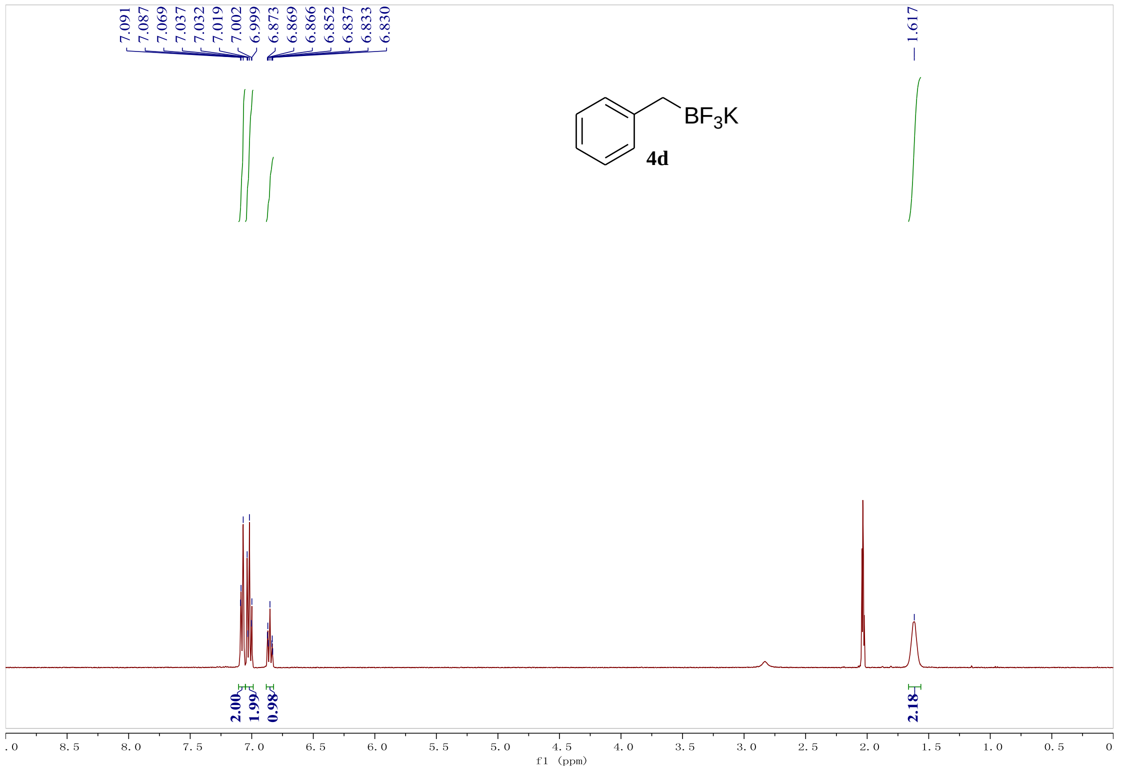
7.091
7.087
7.069
7.037
7.032
7.019
7.002
6.999
6.873
6.869
6.866
6.852
6.837
6.833
6.830
1.617
BF3K
4d
2.00
1.99
0.98
2.18
f1 (ppm)

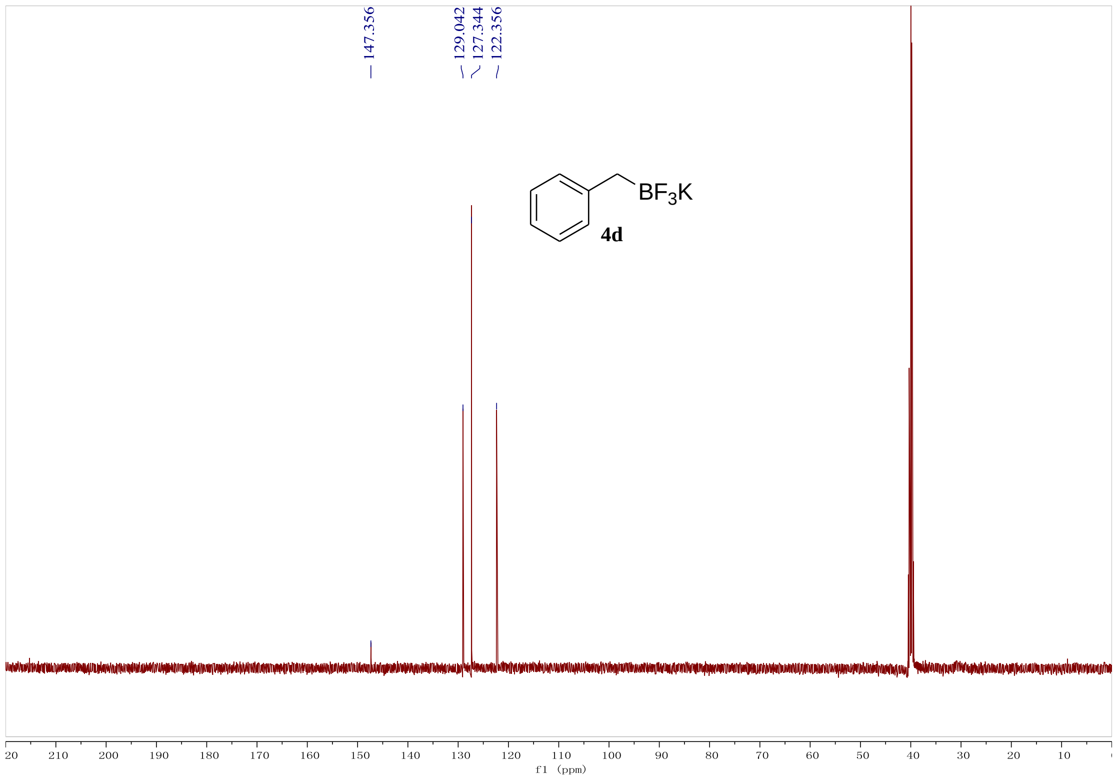
147.356
129.042
127.344
122.356
BF3K
4d
f1 (ppm)

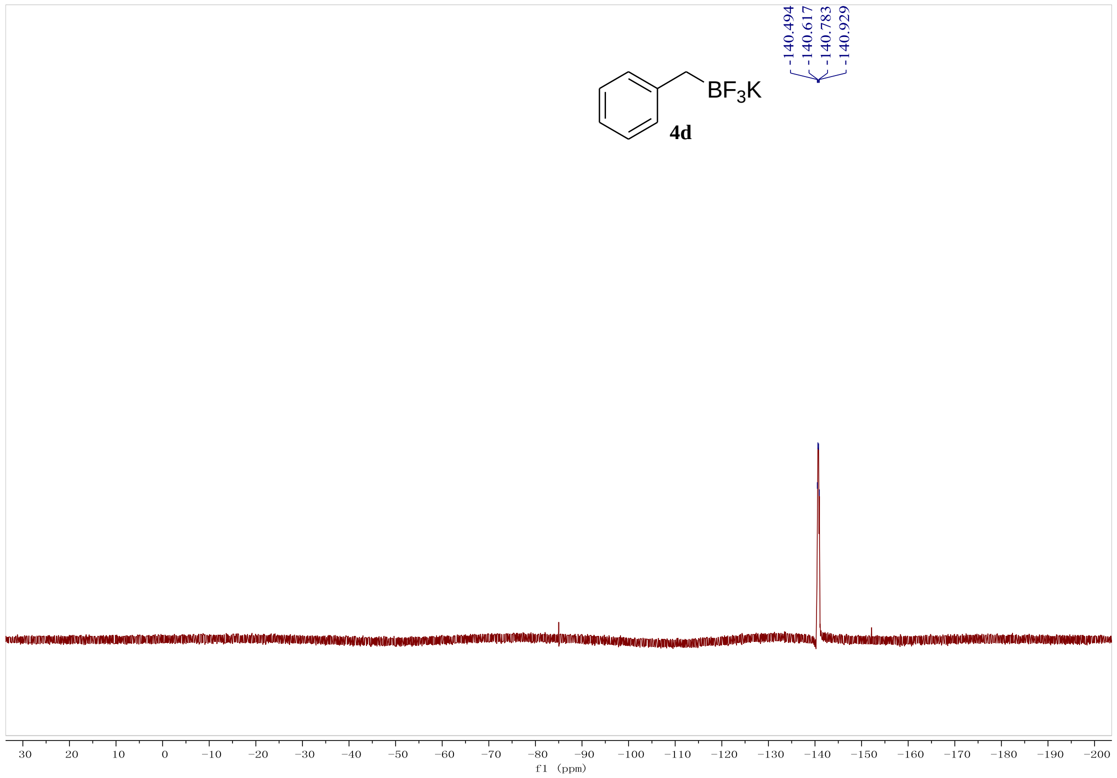
-140.494
-140.617
-140.783
-140.929
BF3K
4d
f1 (ppm)

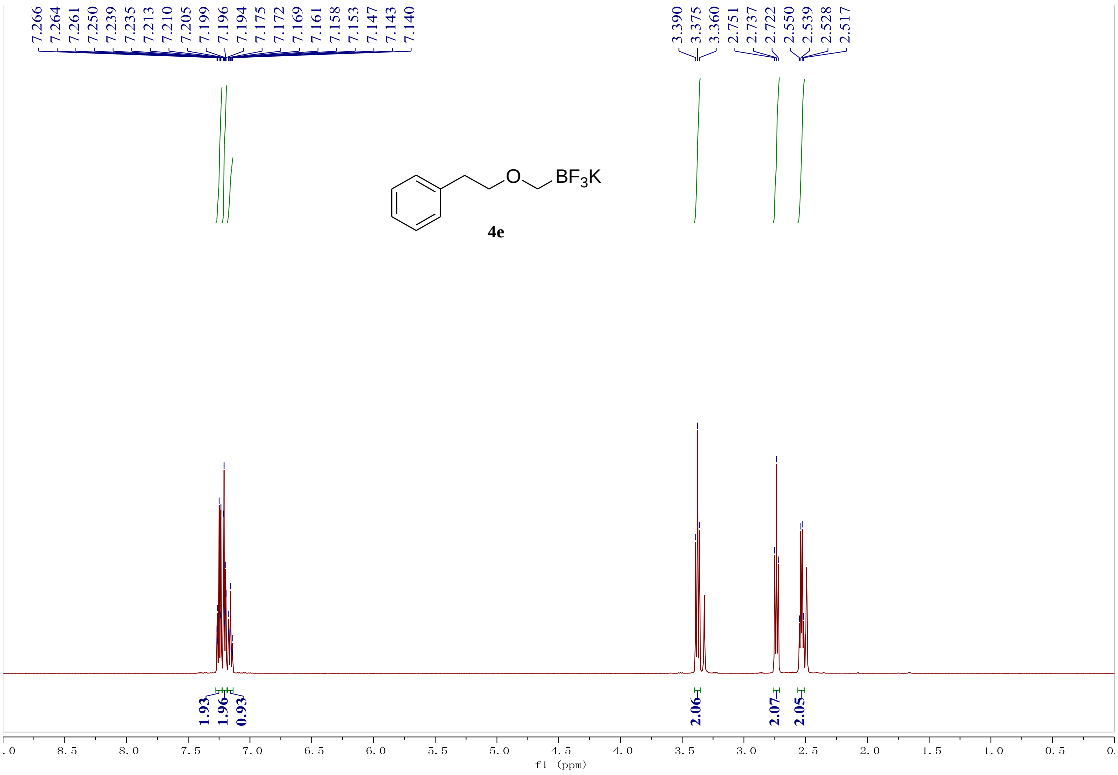

7.266
7.264
7.261
7.250
7.239
7.235
7.213
7.210
7.205
7.199
7.196
7.194
7.175
7.172
7.169
7.161
7.158
7.153
7.147
7.143
7.140
3.390
3.375
3.360
2.751
2.737
2.722
2.550
2.539
2.528
2.517
BF_3K
4e
1.93
1.96
0.93
2.06
2.07
2.05
f1 (ppm)

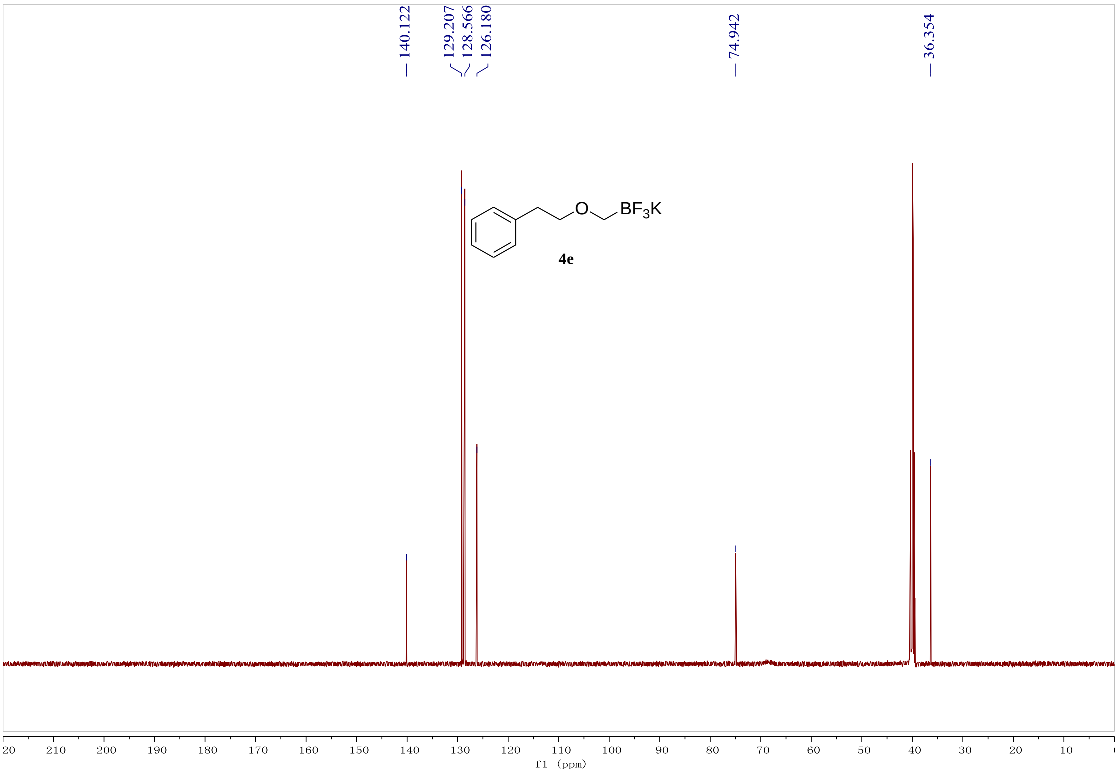

140.122
129.207
128.566
126.180
74.942
36.354
BF_3K
4e
f1 (ppm)

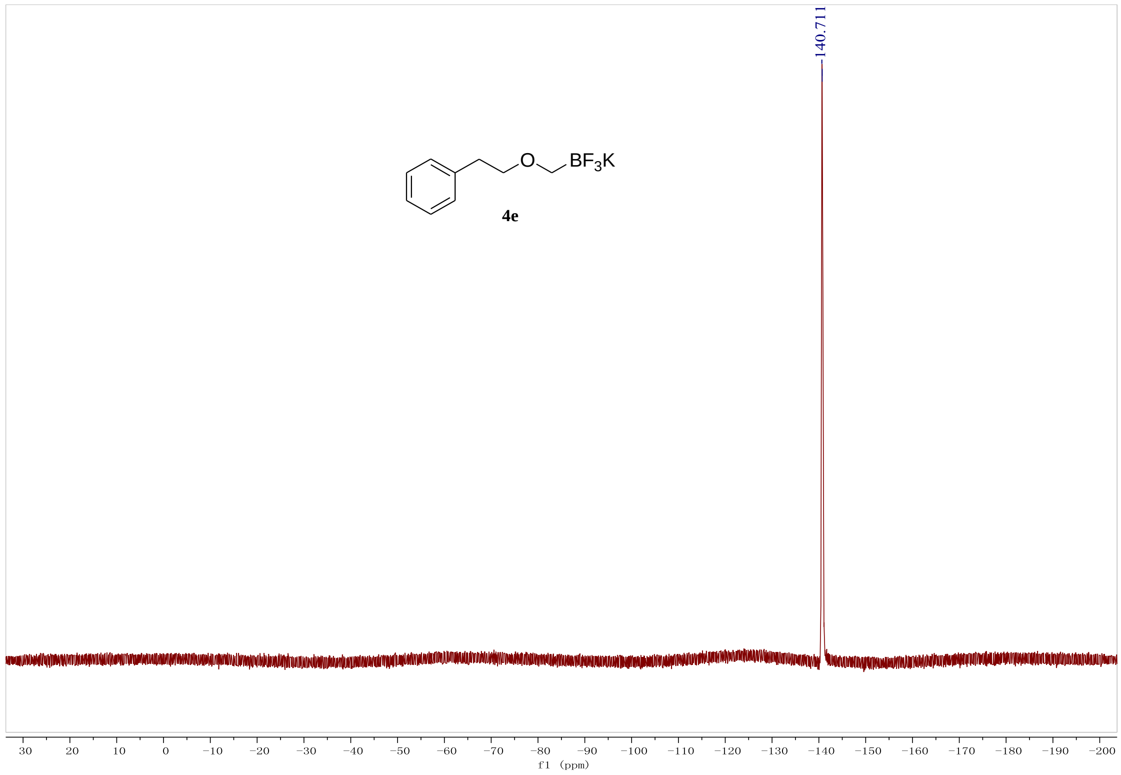

-140.711
BF3K
O
4e
f1 (ppm)

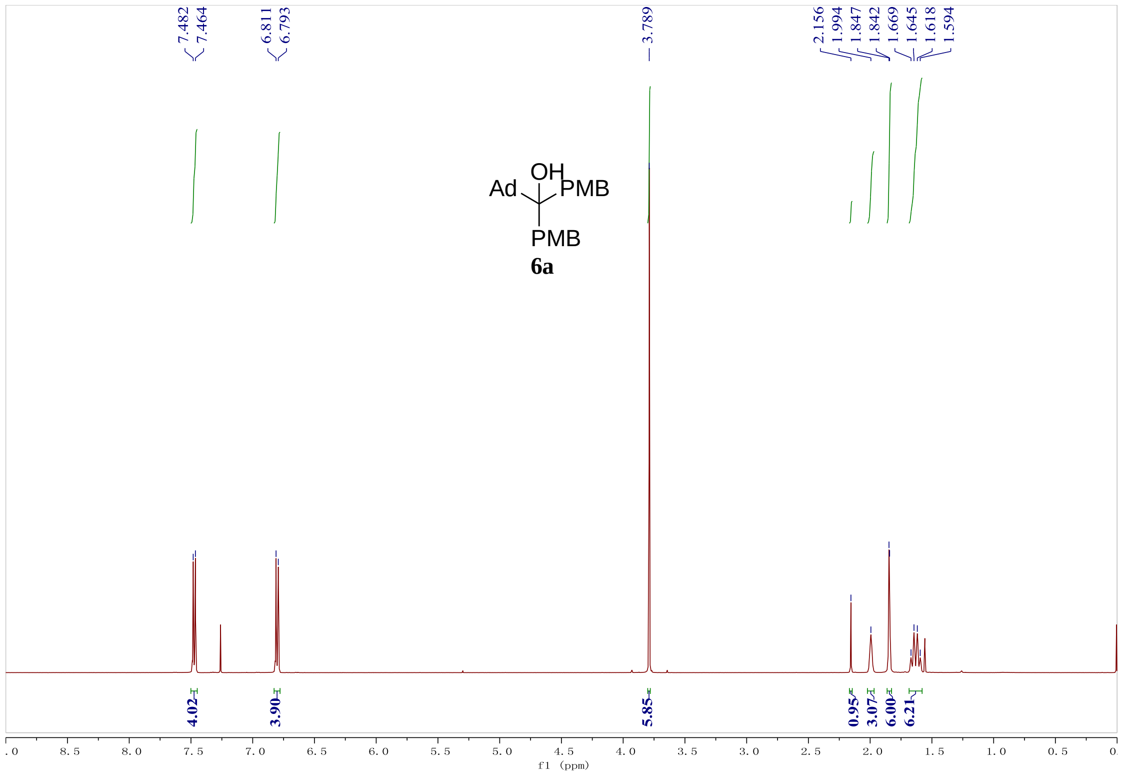

7.482
7.464
6.811
6.793
3.789
2.156
1.994
1.847
1.842
1.669
1.645
1.618
1.594
Ad
OH
PMB
PMB
6a
4.02
3.90
5.85
0.95
3.07
6.00
6.21
f1 (ppm)

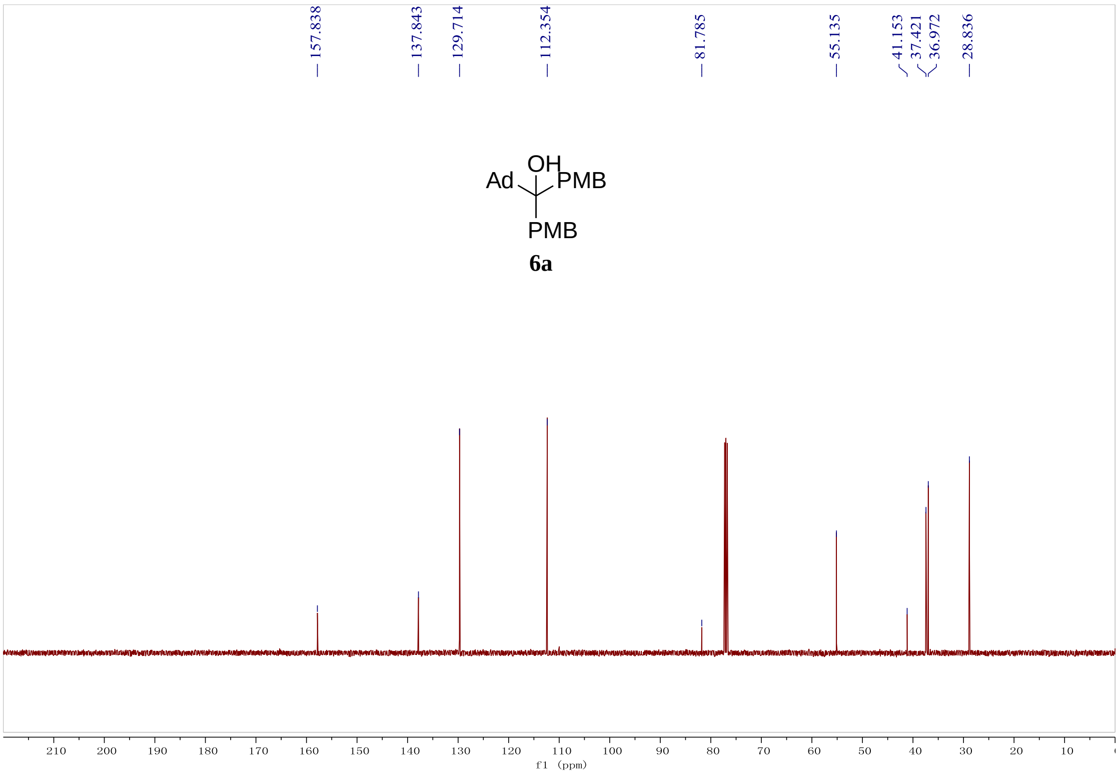

157.838
137.843
129.714
112.354
81.785
55.135
41.153
37.421
36.972
28.836
Ad
OH
PMB
PMB
6a
f1 (ppm)

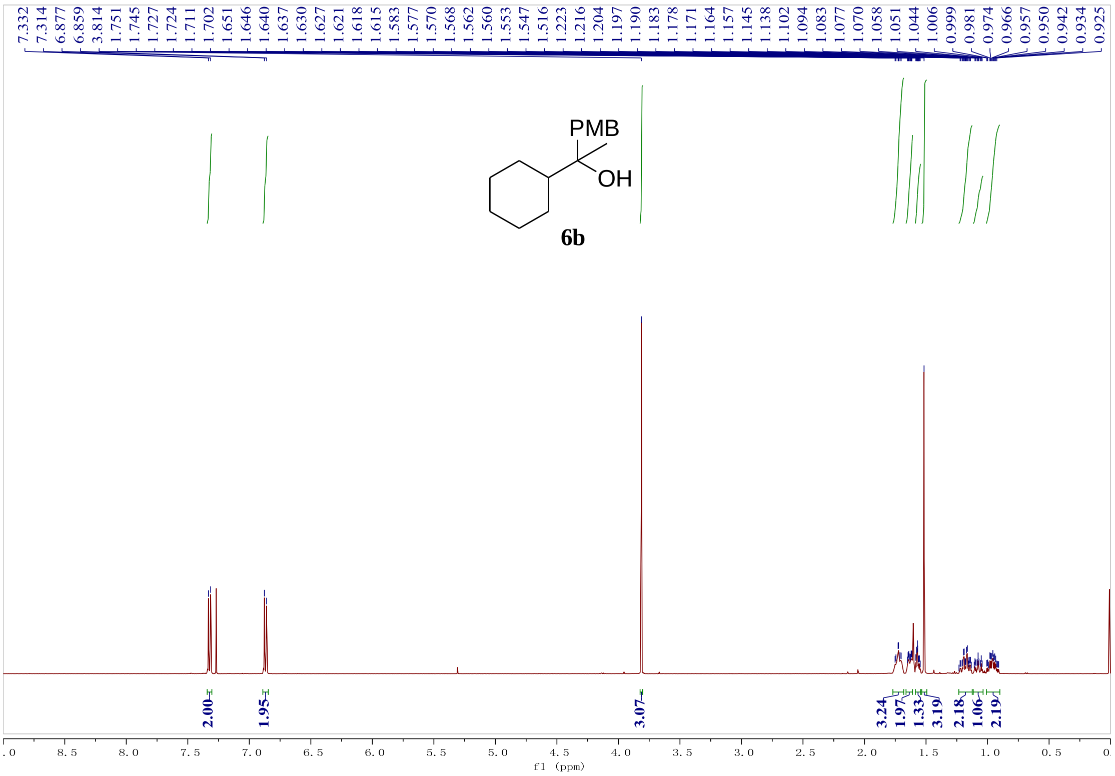

PMB
OH
6b
2.00
1.95
3.07
3.24
1.97
1.33
3.19
2.18
1.06
2.19
f1 (ppm)

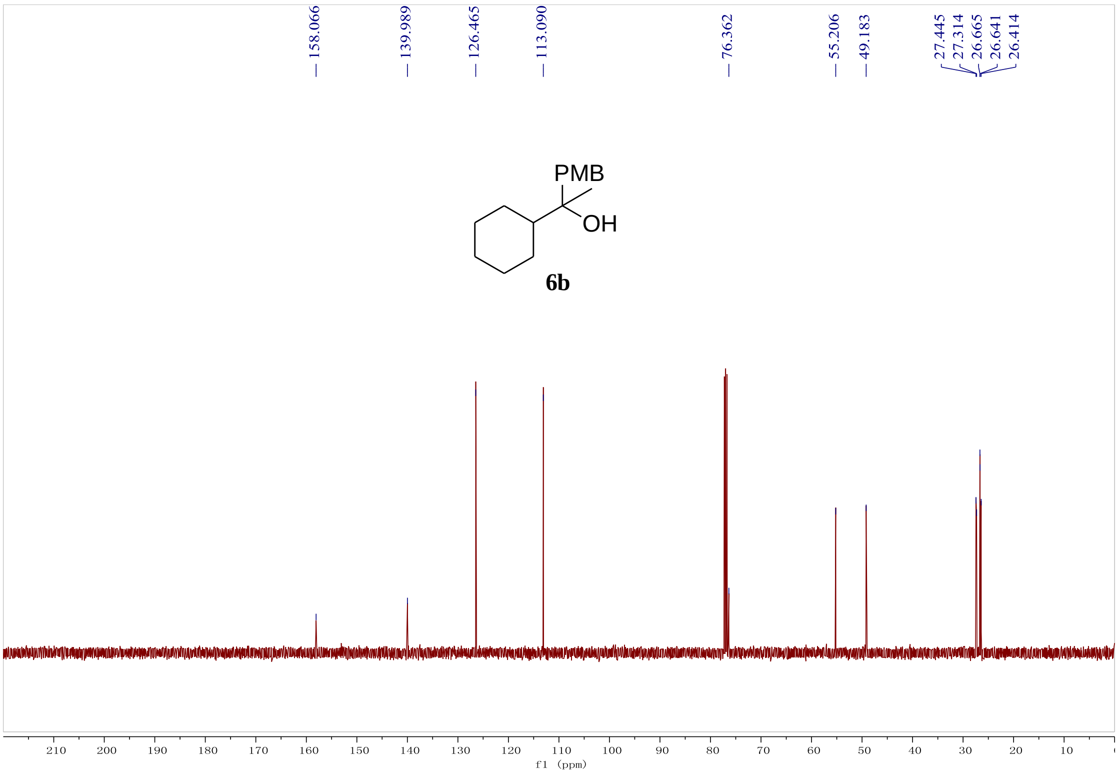
158.066
139.989
126.465
113.090
76.362
55.206
49.183
27.445
27.314
26.665
26.641
26.414
PMB
OH
6b
210 200 190 180 170 160 150 140 130 120 110 100 90 80 70 60 50 40 30 20 10
f1 (ppm)

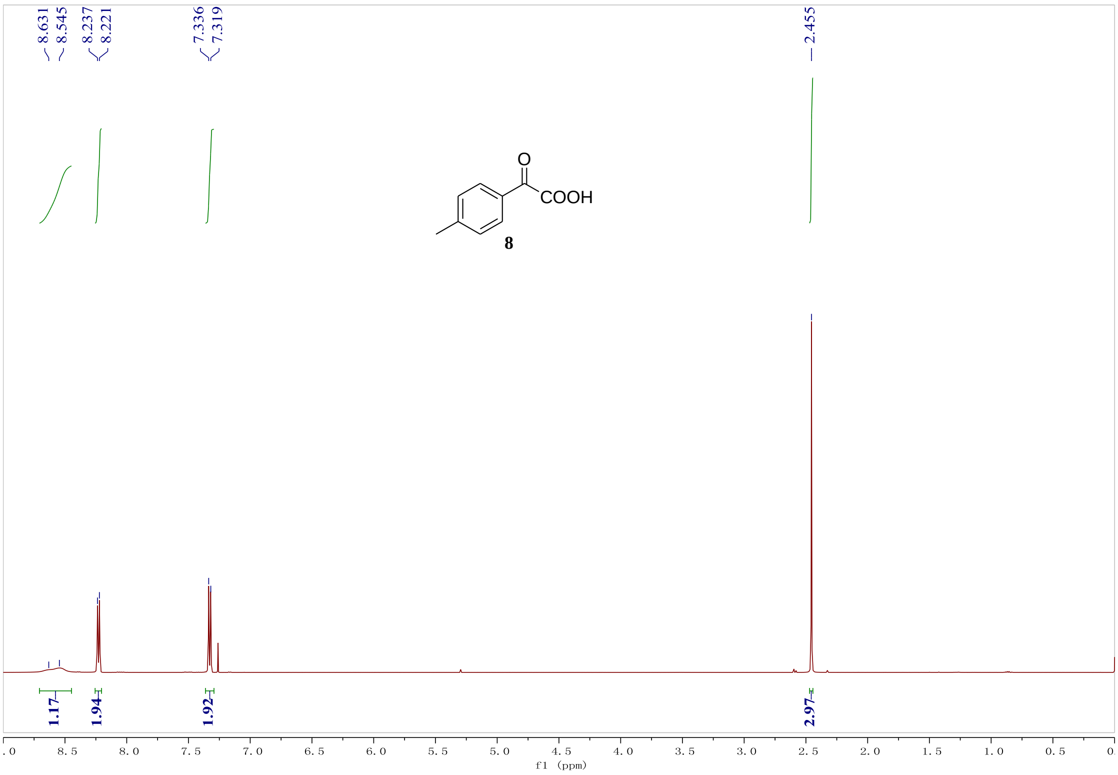
8.631
8.545
8.237
8.221
7.336
7.319
2.455
O
COOH
8
1.17
1.94
1.92
2.97
8.5 8.0 7.5 7.0 6.5 6.0 5.5 5.0 4.5 4.0 3.5 3.0 2.5 2.0 1.5 1.0 0.5
f1 (ppm)

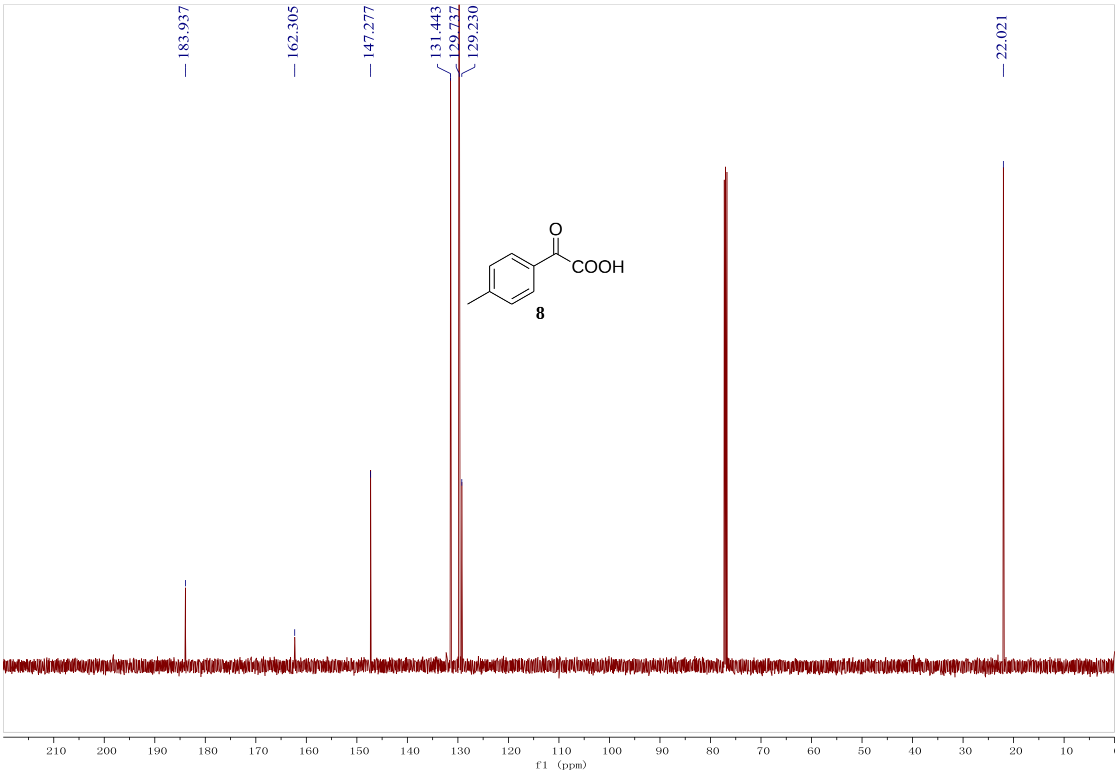
183.937
162.305
147.277
131.443
129.737
129.230
22.021
O
COOH
8
f1 (ppm)

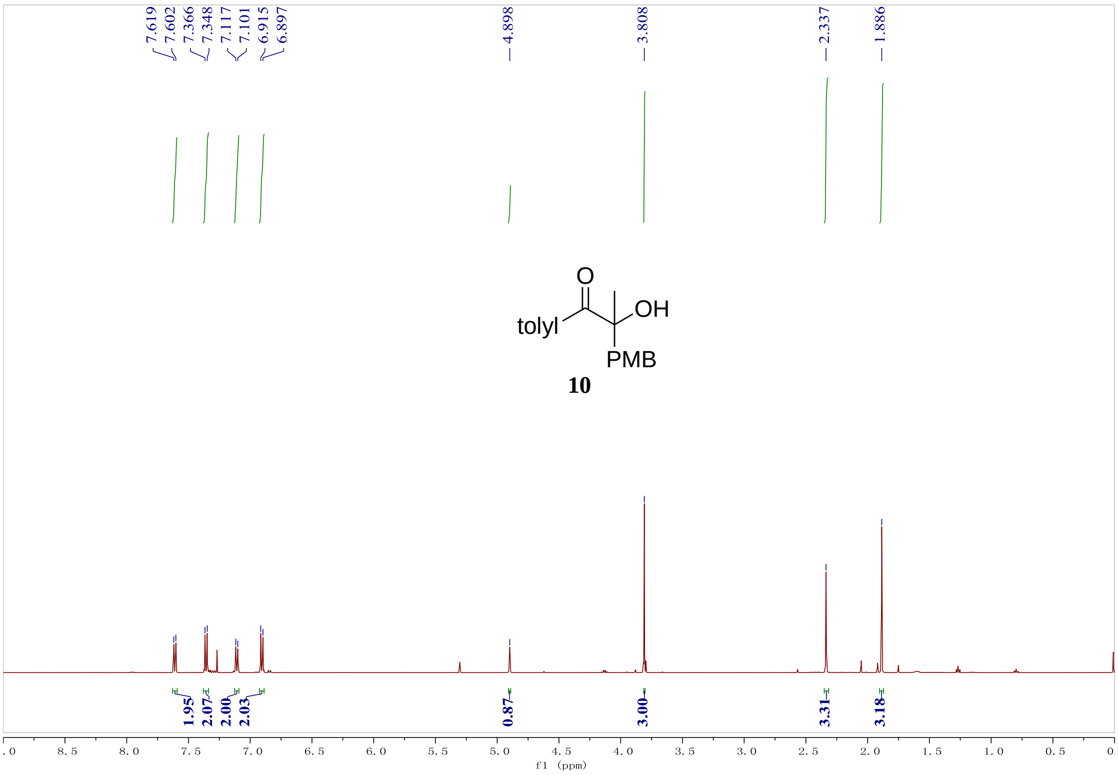
7.619
7.602
7.366
7.348
7.117
7.101
6.915
6.897
4.898
3.808
2.337
1.886
O
OH
tolyl
PMB
10
1.95
2.07
2.00
2.03
0.87
3.00
3.31
3.18
f1 (ppm)

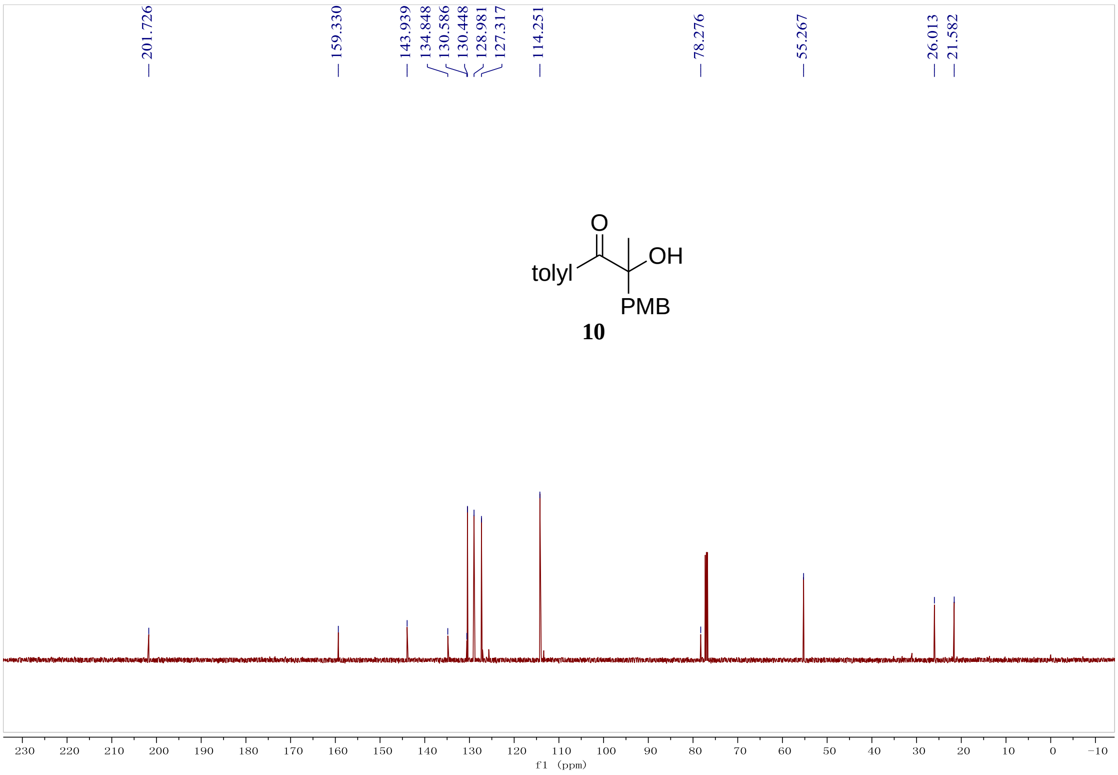

201.726
159.330
143.939
134.848
130.586
130.448
128.981
127.317
114.251
78.276
55.267
26.013
21.582
O
tolyl
OH
PMB
230 220 210 200 190 180 170 160 150 140 130 120 110 100 90 80 70 60 50 40 30 20 10 0 −10
f1 (ppm)

10

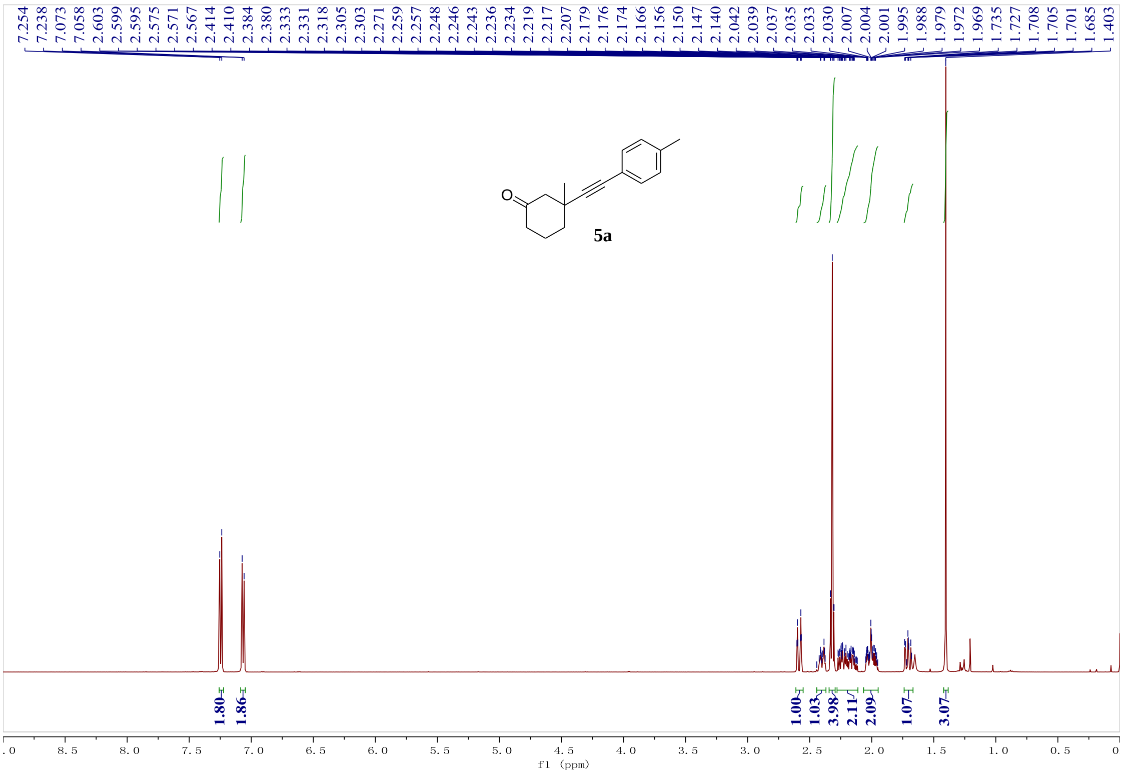
7.254
7.238
7.073
7.058
2.603
2.599
2.595
2.575
2.571
2.567
2.414
2.410
2.384
2.380
2.333
2.331
2.318
2.305
2.303
2.271
2.259
2.257
2.248
2.246
2.243
2.236
2.234
2.219
2.217
2.207
2.179
2.176
2.174
2.166
2.156
2.150
2.147
2.140
2.042
2.039
2.037
2.035
2.033
2.030
2.007
2.004
2.001
1.995
1.988
1.979
1.972
1.969
1.735
1.727
1.708
1.705
1.701
1.685
1.403
O
5a
1.80
1.86
1.00
1.03
3.98
2.11
2.09
1.07
3.07
f1 (ppm)

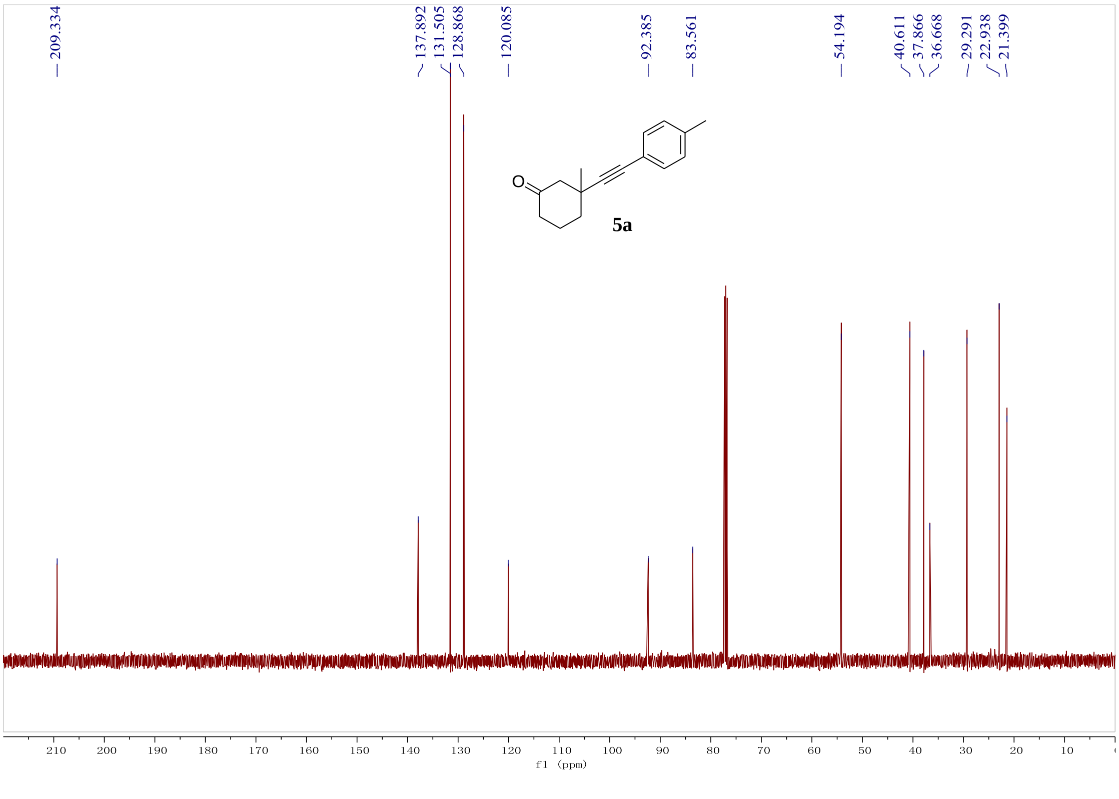
209.334
137.892
131.505
128.868
120.085
92.385
83.561
54.194
40.611
37.866
36.668
29.291
22.938
21.399
O
5a
f1 (ppm)

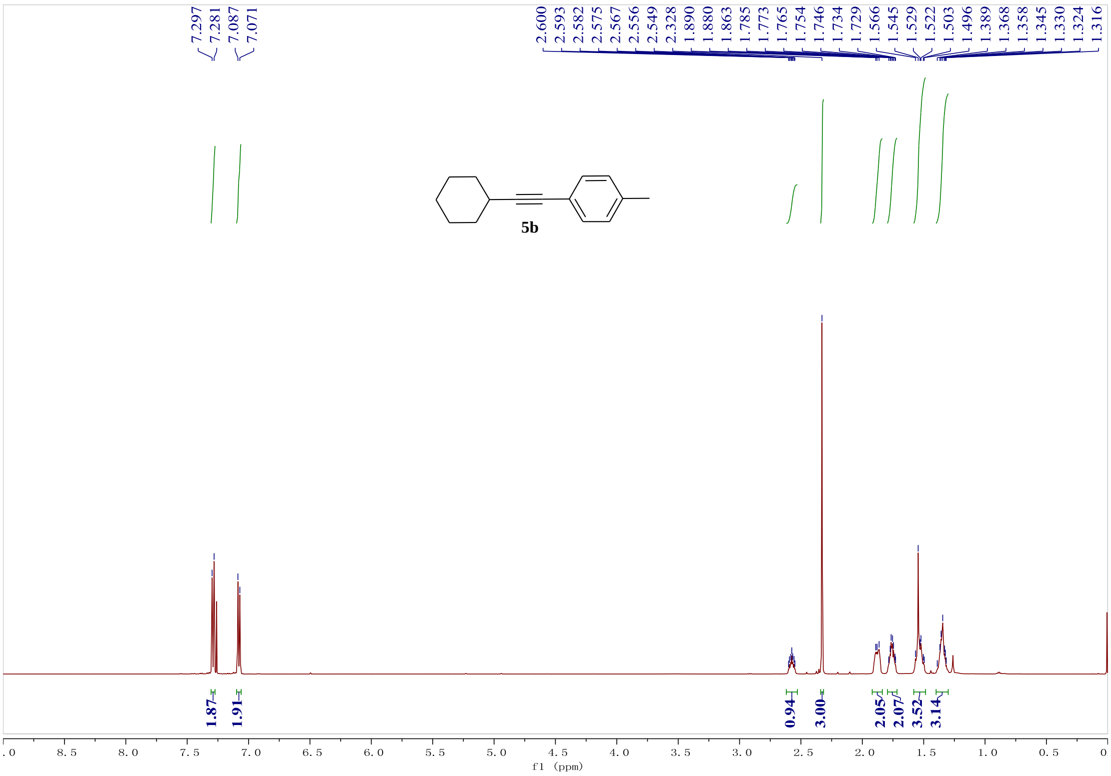
7.297
7.281
7.087
7.071
2.600
2.593
2.582
2.575
2.567
2.556
2.549
2.328
1.890
1.880
1.863
1.785
1.773
1.765
1.754
1.746
1.734
1.729
1.566
1.545
1.529
1.522
1.503
1.496
1.389
1.368
1.358
1.345
1.330
1.324
1.316
5b
1.87
1.91
0.94
3.00
2.05
2.07
3.52
3.14
f1 (ppm)

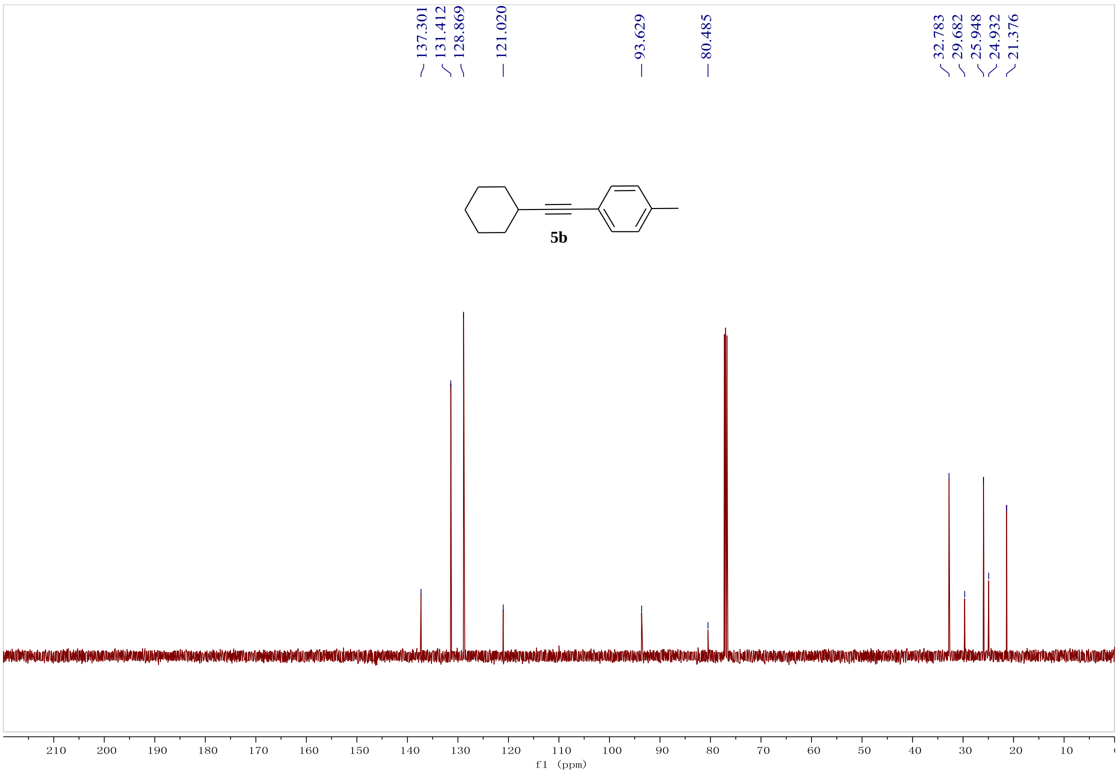
137.301
131.412
128.869
121.020
93.629
80.485
32.783
29.682
25.948
24.932
21.376
5b
f1 (ppm)

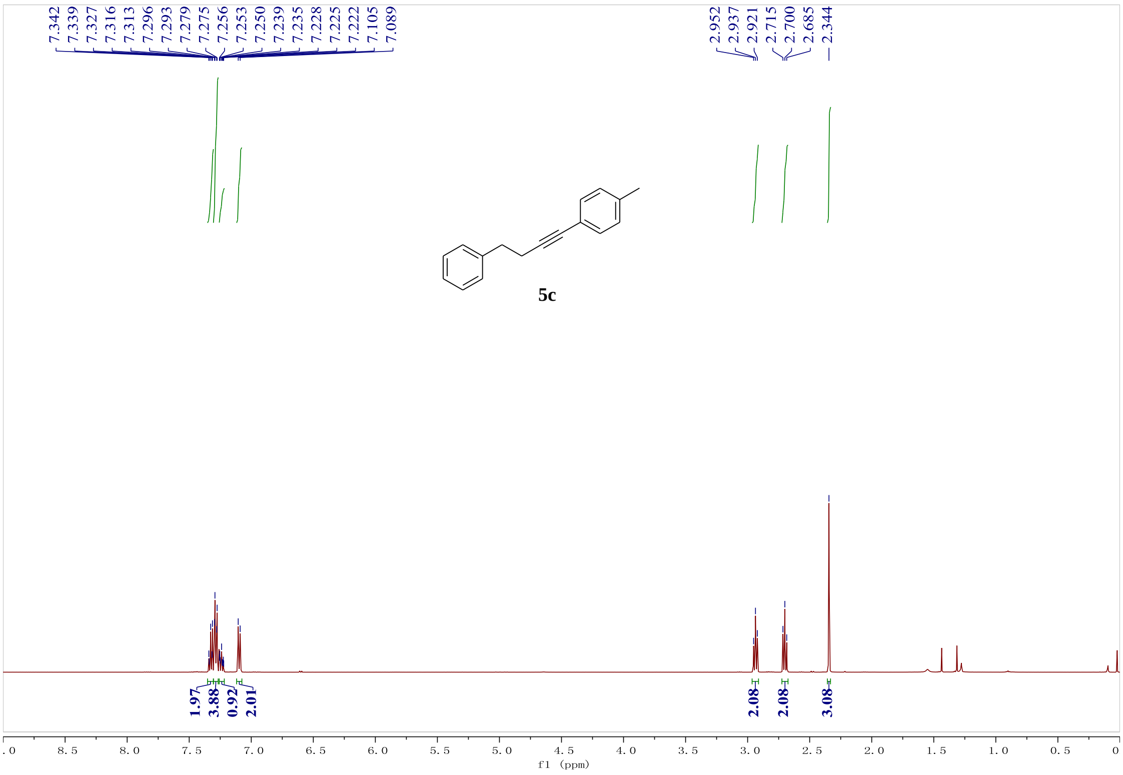
7.342
7.339
7.327
7.316
7.313
7.296
7.293
7.279
7.275
7.256
7.253
7.250
7.239
7.235
7.228
7.225
7.222
7.105
7.089
2.952
2.937
2.921
2.715
2.700
2.685
2.344
5c
1.97
3.88
0.92
2.01
2.08
2.08
3.08
f1 (ppm)

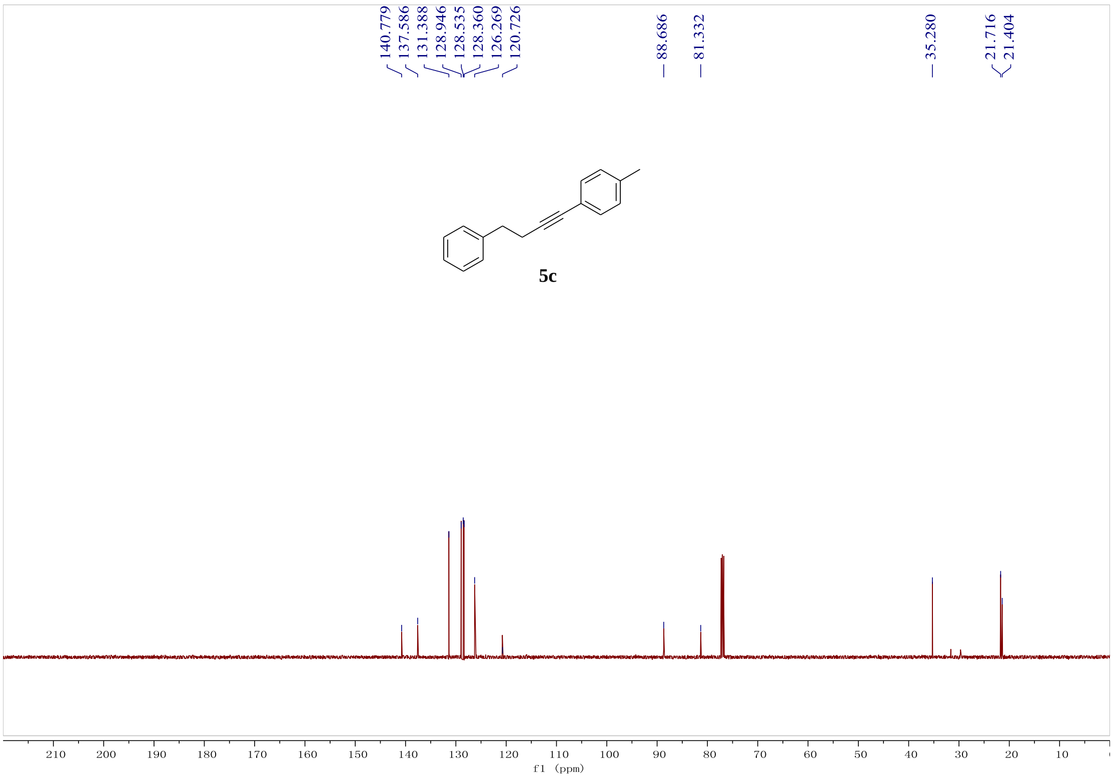
140.779
137.586
131.388
128.946
128.535
128.360
126.269
120.726
88.686
81.332
35.280
21.716
21.404
5c
f1 (ppm)

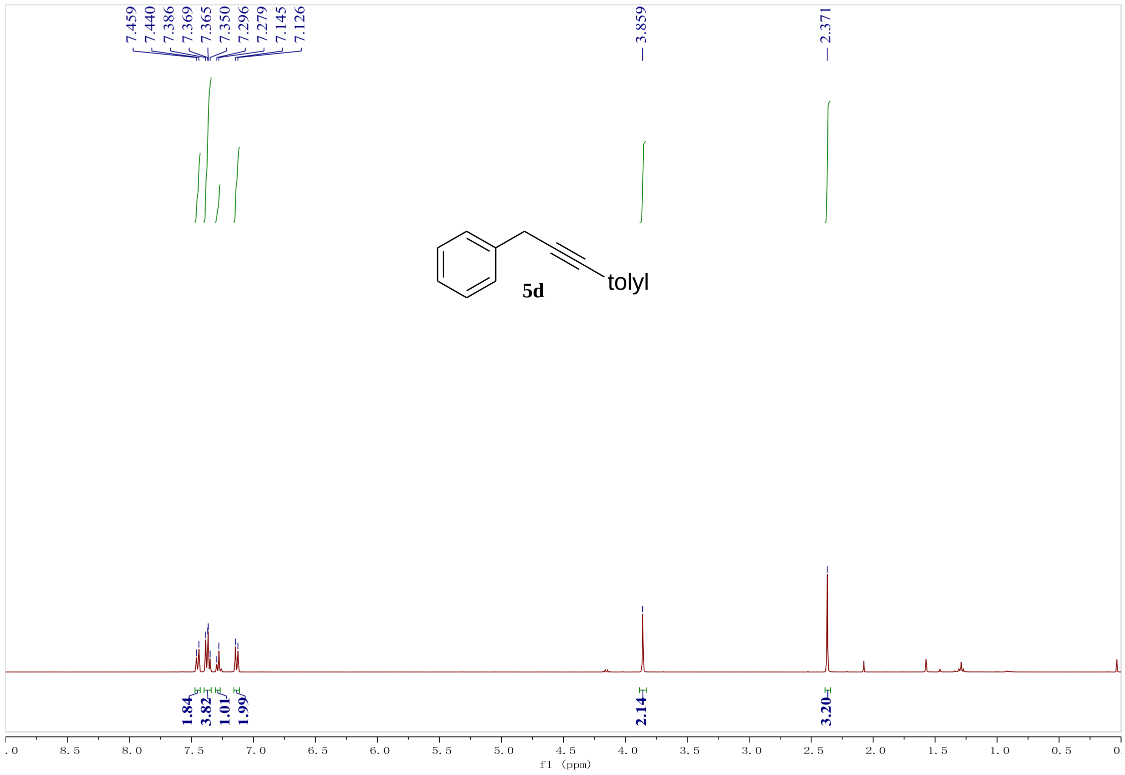
7.459
7.440
7.386
7.369
7.365
7.350
7.296
7.279
7.145
7.126
3.859
2.371
5d
tolyl
1.84
3.82
1.01
1.99
2.14
3.20
f1 (ppm)

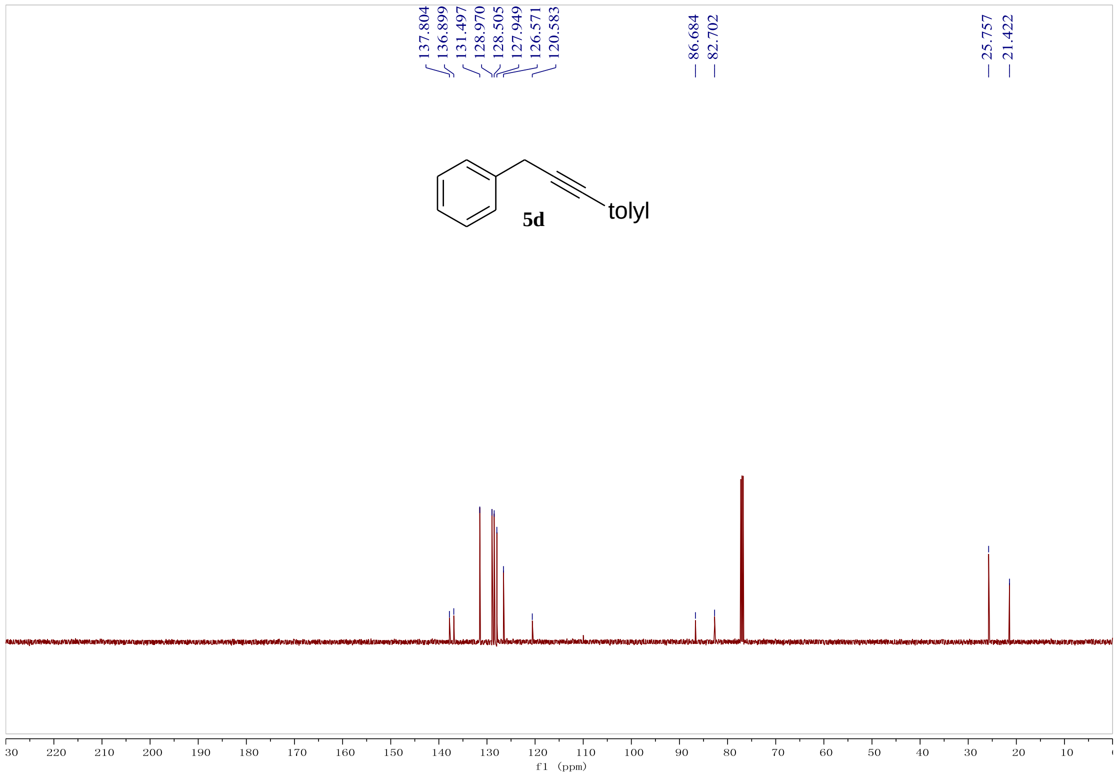
137.804
136.899
131.497
128.970
128.505
127.949
126.571
120.583
86.684
82.702
25.757
21.422
5d
tolyl
f1 (ppm)

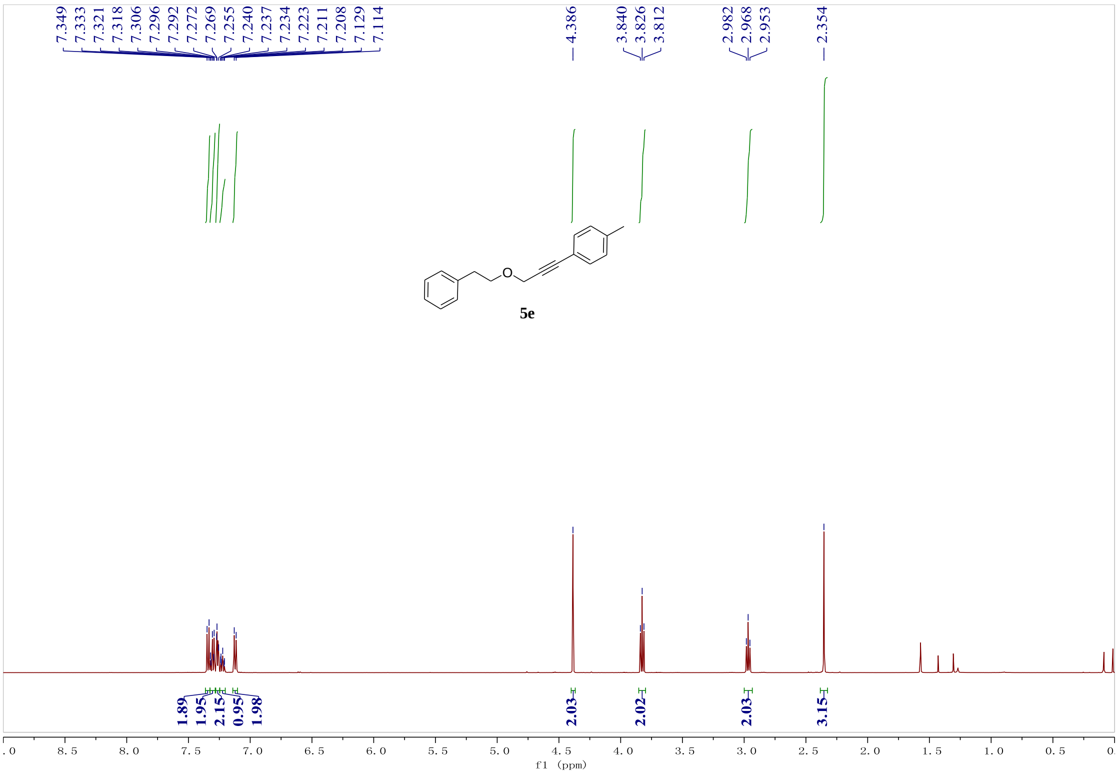
7.349
7.333
7.321
7.318
7.306
7.296
7.292
7.272
7.269
7.255
7.240
7.237
7.234
7.223
7.211
7.208
7.129
7.114
4.386
3.840
3.826
3.812
2.982
2.968
2.953
2.354
5e
1.89
1.95
2.15
0.95
1.98
2.03
2.02
2.03
3.15
f1 (ppm)

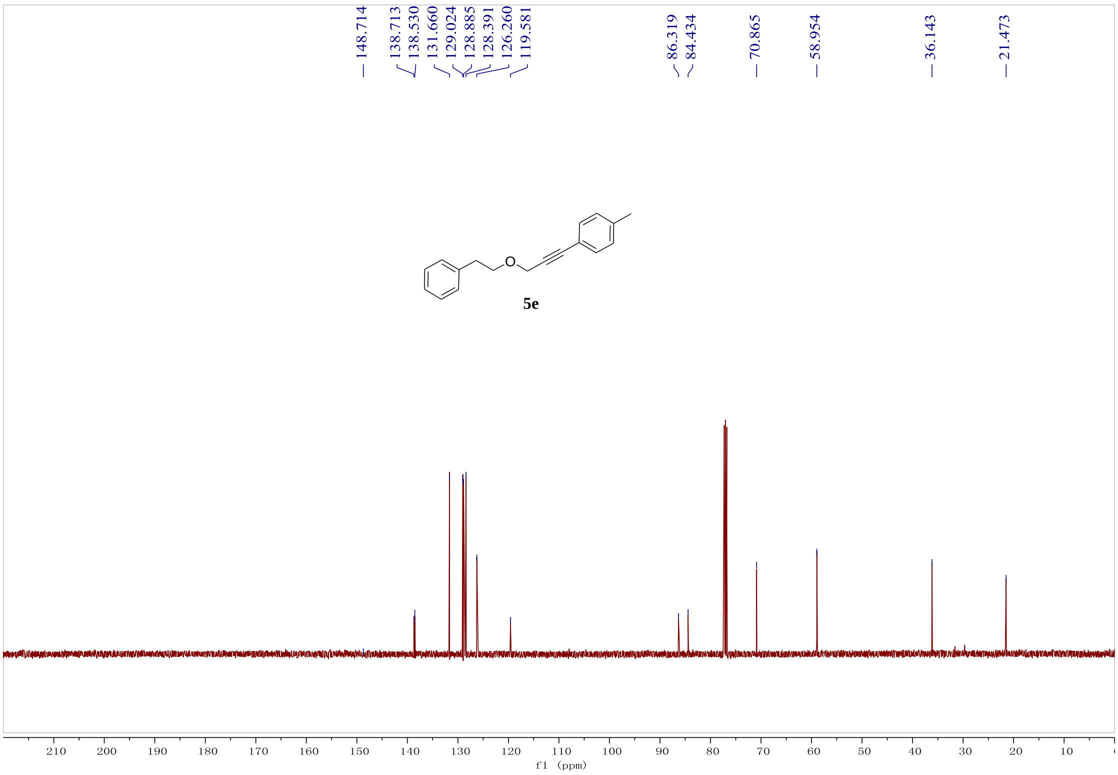
148.714
138.713
138.530
131.660
129.024
128.885
128.391
126.260
119.581
86.319
84.434
70.865
58.954
36.143
21.473
5e
f1 (ppm)

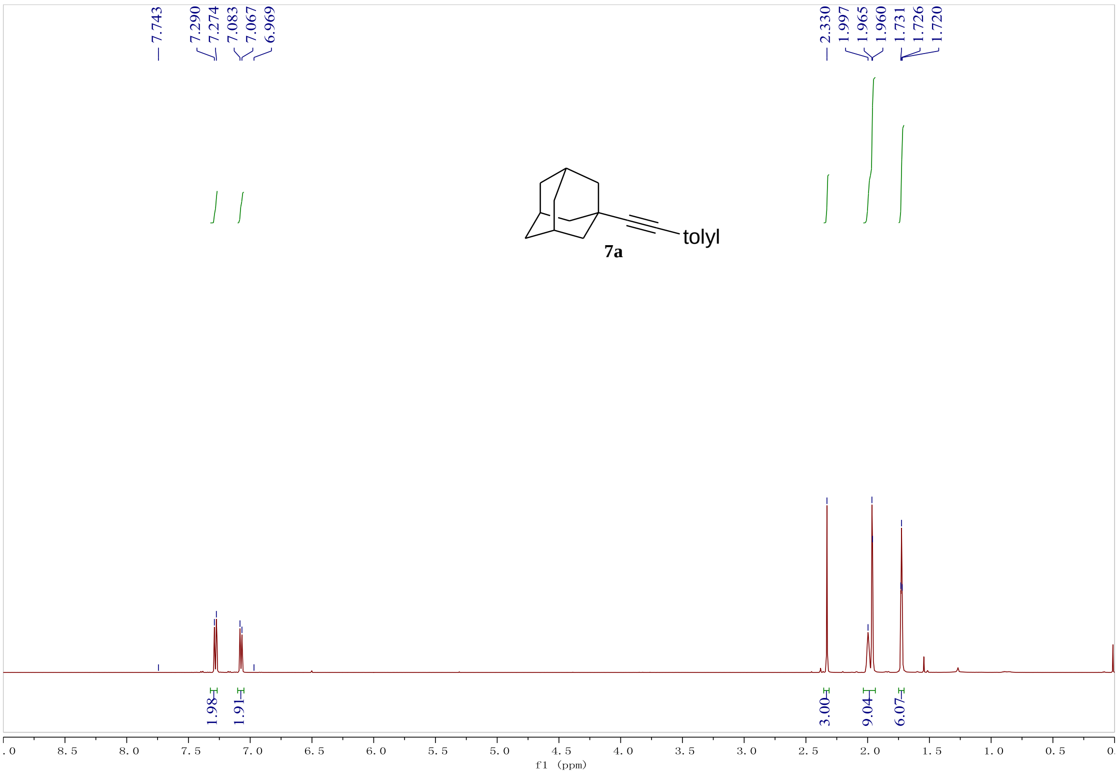
7.743
7.290
7.274
7.083
7.067
6.969
2.330
1.997
1.965
1.960
1.731
1.726
1.720
tolyl
7a
1.98
1.91
3.00
9.04
6.07
f1 (ppm)

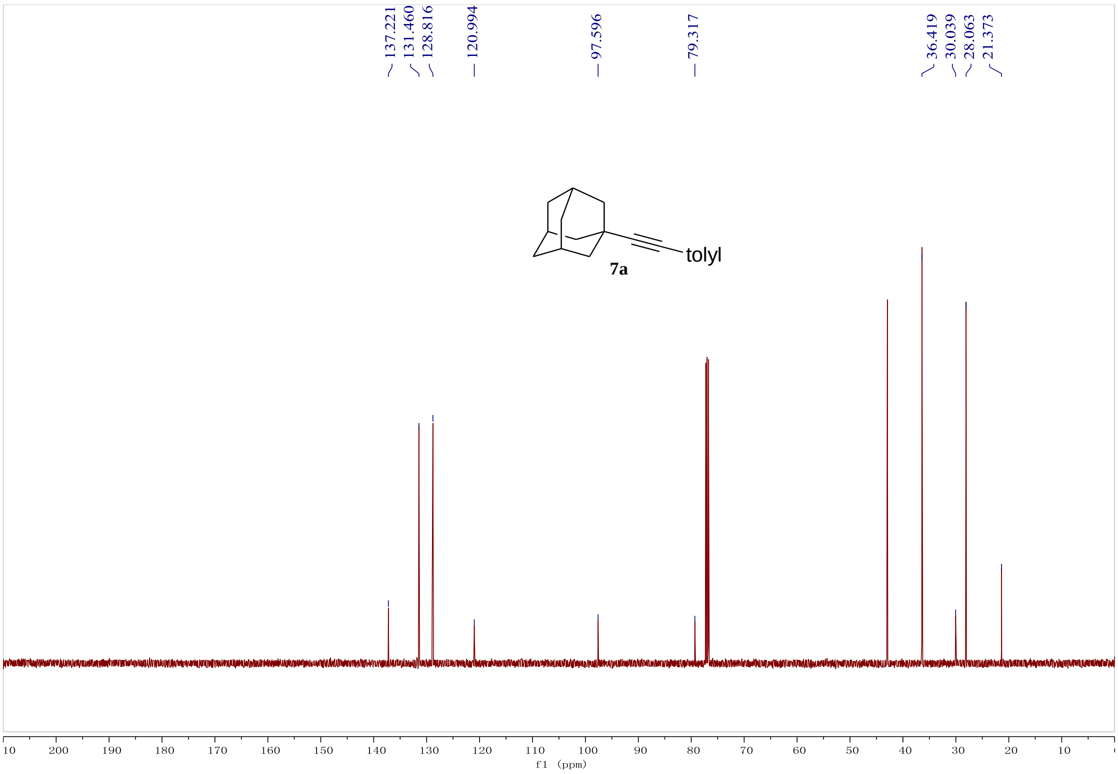
137.221
131.460
128.816
120.994
97.596
79.317
36.419
30.039
28.063
21.373
tolyl
7a
f1 (ppm)

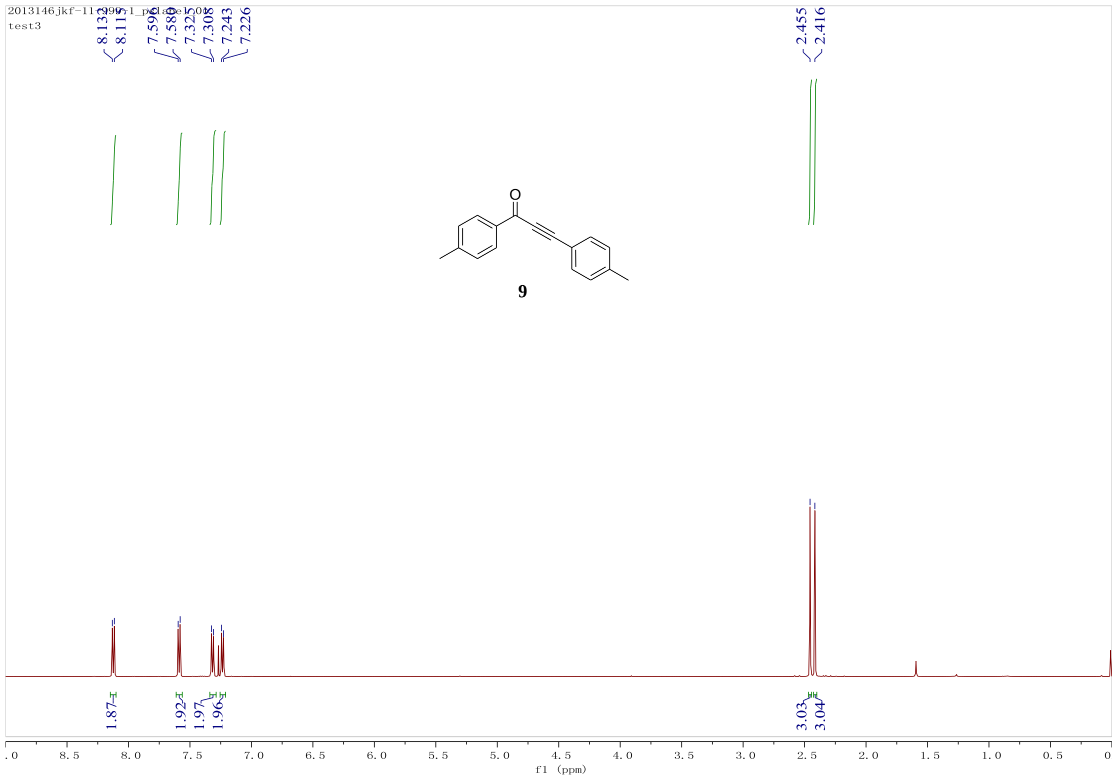
test3
8.132
8.115
7.596
7.580
7.325
7.308
7.243
7.226
2.455
2.416
O
9
1.87
1.92
1.97
1.96
3.03
3.04
f1 (ppm)

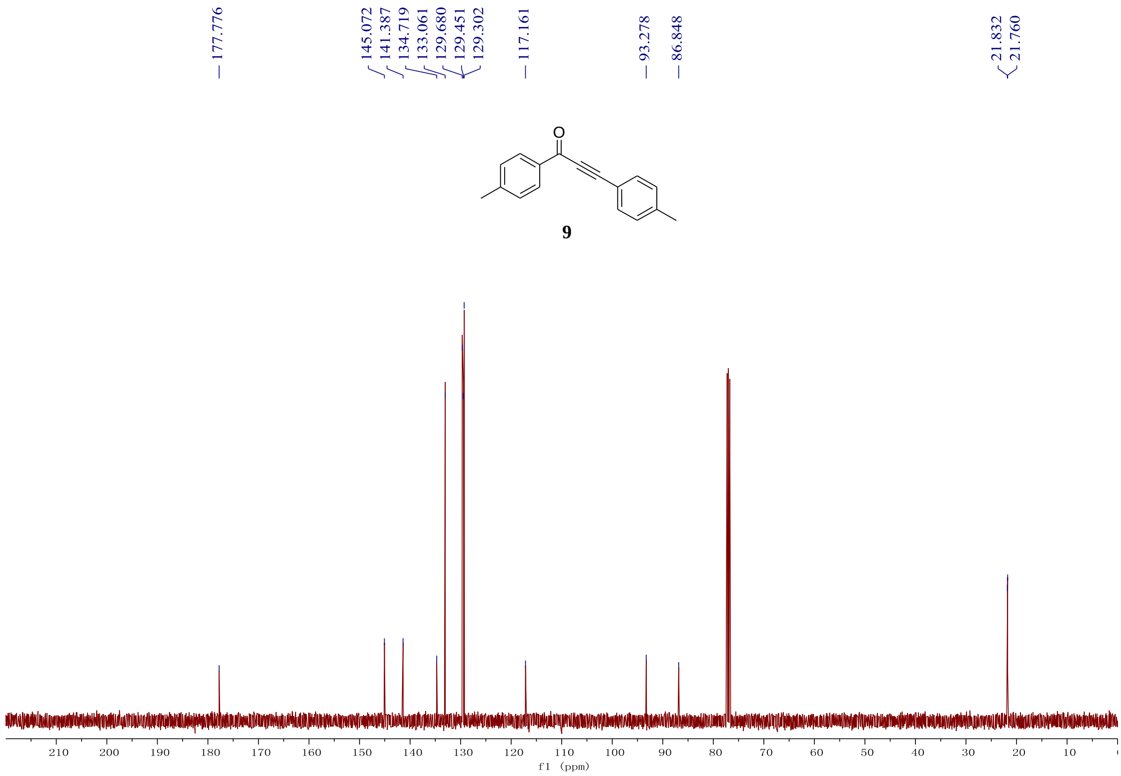
177.776
145.072
141.387
134.719
133.061
129.680
129.451
129.302
117.161
93.278
86.848
21.832
21.760
O
9
f1 (ppm)
